# Updated Insights on Cardiac and Vascular Risks of Proton Pump Inhibitors: A Real-World Pharmacovigilance Study

**DOI:** 10.3389/fcvm.2022.767987

**Published:** 2022-02-25

**Authors:** Yinghong Zhai, Xiaofei Ye, Fangyuan Hu, Jinfang Xu, Xiaojing Guo, Zhen Lin, Xiang Zhou, Zhijian Guo, Yang Cao, Jia He

**Affiliations:** ^1^School of Medicine, Tongji University, Shanghai, China; ^2^Department of Health Statistics, Second Military Medical University, Shanghai, China; ^3^Department of Medical Service, Naval Hospital of Eastern Theater, Zhoushan, China; ^4^Clinical Epidemiology and Biostatistics, School of Medical Sciences, Örebro University, Örebro, Sweden

**Keywords:** cardiac, vascular, proton pump inhibitors, FAERS database, disproportionality analysis

## Abstract

**Background:**

Proton pump inhibitors (PPIs) are among the most widely prescribed medications in clinical practice. However, there are also concerns about the potential risks of long-term PPI use. The present study aimed to examine the safety of PPIs and summarize their potential cardiac and vascular risks in a real-world setting.

**Methods:**

This pharmacovigilance study extracted records between January 2015 and December 2019 from the FDA Adverse Event Reporting System (FAERS) database. The association of seven PPI medications with cardiac and vascular events (CVEs) were evaluated. Two established pharmacovigilance methods, reporting odds ratio (ROR) and information components (IC) based statistical shrinkage, were used to measure disproportionality.

**Results:**

In total 62,140 CVE records associated with PPI use were investigated. Women showed a higher proportion (54.37%) of PPI-associated CVEs. The median time from PPI initiation to CVE onset was 97 [interquartile range (IQR): 8–491] days, with the shortest median time of 42 days (IQR: 2–277 days) for esomeprazole, and the longest time of 389 days (IQR: 0–525 days) for dexlansoprazole. Although PPIs were not associated with elevated CVE risks compared those of the whole database (IC_025_/ROR_025_ = −0.39/0.74), various signals emerged. Despite some similarities exist between the PPIs, their cardiac and vascular safety profiles varied significantly. Pantoprazole showed the broadest spectrum of signals, from thrombotic thrombocytopenic purpura (IC_025_/ROR_025_ = 0.01/1.08) to renal haemangioma (IC_025_/ROR_025_ = 3.14/9.58). Esomeprazole showed the second-broadest spectrum of toxicities, ranging from duodenal ulcer hemorrhage (IC_025_/ROR_025_ = 0.07/1.28) to hypertensive nephropathy (IC_025_/ROR_025_ = 4.09/18.72). Vascular signals were more dominant than cardiac signals, suggesting that vascular function was more heavily affected. Hypertensive nephropathy, renal haemangioma, renal artery stenosis, and renal infarct had strong signals across most PPI regimens and merited further attention.

**Conclusions:**

PPIs may inflict various CVEs, particularly those involving the vascular system, on the users. Given the wide range of onset times and different toxicity profiles for various PPI medications, they should be prescribed with caution.

## Introduction

Proton pump inhibitors (PPIs) are a class of effective medications used to treat various acid-related disorders. Their use in the clinical setting has increased rapidly and tremendously (1). PPIs are among the most commonly used medications worldwide (2). However, the widespread availability of these agents has also contributed to inappropriate prescriptions and medication overuse. Currently, the administration of PPIs is increasing in daily clinical practice, and there is growing concern regarding the potential long-term risks associated with these agents (3). Mounting clinical data have suggested that chronic exposure to PPIs increases the susceptibility of patient to serious adverse sequelae, including gastric cancer (4), fractures (5), kidney disorders (6), cardiovascular events (7), and dementia (8).

Among these complications, cardiac and vascular events (CVEs) have garnered considerable attention because of their potentially fatal effects. Although there are several published studies on this issue, the findings are controversial. Moreover, existing real-world evidence on the cardiovascular safety profile of different PPIs is scarce despite their wide PPIs are widely use in clinical settings. Therefore, it is vital to assess their overall adverse cardiovascular risks. Herein, the current study aimed to evaluate the real-world pattern of cardiac and vascular adverse events (AEs) among PPI users and summarize and prioritize signals that warrant further attention.

## Methods

### Study Design and Data Sources

The observational pharmacovigilance study used a subset of the Food and Drug Administration (FDA) Adverse Events Reporting System (FAERS) database of the United States between January 2015 and December 2019. All authors had access to the study data and reviewed and approved the final manuscript.

The FAERS database gathers worldwide reports related to AEs and medication errors that are spontaneously submitted by healthcare professionals, patients, and pharmaceutical manufacturers (9). It is maintained by the FDA and supports the FDA's ongoing public health safety policy for the surveillance of medications and therapeutic biologic products (10).

The FAERS database is publicly available and permits the analysis of a large quantity of data to identify safety signals. The potential of FAERS in detecting early safety issues has been reported previously, especially those on newly approved medications (11) and rare AEs (12). All data in this study can be accessed at fis.fda.gov/extensions/FPD-QDE-FAERS/FPD-QDE-FAERS.html.

### Procedures

Before the formal analysis, we conducted data deduplication and standardization procedures. First, we scrutinized the extracted data based on similarities among demographic characteristics (sex and age), name of the medication, and AE-related information (type of AE, starting date, reporting year, country of reporter, event date, end date, and outcome) to detect and eliminate duplicate records. In addition, we excluded records whose start dates of drug therapy were later than the dates of AE (13). The onset time was defined as the period between the start date of PPI initiation to the onset of a CVE, which was calculated using the variables EVENT_DT and START_DT in the FAERS database.

Seven PPI medications (omeprazole, lansoprazole, pantoprazole, rabeprazole, ilaprazole, esomeprazole, and dexlansoprazole) were investigated in the current study. We used generic and brand names to identify PPI-related records. In FAERS, AEs in each record are coded using the preferred term (PT) according to the Medical Dictionary for Regulatory Activities (MedDRA). A given PT can be assigned to one or more high-level terms (HLTs), high-level group terms (HLGTs), and system organ class (SOC) levels (14). The multiaxial nature of MedDRA provides flexibility in AE retrieval. In the study, we identified potential records of interest using all PTs related to cardiac disorders (SOC code: 10007541) and vascular disorders (SOC code: 10047065) according to MedDRA (*version 22.0*).

### Statistical Analysis

We used the disproportionality analysis to compare the frequency of selected AEs reported for a single drug or a class of drugs with the frequency of the same AEs reported for other medications in the FAERS database (15). Disproportionality emerges when the reporting frequency of a specific AE for a given medication is higher than that in the background data. In our study, we used the two established pharmacovigilance indices, reporting odds ratio (ROR) and information components (IC), to measure disproportionality (16, 17).

Calculations for ROR and IC were based on 2 × 2 contingency table (Supplementary Table S1), in which we compared the proportion of the targeted AEs reported for the selected medications (single or a class of medications) with the proportion of the same AEs reported for the control group, i.e. the whole database used. In addition, we applied statistical shrinkage (shrinkage parameter = 0.5) to reduce the number of false-positive signals when calculating ROR or IC (18). Formulas were presented as follows:


$$\begin{array}{l} \text{ROR}=\left(N_{observed}+0.5\right)/\left(N_{expected}+0.5\right) \end{array}$$

$$\begin{array}{l} IC=\log_{2}\left(\left(N_{observed}+0.5\right)/\left(N_{expected}+0.5\right)\right) \end{array}$$

$$\begin{array}{l} N_{expected}=\left(n_{drug}*\;n_{event}\right)/n_{total} \end{array}$$

$$\begin{array}{r} N_{expected}\;:\text{ the number of records expected for the selected}\\ \text{medication}-\text{AE combination}. \end{array}$$

$$\begin{array}{r} N_{observed}\;:\text{ the observed number of records for the selected}\\ \text{medication}-\text{AE combinations}. \end{array}$$

$$\begin{array}{l} n_{drug}\;:\text{ the total number of records for the selected medication}. \end{array}$$

$$\begin{array}{l} n_{event}\;:\text{ the total number of total records for the selected AE}. \end{array}$$

$$\begin{array}{l} n_{total}\;:\text{ the total number of records in the database}. \end{array}$$

A signal emerged if the lower limits of the 95% confidence intervals of ROR (ROR_025_) and IC (IC_025_) exceeded the predefined thresholds (1 and 0, respectively) in at least three records. All analyses were conducted using SAS 9.4 software (SAS Institute, Cary, NC).

## Results

### Descriptive Results

**Figure 1:**
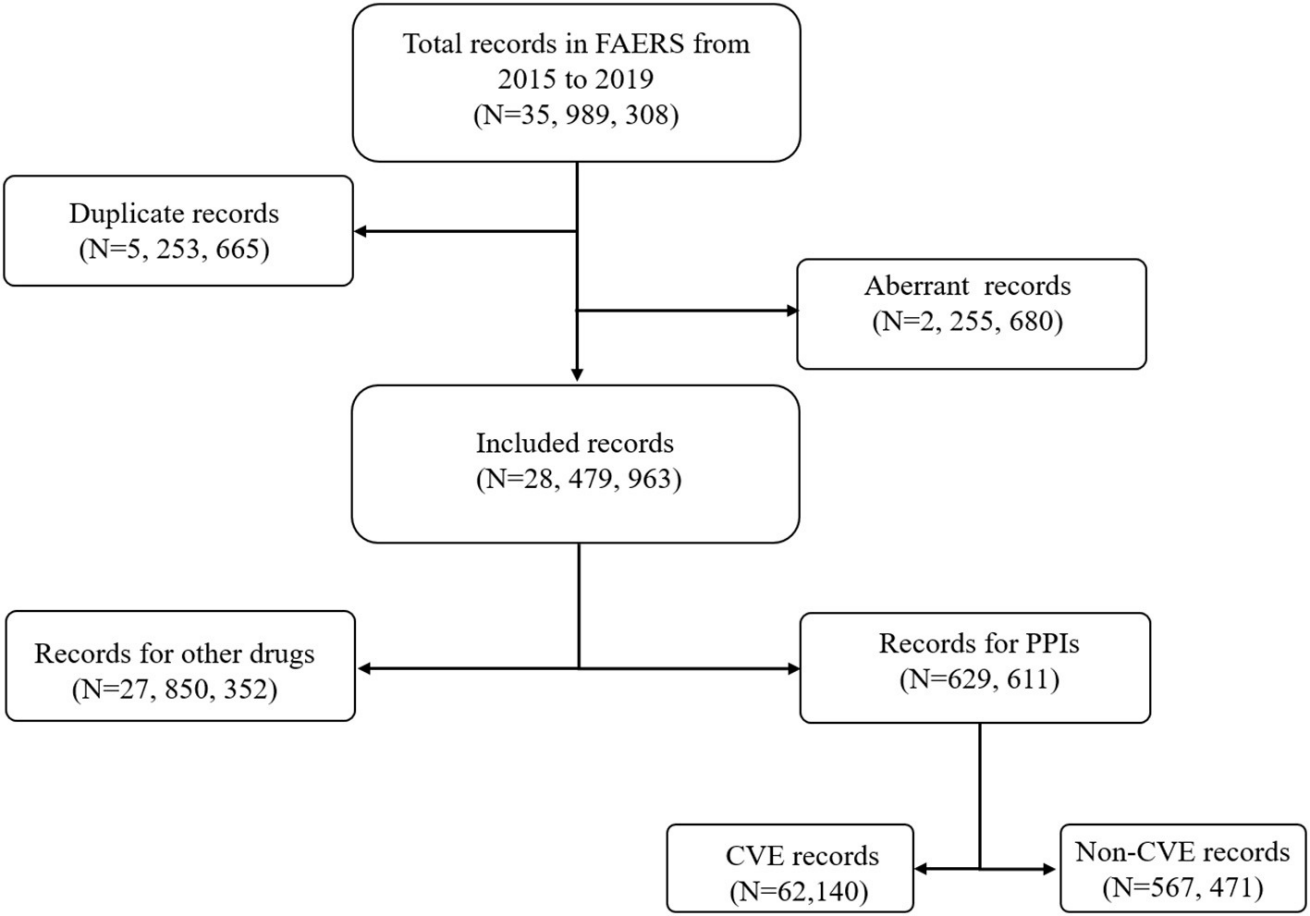
Flow chart of the record selection process from the U.S. food and drug administration adverse events reporting system database.

After removing the duplicate (*N* = 5,253,665) and aberrant records (*N* = 2,255,680), a total of 28,479,963 records were included in the final analysis (Figure 1). Among these, 62,140 records of PPI-associated CVEs were identified.

The characteristics of these records were presented in Table 1. In general, we observed a growing trend in the number of CVE records over the study period, which reflects the continually increased use of PPIs. Omeprazole was the most frequently prescribed PPI (*n* = 25,713; 41.38%), followed by pantoprazole (*n* = 20,653; 33.24%). We did not identify any CVE records for ilaprazole because it is not an FDA-approved PPI and, consequently, was rarely used. Therefore, ilaprazole was excluded from the further analysis and not shown in Table 1. Overall, women showed a greater proportion of PPI-associated CVEs than men (54.37 vs. 45.63%). The trend remained in PPI-specific analyses. The mean age at CVE onset was 64.79 years across all records in which this demographic information was available. The median onset time of CVEs was 97 days (interquartile range (IQR): 8–491 days) after PPI initiation, with the shortest median time of 42 days (IQR: 2–277 days) for esomeprazole, and the longest time of 389 days (IQR: 0–525 days) for dexlansoprazole. Hospitalisations accounted for 48.78% of all associated records and the proportion of death was 14.4%. Pantoprazole was associated with the largest proportion of hospitalization and rabeprazole was related to the largest proportion of death.

**Table 1:** Characteristics of PPI-associated CVE records in FAERS from 2015 to 2019.

**Characteristics**	**Total PPIs**	**Omeprazole**	**Lansoprazole**	**Pantoprazole**	**Rabeprazole**	**Esomeprazole**	**Dexlansoprazole**
**Characteristics**	**(*N =* 62,140)**	**(*N =* 25,713)**	**(*N =* 8,846)**	**(*N =* 20,653)**	**(*N =* 2,250)**	**(*N =* 3141)**	**(*N =* 1,537)**
**Sex**							
Male	26,261 (45.63)	10,615 (44.17)	3,785 (47.71)	9,189 (47.75)	940 (44.57)	1,343 (46.29)	389 (29.31)
Female	31,286 (54.37)	13,418 (55.83)	4,149 (52.29)	10,054 (52.25)	1,169 (55.43)	1,558 (53.71)	938 (70.69)
Data available	57,547	24,033	7,934	19,243	2,109	2,901	1,327
**Age**							
mean ±SD, years	64.79 ±16.07	63.87 ± 16.41	65.68 ± 17.44	65.75 ± 15.06	67.21 ± 14.40	63.29 ± 16.48	60.23 ± 15.21
Data available	48,267	19,787	6,821	1,6295	1,914	2,520	930
**Year**							
2015	11,093 (17.85)	5,128 (19.94)	1,553 (17.56)	3,305 (16.00)	458 (20.36)	410 (13.05)	239 (15.55)
2016	11,475 (18.47)	5,096 (19.82)	1,571 (17.76)	3,622 (17.54)	445 (19.78)	470 (14.96)	271 (17.63)
2017	10,318 (16.60)	4,373 (17.01)	1,400 (15.83)	3,468 (16.79)	400 (17.78)	487 (15.50)	190 (12.36)
2018	14,080 (22.66)	5,536 (21.53)	1,985 (22.44)	4,892 (23.69)	482 (21.42)	754 (24.01)	431 (28.04)
2019	15,174 (24.42)	5,580 (21.70)	2,337 (26.42)	5,366 (25.98)	465 (20.67)	1,020 (32.47)	406 (26.42)
Data available	62,140	25,713	8,846	20,653	2,250	3,141	1,537
**Reported countries**							
United States	30,818 (49.59)	15,674 (60.96)	2,679 (30.28)	10,159 (49.19)	398 (17.69)	764 (24.32)	1,144 (74.43)
Great Britain	5,344 (8.60)	2,758 (10.73)	2,018 (22.81)	325 (1.57)	41 (1.82)	201 (6.40)	1 (0.07)
Canada	3,535 (5.69)	495 (1.93)	319 (3.61)	1,901 (9.20)	413 (18.36)	172 (5.48)	235 (15.29)
Germany	3,302 (5.31)	408 (1.59)	20 (0.23)	2,765 (13.39)	11 (0.49)	98 (3.12)	0 (0.00)
Japan	3,243 (5.22)	314 (1.22)	1,875 (21.20)	4 (0.02)	913 (40.58)	137 (4.36)	0 (0.00)
Italy	2,602 (4.19)	721 (2.80)	799 (9.03)	852 (4.13)	75 (3.33)	154 (4.90)	1 (0.07)
Others	13,296 (21.40)	5,343 (20.78)	1,136 (12.84)	4,647 (22.50)	399 (17.73)	1,615 (51.42)	156 (10.15)
Data available	62,140	25,713	8,846	20,653	2250	3,141	1,537
**Time to onset**							
Median, days	97	122	100	72	173	42	389
Quartile 1–3	8–491	10–579	10–470	8–389	24–713	2–277	0–525
Data available	8,004	2,969	1,535	2,497	399	473	131
**Outcome**							
Death	8,007 (14.40)	2,995 (13.69)	1,345 (16.20)	2,718 (14.26)	371 (17.72)	464 (15.37)	114 (9.08)
Life-threatening	4,070 (7.32)	1,521 (6.95)	684 (8.24)	1,320 (6.93)	212 (10.12)	275 (9.11)	58 (4.62)
Disability	1,422 (2.56)	596 (2.72)	279 (3.36)	351 (1.84)	87 (4.15)	82 (2.72)	27 (2.15)
Hospitalization	27,122 (48.78)	10,643 (48.64)	3,467 (41.77)	10,300 (54.05)	874 (41.74)	1,408 (46.65)	430 (34.24)
Congenital anomaly	92 (0.17)	31 (0.14)	5 (0.06)	40 (0.21)	0 (0.00)	16 (0.53)	0 (0.00)
Other serious (important medical event)	14,872 (26.75)	6,092 (27.84)	2,508 (30.22)	4,322 (22.68)	550 (26.27)	773 (25.61)	627 (49.92)
Required intervention	21 (0.04)	5 (0.02)	12 (0.14)	4 (0.02)	0 (0.00)	0 (0.00)	0 (0.00)
Data available	55,606	21,883	8,300	19,055	2,094	3,018	1,256

*CVE, cardiac and vascular event; PPI, proton pump inhibitor*.

### Disproportionality

**Figure 2:**
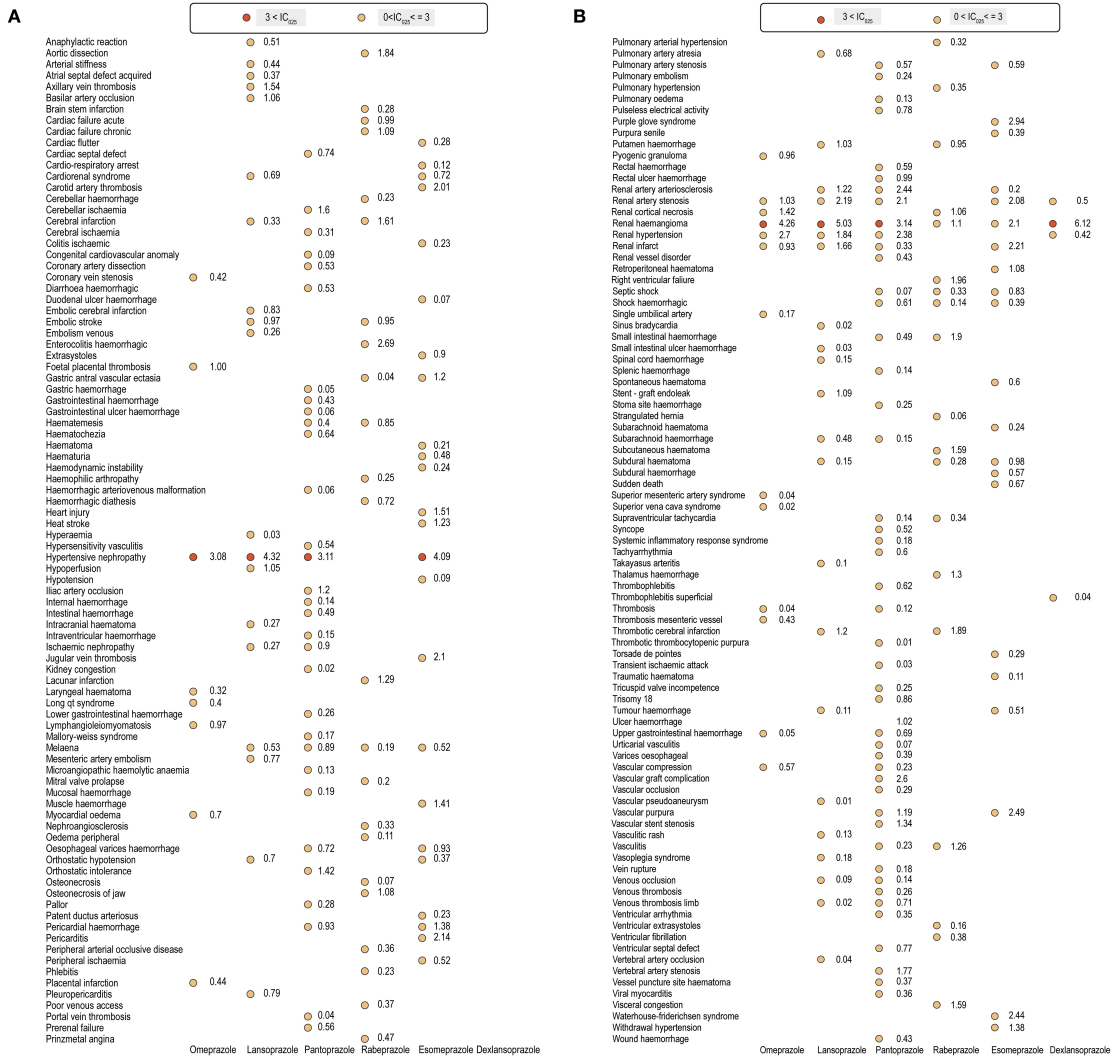
**(A,B)** Cardiac and vascular toxicity profiles of different proton pump inhibitor regimens.

**Figure 3:**
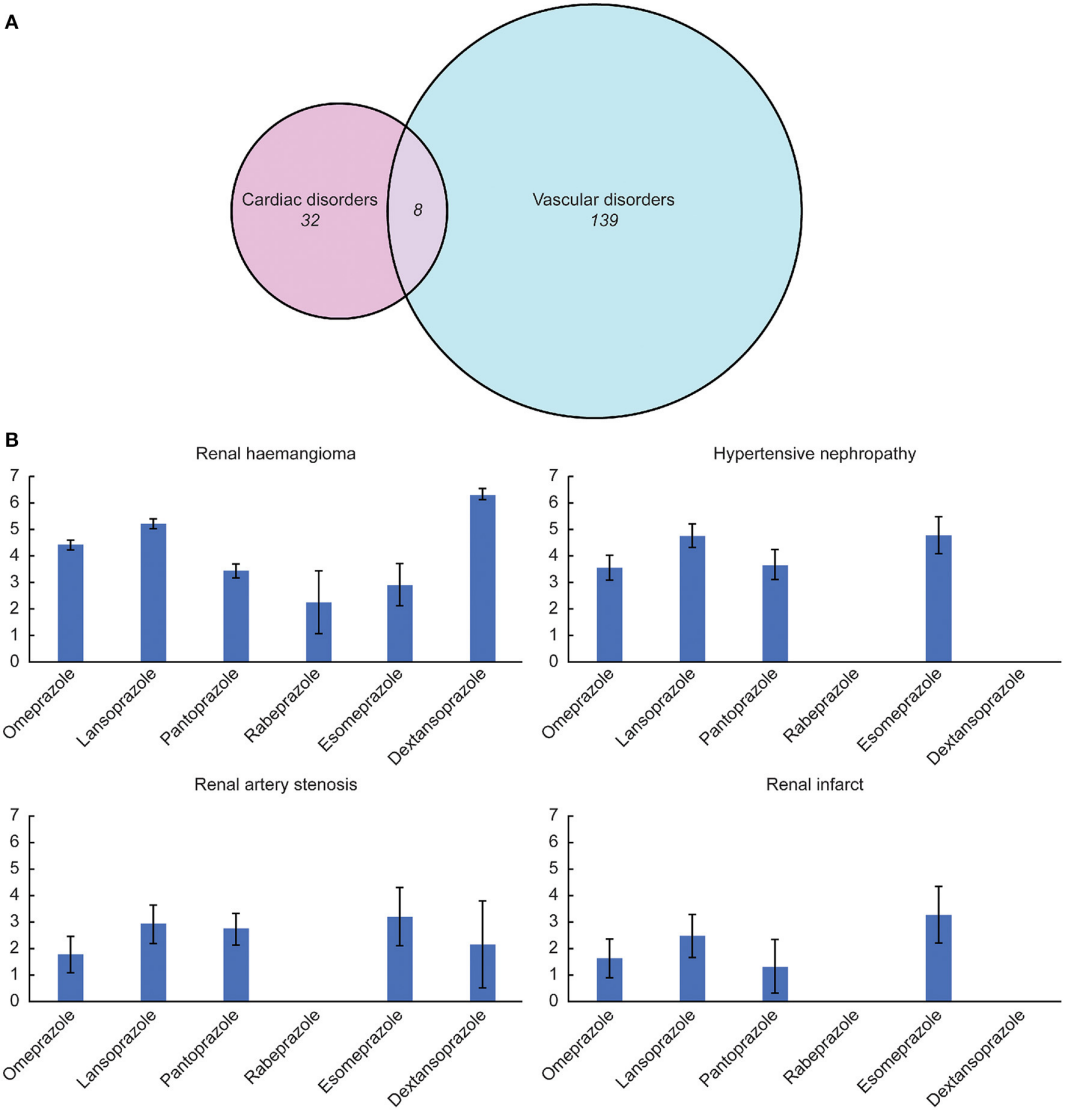
**(A)** Composition proportion of the identified signals. **(B)** Association between several highlighted adverse events and different proton pump inhibitor drugs quantified by the information components value.

No over-reporting of CVEs was identified in patients who received PPIs (total or specific) compared with all patients in database (Table 2). However, after further data mining for the PPIs at the PT level, we observed a wide range of cardiac and vascular signals (Figure 2). Notably, most of the emerging signals were associated vascular disorders, while cardiac signals constituted a small proportion (Figure 3). Details of the disproportionality analysis are presented in the Supplementary Tables S2–S7.

**Table 2:** Results of the disproportionality analysis for PPI-associated CVEs (total and specific).

**Drug**	**N**	**IC**	**IC_**025**_**	**IC_**975**_**	**ROR**	**ROR_**025**_**	**ROR_**975**_**
PPIs	62,140	−0.37	−0.39	−0.36	0.74	0.74	0.75
Omeprazole	25,713	−0.43	−0.45	−0.41	0.71	0.70	0.72
Lansoprazole	8,846	−0.67	−0.70	−0.63	0.60	0.58	0.61
Pantoprazole	20,653	−0.09	−0.11	−0.06	0.93	0.92	0.95
Rabeprazole	2,250	−0.19	−0.26	−0.12	0.86	0.82	0.90
Ilaprazole	0	−2.89			0.12		
Esomeprazole	3,141	−0.14	−0.20	−0.08	0.90	0.86	0.93
Dexlansoprazole	1,537	−1.34	−1.43	−1.26	0.36	0.34	0.38

*CVEs, cardiac and vascular events; N, number of records; IC, information components; ROR, reporting odds ratio; IC_025_/IC_975_, the lower/upper limit of 95% confidence interval for the IC; PPI, proton pump inhibitors; ROR_025_/ROR_975_, the lower/upper limit of 95% confidence interval for the ROR*.

Despite several overlapping signals, the cardiac and vascular toxicity profiles differed significantly among different PPI regimens. Pantoprazole showed the broadest spectrum of toxicities, ranging from thrombotic thrombocytopenic purpura (IC_025_/ROR_025_ = 0.01/1.08) to renal haemangioma (IC_025_/ROR_025_ = 3.14/9.58). Esomeprazole showed the second-broadest spectrum of toxicities, ranging from duodenal ulcer hemorrhage (IC_025_/ROR_025_ = 0.07/1.28) to hypertensive nephropathy (IC_025_/ROR_025_ = 4.09/18.72), which was closely followed by rabeprazole, ranging from gastric antral vascular ectasia (IC_025_/ROR_025_ = 0.04/1.62) to enterocolitis haemorrhagic (IC_025_/ROR_025_ = 2.69/7.26). Notably, hypertensive nephropathy and renal haemangioma emerged as the strongest signals across several PPIs.

## Discussion

PPIs have been a cornerstone for the treatment of several acid-related diseases for the past few years. PPIs are one of the most commonly prescribed medications in clinical practice, and their overall use has increased dramatically since the 1990s. However, concerns regarding the inappropriate use and overdosing of PPIs are also growing simultaneously (19–21). Due to the wide application of PPIs, many unanticipated AEs have also been reported. Numerous articles have raised alarm about the long-term use of PPIs due to their wide range of potential risks. Cardiovascular AEs have been particularly regarded for patients administered PPIs. However, the currently available studies present contradictory findings on this issue.

Based on real-world clinical evidence, there is a growing need for comprehensive and accurate safety profiles for CVEs in patients taking PPIs. The current study is the largest and most extensive characterization of potentially PPI-associated CVEs by mining the FAERS database to date. By analyzing a large number of records, we were able to detect signals for even relatively rare AEs. We enumerate the notable and interesting findings as follows:

1. Our study depicted the clinical characteristics of CVE records for total and specific PPI drugs. We noted significant differences in the times to CVEs onset according to different PPI regimens. Overall, the median time from initiation of PPIs to onset for CVEs was 97 days (IQR: 8–491 days). Esomeprazole and dexlansoprazole exhibited the shortest and longest median onset time, respectively. Notably, we found that CVEs might either occur shortly after PPI intake or manifest in PPI recipients after several years. Similarly, a recent pharmacovigilance study reported that patients in different PPI regimens showed a significant difference in the median time to acute kidney injury onset (22). Therefore, we suggest that the short- and long-term regimens of PPIs need to be carefully prescribed, considering the wide range of onset times of different PPIs. Additionally, more people are using PPIs for longer durations than what is recommended by clinical guidelines (19, 23), and many AEs are potentially associated with chronic PPI exposure (24–26). Vigilant administration of PPI drugs is needed. It is highly recommended that when PPIs are applied in long-term therapy, periodic assessment is necessary to reduce potential cardiac and vascular complications (27).
2. Existing studies on the association between cardiovascular disorders and PPIs are conflicting. Several studies showed that PPIs were associated with an augmented risk of cardiovascular events (28–30), while others found no this association and supported the cardiovascular safety profile of PPIs (31–33). Notably, in the present study, we examined and quantified all potential cardiac and vascular risks after PPI treatment in a real-world setting, which cast a light on a comprehensive and detailed understanding of this safety issue. Our results indicated that CVEs were not over-reported in PPI users when compared with the whole FAERS database. However, we found several disease signals when conducting further data mining at the PT level. Despite many cardiac manifestations, such as cardiac failure acute/chronic, pericarditis, right ventricular failure, and heart injury, being detected with significant disproportionality, most of the signals were related to vascular disorders.

PPIs might adversely affect vascular and cardiac physiology in multiple ways. PPI-associated vascular dysfunction has gained increasing attention and proposed mechanisms have been investigated in several studies. Ghebremariam et al. found that PPIs can reduce the activity of dimethylarginine dimethylamino-hydrolase (DDAH), thereby increasing the plasma level of asymmetrical dimethylarginine (ADMA), an endogenous and competitive inhibitor of nitric oxide synthase (NOS). Elevated ADMA may attenuate the vasoprotective effects of endothelial NOS and increase the risk of vascular inflammation and thrombosis (34). Additionally, Yepuri et al. found that chronic exposure to PPIs can speed endothelial aging, which seemed a result of an inhibition of lysosomal acidification and subsequent disruption of proteostasis (35).

Taken together, such a disruption of vascular homeostasis and broad impairment in endothelial function may cause vascular disorders. As a result, long-term exposure to PPIs exposed patients to an augmented risk of cardiac diseases, which may explain the increased cardiovascular events (36). Our finding that vascular signals were overwhelmingly dominant compared to cardiac signals also supports that vascular function is much more directly and heavily affected than cardiac function. Future in-depth research is needed to reveal the underlying mechanism in this process.

3. We discovered that although some similarities exist between the PPIs, their cardiac and vascular safety profiles varied significantly. Very few published studies have assessed the cardiac and vascular toxicities of different PPIs. Our study may address this information gap by presenting and characterizing all disproportionate signals observed after the use of different PPIs. We found that pantoprazole showed the broadest spectrum of cardiac and vascular signals, followed by esomeprazole. Notably, we identified a significant disproportionality among several vascular disorders involving the renal system, particularly hypertensive nephropathy, renal haemangioma, renal artery stenosis, and renal infarcts. In recent years, PPIs have come under scrutiny for a rising number of associated AEs related to the renal system. The elevated risk of chronic kidney diseases and their progression among PPI users have been emphasized in numerous epidemiological studies (37–39).

Several studies have reported the association between PPI treatment and acute kidney injury (40–42). Our study supplements this existing literature by revealing novel potential kidney AEs following PPI use. We also observed some new disproportionate associations in specific PPI drugs. Taken together, our findings suggest that since the cardiac and vascular risk profiles of PPIs are significantly different, physicians should consider the potential risks of each PPI and select the optimal drug for individual patients.

There were several limitations in the present study that should be recognized. First, our analysis had inevitable and unquantifiable bias due to the nature of the spontaneous reporting mechanism of the FAERS database, such as over-reporting, under-reporting, and missing variable values in large proportions. Second, due to the absence of the total number of patients exposed to PPIs in FAERS, we could not calculate AE incidence and establish a causal relationship. Lastly, although the disproportionality calculation methods are efficient and popular in hypotheses generation, they fall short of controlling for confounding factors, such as the masking effect and co-prescription (43). Notwithstanding the limitations mentioned above, our findings from this extensive analysis of a large database generated and prioritized several AE signals that merit further investigation. Our work provides the scientific community with several important clues for future in-depth clinical research.

## Conclusions

Patients undergoing PPI treatment may experience cardiac and vascular AEs, with a higher possibility of vascular disorders. It is important to understand that despite some similarities, the safety profiles of various PPI medications regarding CVEs are significantly different. As the population exposure to PPIs is expected to continually rise *en masse*, a comprehensive understanding of the toxicity profile of different PPIs is needed. Caution should be exercised when selecting optimal PPI medications for corresponding indications, and the likelihood of potential cardiac and vascular complications should be considered.

## Data Availability Statement

Publicly available datasets were analyzed in this study. This data can be found here: https://fis.fda.gov/extensions/FPD-QDE-FAERS/FPD-QDE-FAERS.html.

## Author Contributions

YZ, XY, and FH: conception and design of the work and analysis and interpretation of data. YZ and FH: acquisition and review of data. JX and XG: categorization and verification of data. YZ: drafting the article. YC, XY, and FH: manuscript reviewing. All authors contributed to the article and approved the submitted version.

## Funding

This study was supported by the National Nature Science Foundation of China (No. 82073671), the Leading Talents of Public Health in Shanghai (No. GWV-10.2-XD22), the Shanghai Municipal Commission of Health and Family Planning Fund for Excellent Young Scholars (No. 2018YQ47), the Excellent Young Scholars of Public Health in Shanghai (No. GWV-10.2-YQ33), three-year Action Program of Shanghai Municipality for Strengthening the Construction of Public Health System (GWV-10.1-XK05), Big Data and Artificial Intelligence Application, and Military Key Discipline Construction Project (Health Service-Naval Health Service Organization and Command) (No. 03).

## Conflict of Interest

The authors declare that the research was conducted in the absence of any commercial or financial relationships that could be construed as a potential conflict of interest.

## Publisher's Note

All claims expressed in this article are solely those of the authors and do not necessarily represent those of their affiliated organizations, or those of the publisher, the editors and the reviewers. Any product that may be evaluated in this article, or claim that may be made by its manufacturer, is not guaranteed or endorsed by the publisher.

## Abbreviations

ADMA: asymmetrical dimethylarginine
AE: adverse event
CVE: cardiac and vascular event
DDAH: dimethylarginine dimethylamino-hydrolase
FDA: Food and Drug Administration (FDA)
FAERS: Food and Drug Administration adverse event reporting system
HLGT: high-level group term
HLT: high-level term
IC: information component
IQR: interquartile range
MedDRA: Medical Dictionary for Regulatory Activities
NOS: nitric oxide synthase
PPI: proton pump inhibitor
PT: preferred term
ROR: reporting odds ratio
SOC: system organ class.
