# Supplementary material for: Updated Insights on Cardiac and Vascular Risks of Proton Pump Inhibitors: A Real-World Pharmacovigilance Study
Source: Front Cardiovasc Med. 2022 Feb 25;9:767987. doi: 10.3389/fcvm.2022.767987 (PMC8913586; doi:10.3389/fcvm.2022.767987)
Supplement: Supplementary file 1 [file Data_Sheet_1.docx]

**Table S1**. 2 × 2 contingency table for the calculation of ROR and IC*

|  | Target AEs | Other AEs |
| --- | --- | --- |
| Selected medications | a | b |
| Other medications | c | d |

* a: the number of records for the selected medication-AE combinations; b: the number of records with any other AEs reported for the selected medications; c: the number of records with any target AEs for other medications; d: the number of records reported other AEs for other medications.

**Tables S2**-**Table S7** provide the detailed disproportionality results of cardiac and vascular toxicities associated with PPI drugs, respectively. Only PTs with at least 3 records were listed. AEs with records below 3 in class-specific PPIs were not calculated the signal values. IC_025_: the lower limit of a 95% CI for the IC; IC_975_: the upper limit of a 95% CI; ROR_025_: the lower limit of the 95%CI of ROR; ROR_975_: the upper limit of the 95%CI of ROR. IC_025_>0 and ROR_025_ >1 was deemed a signal. N: number of records; a: the number of records with CVEs reported for specific PPI; b: the number of records with any other AEs reported for specific PPI; c: the number of records with any CVEs for other drugs; d: the number of records reported other AEs for other drugs.

**Table** **S2.** Disproportionality results of cardiac and vascular toxicities associated with omeprazole

| PT | a | b | c | d | IC | IC_025_ | IC_975_ | ROR | ROR_025_ | ROR_975_ |
| --- | --- | --- | --- | --- | --- | --- | --- | --- | --- | --- |
| Dyspnoea | 1399 | 269413 | 216750 | 27992401 | -0.57 | -0.66 | -0.48 | 0.67 | 0.64 | 0.71 |
| Dizziness | 1114 | 269698 | 171722 | 28037429 | -0.56 | -0.66 | -0.46 | 0.67 | 0.64 | 0.72 |
| Peripheral swelling | 1053 | 269759 | 103538 | 28105613 | 0.08 | -0.02 | 0.18 | 1.06 | 1.00 | 1.13 |
| Gastrointestinal haemorrhage | 878 | 269934 | 117891 | 28091260 | -0.36 | -0.47 | -0.25 | 0.78 | 0.73 | 0.83 |
| Syncope | 741 | 270071 | 74574 | 28134577 | 0.05 | -0.07 | 0.17 | 1.04 | 0.96 | 1.11 |
| Hypertension | 723 | 270089 | 87441 | 28121710 | -0.21 | -0.34 | -0.09 | 0.86 | 0.80 | 0.93 |
| Pulmonary embolism | 701 | 270111 | 69374 | 28139777 | 0.07 | -0.05 | 0.20 | 1.05 | 0.98 | 1.13 |
| Thrombosis | 681 | 270131 | 62999 | 28146152 | 0.17 | 0.04 | 0.30 | 1.13 | 1.04 | 1.21 |
| Myocardial infarction | 625 | 270187 | 95705 | 28113446 | -0.55 | -0.68 | -0.42 | 0.68 | 0.63 | 0.74 |
| Hypotension | 542 | 270270 | 86926 | 28122225 | -0.62 | -0.76 | -0.48 | 0.65 | 0.60 | 0.71 |
| Rectal haemorrhage | 499 | 270313 | 47503 | 28161648 | 0.13 | -0.02 | 0.28 | 1.09 | 1.00 | 1.20 |
| Chest pain | 496 | 270316 | 67515 | 28141636 | -0.38 | -0.53 | -0.23 | 0.77 | 0.70 | 0.84 |
| Palpitations | 485 | 270327 | 52284 | 28156867 | -0.05 | -0.20 | 0.10 | 0.97 | 0.88 | 1.06 |
| Cerebrovascular accident | 446 | 270366 | 77680 | 28131471 | -0.74 | -0.89 | -0.58 | 0.60 | 0.54 | 0.66 |
| Oedema peripheral | 436 | 270376 | 49959 | 28159192 | -0.14 | -0.29 | 0.02 | 0.91 | 0.83 | 1.00 |
| Renal haemangioma | 434 | 270378 | 1648 | 28207503 | 4.42 | 4.26 | 4.58 | 26.66 | 23.98 | 29.64 |
| Upper gastrointestinal haemorrhage | 424 | 270388 | 38103 | 28171048 | 0.21 | 0.05 | 0.37 | 1.16 | 1.05 | 1.28 |
| Tachycardia | 323 | 270489 | 44324 | 28164827 | -0.39 | -0.58 | -0.21 | 0.76 | 0.68 | 0.85 |
| Transient ischaemic attack | 317 | 270495 | 30075 | 28179076 | 0.13 | -0.05 | 0.32 | 1.10 | 0.98 | 1.23 |
| Migraine | 302 | 270510 | 42281 | 28166870 | -0.42 | -0.61 | -0.23 | 0.74 | 0.66 | 0.83 |
| Septic shock | 281 | 270531 | 38110 | 28171041 | -0.38 | -0.57 | -0.18 | 0.77 | 0.68 | 0.86 |
| Haemorrhage | 255 | 270557 | 44436 | 28164715 | -0.74 | -0.94 | -0.53 | 0.60 | 0.53 | 0.68 |
| Epistaxis | 251 | 270561 | 39810 | 28169341 | -0.60 | -0.81 | -0.39 | 0.66 | 0.58 | 0.74 |
| Atrial fibrillation | 245 | 270567 | 44832 | 28164319 | -0.81 | -1.02 | -0.59 | 0.57 | 0.50 | 0.65 |
| Pulmonary oedema | 236 | 270576 | 27547 | 28181604 | -0.16 | -0.38 | 0.05 | 0.89 | 0.79 | 1.01 |
| Deep vein thrombosis | 232 | 270580 | 33014 | 28176137 | -0.45 | -0.66 | -0.23 | 0.73 | 0.64 | 0.83 |
| Chest discomfort | 216 | 270596 | 31976 | 28177175 | -0.50 | -0.73 | -0.28 | 0.70 | 0.62 | 0.80 |
| Anaphylactic reaction | 208 | 270604 | 25480 | 28183671 | -0.23 | -0.46 | 0.00 | 0.85 | 0.74 | 0.97 |
| Lower gastrointestinal haemorrhage | 201 | 270611 | 17715 | 28191436 | 0.24 | 0.00 | 0.47 | 1.18 | 1.03 | 1.36 |
| Cardiac failure congestive | 189 | 270623 | 42989 | 28166162 | -1.12 | -1.36 | -0.88 | 0.46 | 0.40 | 0.53 |
| Haematochezia | 187 | 270625 | 24282 | 28184869 | -0.31 | -0.56 | -0.07 | 0.80 | 0.69 | 0.93 |
| Hot flush | 186 | 270626 | 27843 | 28181308 | -0.52 | -0.76 | -0.28 | 0.70 | 0.60 | 0.80 |
| Cardiac disorder | 177 | 270635 | 32206 | 28176945 | -0.80 | -1.05 | -0.55 | 0.57 | 0.49 | 0.66 |
| Contusion | 172 | 270640 | 35166 | 28173985 | -0.96 | -1.22 | -0.71 | 0.51 | 0.44 | 0.59 |
| Skin ulcer | 172 | 270640 | 18141 | 28191010 | -0.02 | -0.27 | 0.23 | 0.99 | 0.85 | 1.15 |
| Vaginal haemorrhage | 171 | 270641 | 26836 | 28182315 | -0.59 | -0.84 | -0.33 | 0.66 | 0.57 | 0.77 |
| Flushing | 168 | 270644 | 30197 | 28178954 | -0.78 | -1.04 | -0.52 | 0.58 | 0.50 | 0.67 |
| Subdural haematoma | 165 | 270647 | 20523 | 28188628 | -0.25 | -0.51 | 0.00 | 0.84 | 0.72 | 0.98 |
| Haematuria | 163 | 270649 | 23103 | 28186048 | -0.44 | -0.70 | -0.18 | 0.74 | 0.63 | 0.86 |
| Cardiac failure | 156 | 270656 | 38226 | 28170925 | -1.22 | -1.49 | -0.96 | 0.43 | 0.36 | 0.50 |
| Cardiac arrest | 151 | 270661 | 31261 | 28177890 | -0.98 | -1.25 | -0.71 | 0.50 | 0.43 | 0.59 |
| Haemoptysis | 150 | 270662 | 18331 | 28190820 | -0.23 | -0.50 | 0.04 | 0.85 | 0.73 | 1.00 |
| Internal haemorrhage | 142 | 270670 | 19564 | 28189587 | -0.40 | -0.68 | -0.12 | 0.76 | 0.64 | 0.89 |
| Orthostatic hypotension | 135 | 270677 | 12389 | 28196762 | 0.18 | -0.11 | 0.47 | 1.13 | 0.96 | 1.34 |
| Presyncope | 129 | 270683 | 15032 | 28194119 | -0.16 | -0.45 | 0.13 | 0.89 | 0.75 | 1.06 |
| Acute myocardial infarction | 127 | 270685 | 21805 | 28187346 | -0.71 | -1.01 | -0.42 | 0.61 | 0.51 | 0.72 |
| Ischaemic stroke | 127 | 270685 | 18109 | 28191042 | -0.45 | -0.74 | -0.15 | 0.73 | 0.61 | 0.87 |
| Injection site haemorrhage | 121 | 270691 | 24785 | 28184366 | -0.97 | -1.27 | -0.66 | 0.51 | 0.43 | 0.61 |
| Osteonecrosis of jaw | 121 | 270691 | 26502 | 28182649 | -1.06 | -1.36 | -0.76 | 0.48 | 0.40 | 0.57 |
| Melaena | 116 | 270696 | 15195 | 28193956 | -0.33 | -0.63 | -0.02 | 0.80 | 0.66 | 0.96 |
| Injection site bruising | 113 | 270699 | 29457 | 28179694 | -1.31 | -1.62 | -1.00 | 0.40 | 0.33 | 0.48 |
| Haematemesis | 109 | 270703 | 12323 | 28196828 | -0.12 | -0.43 | 0.20 | 0.92 | 0.76 | 1.11 |
| Ventricular tachycardia | 106 | 270706 | 12697 | 28196454 | -0.20 | -0.52 | 0.12 | 0.87 | 0.72 | 1.05 |
| Haemorrhage intracranial | 102 | 270710 | 13734 | 28195417 | -0.37 | -0.69 | -0.04 | 0.77 | 0.64 | 0.94 |
| Cerebral haemorrhage | 100 | 270712 | 23948 | 28185203 | -1.19 | -1.52 | -0.86 | 0.44 | 0.36 | 0.53 |
| Pulmonary hypertension | 100 | 270712 | 14312 | 28194839 | -0.45 | -0.78 | -0.12 | 0.73 | 0.60 | 0.89 |
| Pericardial effusion | 96 | 270716 | 12190 | 28196961 | -0.28 | -0.62 | 0.06 | 0.82 | 0.67 | 1.00 |
| Dyspnoea exertional | 92 | 270720 | 14951 | 28194200 | -0.63 | -0.98 | -0.29 | 0.64 | 0.52 | 0.79 |
| Cardio-respiratory arrest | 91 | 270721 | 17138 | 28192013 | -0.84 | -1.19 | -0.50 | 0.55 | 0.45 | 0.68 |
| Pulmonary thrombosis | 87 | 270725 | 7418 | 28201733 | 0.28 | -0.07 | 0.64 | 1.22 | 0.99 | 1.51 |
| Pulmonary arterial hypertension | 84 | 270728 | 13808 | 28195343 | -0.65 | -1.01 | -0.29 | 0.63 | 0.51 | 0.79 |
| Supraventricular tachycardia | 83 | 270729 | 7764 | 28201387 | 0.15 | -0.21 | 0.52 | 1.11 | 0.90 | 1.38 |
| Haemorrhagic stroke | 80 | 270732 | 10078 | 28199073 | -0.27 | -0.64 | 0.10 | 0.83 | 0.66 | 1.03 |
| Bradycardia | 79 | 270733 | 19738 | 28189413 | -1.25 | -1.62 | -0.87 | 0.42 | 0.34 | 0.52 |
| Pallor | 79 | 270733 | 8713 | 28200438 | -0.08 | -0.46 | 0.29 | 0.94 | 0.76 | 1.18 |
| Haematoma | 78 | 270734 | 14675 | 28194476 | -0.84 | -1.22 | -0.47 | 0.56 | 0.44 | 0.69 |
| Sudden death | 75 | 270737 | 11023 | 28198128 | -0.49 | -0.87 | -0.11 | 0.71 | 0.57 | 0.89 |
| Coronary artery disease | 74 | 270738 | 13727 | 28195424 | -0.82 | -1.21 | -0.44 | 0.56 | 0.45 | 0.71 |
| Ulcer haemorrhage | 73 | 270739 | 5878 | 28203273 | 0.36 | -0.02 | 0.75 | 1.29 | 1.02 | 1.63 |
| Arrhythmia | 70 | 270742 | 17048 | 28192103 | -1.21 | -1.61 | -0.81 | 0.43 | 0.34 | 0.54 |
| Subarachnoid haemorrhage | 69 | 270743 | 10104 | 28199047 | -0.48 | -0.89 | -0.08 | 0.71 | 0.56 | 0.90 |
| Haemorrhoids | 68 | 270744 | 8751 | 28200400 | -0.30 | -0.70 | 0.10 | 0.81 | 0.64 | 1.03 |
| Systemic inflammatory response syndrome | 66 | 270746 | 5191 | 28203960 | 0.40 | -0.01 | 0.81 | 1.32 | 1.04 | 1.68 |
| Vasculitis | 64 | 270748 | 7297 | 28201854 | -0.13 | -0.54 | 0.29 | 0.91 | 0.71 | 1.17 |
| Ventricular fibrillation | 63 | 270749 | 8377 | 28200774 | -0.35 | -0.77 | 0.07 | 0.78 | 0.61 | 1.01 |
| Hypertensive crisis | 61 | 270751 | 8021 | 28201130 | -0.33 | -0.76 | 0.10 | 0.79 | 0.62 | 1.02 |
| Angina pectoris | 60 | 270752 | 12410 | 28196741 | -0.98 | -1.41 | -0.55 | 0.51 | 0.39 | 0.65 |
| Coronary artery occlusion | 60 | 270752 | 5981 | 28203170 | 0.06 | -0.37 | 0.49 | 1.04 | 0.81 | 1.35 |
| Shock | 60 | 270752 | 10723 | 28198428 | -0.77 | -1.20 | -0.34 | 0.58 | 0.45 | 0.75 |
| Osteonecrosis | 54 | 270758 | 9590 | 28199561 | -0.76 | -1.21 | -0.30 | 0.59 | 0.45 | 0.77 |
| Shock haemorrhagic | 54 | 270758 | 9124 | 28200027 | -0.69 | -1.14 | -0.23 | 0.62 | 0.47 | 0.81 |
| Skin haemorrhage | 54 | 270758 | 6297 | 28202854 | -0.16 | -0.61 | 0.29 | 0.89 | 0.68 | 1.17 |
| Anaphylactic shock | 53 | 270759 | 12998 | 28196153 | -1.22 | -1.68 | -0.76 | 0.43 | 0.33 | 0.56 |
| Petechiae | 53 | 270759 | 4846 | 28204305 | 0.18 | -0.27 | 0.64 | 1.14 | 0.87 | 1.49 |
| Right ventricular failure | 53 | 270759 | 7373 | 28201778 | -0.41 | -0.87 | 0.05 | 0.75 | 0.57 | 0.98 |
| Post procedural haemorrhage | 52 | 270760 | 7308 | 28201843 | -0.43 | -0.89 | 0.04 | 0.74 | 0.57 | 0.98 |
| Hypertensive nephropathy | 51 | 270761 | 359 | 28208792 | 3.55 | 3.08 | 4.02 | 13.05 | 9.73 | 17.50 |
| Fluid overload | 50 | 270762 | 10285 | 28198866 | -0.97 | -1.44 | -0.50 | 0.51 | 0.39 | 0.67 |
| Sinus tachycardia | 49 | 270763 | 6639 | 28202512 | -0.37 | -0.85 | 0.10 | 0.77 | 0.58 | 1.02 |
| Peripheral coldness | 48 | 270764 | 6026 | 28203125 | -0.26 | -0.75 | 0.22 | 0.83 | 0.63 | 1.10 |
| Pulmonary congestion | 48 | 270764 | 6199 | 28202952 | -0.30 | -0.79 | 0.18 | 0.81 | 0.61 | 1.07 |
| Gingival bleeding | 47 | 270765 | 5335 | 28203816 | -0.12 | -0.61 | 0.37 | 0.92 | 0.69 | 1.22 |
| Pericarditis | 45 | 270767 | 6133 | 28203018 | -0.38 | -0.88 | 0.12 | 0.77 | 0.57 | 1.03 |
| Gastric haemorrhage | 43 | 270769 | 5983 | 28203168 | -0.41 | -0.92 | 0.10 | 0.75 | 0.56 | 1.01 |
| Ventricular extrasystoles | 43 | 270769 | 6290 | 28202861 | -0.48 | -0.99 | 0.03 | 0.71 | 0.53 | 0.96 |
| Subdural haemorrhage | 41 | 270771 | 3368 | 28205783 | 0.33 | -0.19 | 0.86 | 1.26 | 0.93 | 1.72 |
| Ascites | 39 | 270773 | 10728 | 28198423 | -1.38 | -1.92 | -0.84 | 0.38 | 0.28 | 0.52 |
| Haemorrhoidal haemorrhage | 39 | 270773 | 4553 | 28204598 | -0.16 | -0.70 | 0.38 | 0.89 | 0.65 | 1.22 |
| Increased tendency to bruise | 39 | 270773 | 4611 | 28204540 | -0.18 | -0.72 | 0.36 | 0.88 | 0.64 | 1.21 |
| Renal hypertension | 38 | 270774 | 337 | 28208814 | 3.24 | 2.70 | 3.79 | 10.31 | 7.37 | 14.42 |
| Vein disorder | 38 | 270774 | 3328 | 28205823 | 0.24 | -0.30 | 0.79 | 1.19 | 0.86 | 1.63 |
| Long qt syndrome | 37 | 270775 | 1953 | 28207198 | 0.95 | 0.40 | 1.50 | 1.95 | 1.41 | 2.70 |
| Peripheral vascular disorder | 36 | 270776 | 2768 | 28206383 | 0.43 | -0.13 | 0.99 | 1.35 | 0.97 | 1.87 |
| Torsade de pointes | 36 | 270776 | 5428 | 28203723 | -0.52 | -1.08 | 0.04 | 0.69 | 0.50 | 0.96 |
| Cardiac failure acute | 35 | 270777 | 4204 | 28204947 | -0.20 | -0.77 | 0.37 | 0.87 | 0.62 | 1.21 |
| Hypersensitivity vasculitis | 35 | 270777 | 3643 | 28205508 | 0.00 | -0.57 | 0.57 | 1.00 | 0.72 | 1.40 |
| Cardiovascular disorder | 34 | 270778 | 6141 | 28203010 | -0.78 | -1.36 | -0.20 | 0.58 | 0.41 | 0.81 |
| Gastric ulcer haemorrhage | 34 | 270778 | 4866 | 28204285 | -0.45 | -1.02 | 0.13 | 0.73 | 0.52 | 1.02 |
| Pulmonary haemorrhage | 33 | 270779 | 5643 | 28203508 | -0.70 | -1.29 | -0.12 | 0.61 | 0.44 | 0.86 |
| Purpura | 33 | 270779 | 4349 | 28204802 | -0.33 | -0.92 | 0.25 | 0.79 | 0.56 | 1.12 |
| Sinus bradycardia | 33 | 270779 | 6199 | 28202952 | -0.84 | -1.42 | -0.25 | 0.56 | 0.40 | 0.79 |
| Eye haemorrhage | 32 | 270780 | 6028 | 28203123 | -0.84 | -1.43 | -0.24 | 0.56 | 0.39 | 0.79 |
| Mouth haemorrhage | 32 | 270780 | 3846 | 28205305 | -0.20 | -0.80 | 0.39 | 0.87 | 0.61 | 1.23 |
| Myocarditis | 32 | 270780 | 5741 | 28203410 | -0.77 | -1.36 | -0.17 | 0.58 | 0.41 | 0.83 |
| Thrombophlebitis superficial | 31 | 270781 | 3315 | 28205836 | -0.04 | -0.64 | 0.57 | 0.97 | 0.68 | 1.39 |
| Vascular stent thrombosis | 31 | 270781 | 3581 | 28205570 | -0.15 | -0.75 | 0.46 | 0.90 | 0.63 | 1.29 |
| Renal haemorrhage | 29 | 270783 | 2533 | 28206618 | 0.25 | -0.38 | 0.87 | 1.19 | 0.82 | 1.71 |
| Varicose vein | 29 | 270783 | 3354 | 28205797 | -0.15 | -0.77 | 0.48 | 0.90 | 0.63 | 1.30 |
| Cerebral infarction | 28 | 270784 | 16139 | 28193012 | -2.44 | -3.07 | -1.80 | 0.18 | 0.13 | 0.27 |
| Embolism | 28 | 270784 | 5651 | 28203500 | -0.94 | -1.57 | -0.30 | 0.52 | 0.36 | 0.75 |
| Left ventricular dysfunction | 28 | 270784 | 4475 | 28204676 | -0.60 | -1.24 | 0.03 | 0.66 | 0.45 | 0.95 |
| Myocardial ischaemia | 28 | 270784 | 5811 | 28203340 | -0.98 | -1.61 | -0.34 | 0.51 | 0.35 | 0.73 |
| Stress cardiomyopathy | 28 | 270784 | 4851 | 28204300 | -0.72 | -1.36 | -0.08 | 0.61 | 0.42 | 0.88 |
| Colitis ischaemic | 27 | 270785 | 3815 | 28205336 | -0.43 | -1.08 | 0.22 | 0.74 | 0.51 | 1.08 |
| Intestinal haemorrhage | 27 | 270785 | 3014 | 28206137 | -0.10 | -0.75 | 0.55 | 0.93 | 0.64 | 1.36 |
| Urinary bladder haemorrhage | 27 | 270785 | 2348 | 28206803 | 0.25 | -0.40 | 0.90 | 1.19 | 0.82 | 1.74 |
| Circulatory collapse | 26 | 270786 | 6434 | 28202717 | -1.22 | -1.89 | -0.56 | 0.43 | 0.29 | 0.63 |
| Pericardial haemorrhage | 26 | 270786 | 3147 | 28206004 | -0.21 | -0.87 | 0.45 | 0.86 | 0.59 | 1.27 |
| Raynaud’s phenomenon | 26 | 270786 | 2940 | 28206211 | -0.12 | -0.78 | 0.55 | 0.92 | 0.63 | 1.36 |
| Venous thrombosis | 26 | 270786 | 3542 | 28205609 | -0.38 | -1.04 | 0.28 | 0.77 | 0.52 | 1.13 |
| Poor venous access | 24 | 270788 | 3181 | 28205970 | -0.34 | -1.03 | 0.35 | 0.79 | 0.53 | 1.18 |
| Wound haemorrhage | 24 | 270788 | 3856 | 28205295 | -0.61 | -1.30 | 0.08 | 0.65 | 0.44 | 0.98 |
| Acute coronary syndrome | 23 | 270789 | 4861 | 28204290 | -1.00 | -1.70 | -0.29 | 0.50 | 0.33 | 0.75 |
| Extrasystoles | 23 | 270789 | 2594 | 28206557 | -0.11 | -0.82 | 0.59 | 0.93 | 0.61 | 1.39 |
| Infarction | 23 | 270789 | 3356 | 28205795 | -0.47 | -1.18 | 0.23 | 0.72 | 0.48 | 1.08 |
| Renal artery stenosis | 23 | 270789 | 665 | 28208486 | 1.74 | 1.03 | 2.44 | 3.41 | 2.25 | 5.17 |
| Renal infarct | 23 | 270789 | 717 | 28208434 | 1.64 | 0.93 | 2.35 | 3.18 | 2.10 | 4.82 |
| Sudden cardiac death | 23 | 270789 | 2798 | 28206353 | -0.22 | -0.92 | 0.49 | 0.86 | 0.57 | 1.29 |
| Thrombotic microangiopathy | 23 | 270789 | 7784 | 28201367 | -1.67 | -2.38 | -0.96 | 0.31 | 0.21 | 0.47 |
| Hypovolaemic shock | 22 | 270790 | 2705 | 28206446 | -0.23 | -0.96 | 0.49 | 0.85 | 0.56 | 1.29 |
| Thrombophlebitis | 22 | 270790 | 2931 | 28206220 | -0.35 | -1.07 | 0.38 | 0.79 | 0.52 | 1.20 |
| Thrombosis in device | 22 | 270790 | 2164 | 28206987 | 0.08 | -0.64 | 0.80 | 1.06 | 0.69 | 1.61 |
| Varices oesophageal | 22 | 270790 | 2022 | 28207129 | 0.17 | -0.55 | 0.90 | 1.13 | 0.74 | 1.72 |
| Peripheral ischaemia | 21 | 270791 | 3039 | 28206112 | -0.46 | -1.20 | 0.28 | 0.72 | 0.47 | 1.11 |
| Sinus node dysfunction | 21 | 270791 | 2840 | 28206311 | -0.37 | -1.11 | 0.38 | 0.77 | 0.50 | 1.19 |
| Small intestinal haemorrhage | 21 | 270791 | 2726 | 28206425 | -0.31 | -1.05 | 0.43 | 0.81 | 0.52 | 1.24 |
| Ventricular arrhythmia | 21 | 270791 | 2597 | 28206554 | -0.24 | -0.98 | 0.50 | 0.85 | 0.55 | 1.30 |
| Cardiac flutter | 20 | 270792 | 2544 | 28206607 | -0.28 | -1.04 | 0.48 | 0.82 | 0.53 | 1.28 |
| Diarrhoea haemorrhagic | 20 | 270792 | 3891 | 28205260 | -0.88 | -1.64 | -0.12 | 0.54 | 0.35 | 0.84 |
| Intestinal ischaemia | 20 | 270792 | 2874 | 28206277 | -0.45 | -1.21 | 0.31 | 0.73 | 0.47 | 1.13 |
| Pre-eclampsia | 20 | 270792 | 2315 | 28206836 | -0.15 | -0.91 | 0.61 | 0.90 | 0.58 | 1.40 |
| Procedural haemorrhage | 20 | 270792 | 2595 | 28206556 | -0.31 | -1.07 | 0.45 | 0.81 | 0.52 | 1.25 |
| Pulmonary alveolar haemorrhage | 20 | 270792 | 3962 | 28205189 | -0.90 | -1.66 | -0.14 | 0.53 | 0.34 | 0.83 |
| Venous thrombosis limb | 20 | 270792 | 2748 | 28206403 | -0.39 | -1.15 | 0.37 | 0.76 | 0.49 | 1.18 |
| Arterial occlusive disease | 19 | 270793 | 3105 | 28206046 | -0.63 | -1.41 | 0.15 | 0.64 | 0.41 | 1.01 |
| Atrial flutter | 19 | 270793 | 4728 | 28204423 | -1.23 | -2.01 | -0.45 | 0.42 | 0.27 | 0.67 |
| Cyanosis | 19 | 270793 | 4119 | 28205032 | -1.03 | -1.81 | -0.25 | 0.49 | 0.31 | 0.76 |
| Haemorrhagic arteriovenous malformation | 19 | 270793 | 2817 | 28206334 | -0.49 | -1.28 | 0.29 | 0.71 | 0.45 | 1.11 |
| Ischaemic cardiomyopathy | 19 | 270793 | 1482 | 28207669 | 0.40 | -0.38 | 1.18 | 1.32 | 0.84 | 2.08 |
| Portal vein thrombosis | 19 | 270793 | 1982 | 28207169 | 0.00 | -0.78 | 0.78 | 1.00 | 0.64 | 1.57 |
| Pulseless electrical activity | 19 | 270793 | 2371 | 28206780 | -0.25 | -1.03 | 0.53 | 0.84 | 0.53 | 1.32 |
| Temporal arteritis | 19 | 270793 | 1260 | 28207891 | 0.62 | -0.16 | 1.40 | 1.55 | 0.98 | 2.44 |
| Vascular occlusion | 19 | 270793 | 1748 | 28207403 | 0.17 | -0.61 | 0.95 | 1.13 | 0.72 | 1.77 |
| Cardiomyopathy | 18 | 270794 | 6237 | 28202914 | -1.70 | -2.50 | -0.89 | 0.31 | 0.19 | 0.49 |
| Duodenal ulcer haemorrhage | 18 | 270794 | 2406 | 28206745 | -0.35 | -1.15 | 0.46 | 0.78 | 0.49 | 1.25 |
| Lymphoedema | 18 | 270794 | 3139 | 28206012 | -0.72 | -1.53 | 0.08 | 0.60 | 0.38 | 0.96 |
| Phlebitis | 18 | 270794 | 2819 | 28206332 | -0.57 | -1.37 | 0.23 | 0.67 | 0.42 | 1.07 |
| Retinal haemorrhage | 18 | 270794 | 4012 | 28205139 | -1.07 | -1.87 | -0.27 | 0.47 | 0.30 | 0.75 |
| Retinal vein occlusion | 18 | 270794 | 1888 | 28207263 | -0.01 | -0.81 | 0.79 | 0.99 | 0.62 | 1.58 |
| Thrombotic thrombocytopenic purpura | 18 | 270794 | 2926 | 28206225 | -0.62 | -1.43 | 0.18 | 0.65 | 0.41 | 1.03 |
| Vasodilatation | 18 | 270794 | 1969 | 28207182 | -0.07 | -0.87 | 0.74 | 0.95 | 0.60 | 1.52 |
| Venous occlusion | 18 | 270794 | 1379 | 28207772 | 0.42 | -0.38 | 1.23 | 1.35 | 0.85 | 2.14 |
| Ventricular septal defect | 18 | 270794 | 4598 | 28204553 | -1.26 | -2.07 | -0.46 | 0.41 | 0.26 | 0.66 |
| Angina unstable | 17 | 270795 | 4053 | 28205098 | -1.16 | -1.99 | -0.33 | 0.44 | 0.28 | 0.71 |
| Cardiogenic shock | 17 | 270795 | 4828 | 28204323 | -1.41 | -2.24 | -0.58 | 0.37 | 0.23 | 0.60 |
| Cardiomegaly | 17 | 270795 | 3287 | 28205864 | -0.87 | -1.70 | -0.04 | 0.55 | 0.34 | 0.88 |
| Hypoxic-ischaemic encephalopathy | 17 | 270795 | 1213 | 28207938 | 0.52 | -0.31 | 1.35 | 1.44 | 0.89 | 2.33 |
| Intracranial aneurysm | 17 | 270795 | 2067 | 28207084 | -0.22 | -1.04 | 0.61 | 0.86 | 0.53 | 1.39 |
| Retroperitoneal haemorrhage | 17 | 270795 | 1940 | 28207211 | -0.13 | -0.96 | 0.70 | 0.92 | 0.57 | 1.48 |
| Splenic infarction | 17 | 270795 | 1143 | 28208008 | 0.60 | -0.23 | 1.43 | 1.53 | 0.94 | 2.46 |
| Ecchymosis | 16 | 270796 | 2429 | 28206722 | -0.53 | -1.38 | 0.33 | 0.69 | 0.42 | 1.13 |
| Haemorrhagic diathesis | 16 | 270796 | 1791 | 28207360 | -0.10 | -0.96 | 0.76 | 0.93 | 0.57 | 1.53 |
| Oesophageal varices haemorrhage | 16 | 270796 | 2149 | 28207002 | -0.35 | -1.21 | 0.50 | 0.78 | 0.48 | 1.28 |
| Dizziness postural | 15 | 270797 | 3464 | 28205687 | -1.12 | -2.00 | -0.23 | 0.46 | 0.28 | 0.76 |
| Haemorrhagic transformation stroke | 15 | 270797 | 1713 | 28207438 | -0.13 | -1.01 | 0.76 | 0.91 | 0.55 | 1.52 |
| Henoch-schonlein purpura | 15 | 270797 | 895 | 28208256 | 0.76 | -0.13 | 1.65 | 1.70 | 1.02 | 2.84 |
| Large intestinal haemorrhage | 15 | 270797 | 1969 | 28207182 | -0.32 | -1.21 | 0.57 | 0.80 | 0.48 | 1.33 |
| Migraine with aura | 15 | 270797 | 1315 | 28207836 | 0.24 | -0.65 | 1.12 | 1.18 | 0.71 | 1.96 |
| Papilloedema | 15 | 270797 | 2136 | 28207015 | -0.43 | -1.32 | 0.45 | 0.74 | 0.44 | 1.23 |
| Uterine haemorrhage | 15 | 270797 | 3014 | 28206137 | -0.92 | -1.81 | -0.03 | 0.53 | 0.32 | 0.87 |
| Blood pressure fluctuation | 14 | 270798 | 8363 | 28200788 | -2.47 | -3.39 | -1.55 | 0.18 | 0.11 | 0.30 |
| Disseminated intravascular coagulation | 14 | 270798 | 5064 | 28204087 | -1.75 | -2.67 | -0.83 | 0.30 | 0.17 | 0.50 |
| Haemorrhage urinary tract | 14 | 270798 | 1679 | 28207472 | -0.20 | -1.12 | 0.73 | 0.87 | 0.52 | 1.48 |
| Kounis syndrome | 14 | 270798 | 1545 | 28207606 | -0.08 | -1.00 | 0.84 | 0.95 | 0.56 | 1.60 |
| Left ventricular failure | 14 | 270798 | 2417 | 28206734 | -0.70 | -1.62 | 0.22 | 0.61 | 0.36 | 1.03 |
| Mitral valve incompetence | 14 | 270798 | 3864 | 28205287 | -1.37 | -2.29 | -0.45 | 0.39 | 0.23 | 0.65 |
| Muscle haemorrhage | 14 | 270798 | 3084 | 28206067 | -1.05 | -1.97 | -0.13 | 0.48 | 0.28 | 0.81 |
| Tachyarrhythmia | 14 | 270798 | 2348 | 28206803 | -0.66 | -1.58 | 0.26 | 0.63 | 0.37 | 1.06 |
| Vasculitic rash | 14 | 270798 | 857 | 28208294 | 0.72 | -0.20 | 1.64 | 1.66 | 0.98 | 2.82 |
| Ventricular dysfunction | 14 | 270798 | 924 | 28208227 | 0.62 | -0.30 | 1.54 | 1.55 | 0.91 | 2.62 |
| Ventricular hypokinesia | 14 | 270798 | 1924 | 28207227 | -0.38 | -1.30 | 0.54 | 0.76 | 0.45 | 1.29 |
| Congestive cardiomyopathy | 13 | 270799 | 2619 | 28206532 | -0.92 | -1.88 | 0.04 | 0.53 | 0.31 | 0.91 |
| Diabetic foot | 13 | 270799 | 1842 | 28207309 | -0.43 | -1.38 | 0.53 | 0.74 | 0.43 | 1.28 |
| Diverticulum intestinal haemorrhagic | 13 | 270799 | 3239 | 28205912 | -1.22 | -2.18 | -0.26 | 0.43 | 0.25 | 0.74 |
| Intraventricular haemorrhage | 13 | 270799 | 2181 | 28206970 | -0.66 | -1.62 | 0.30 | 0.63 | 0.37 | 1.09 |
| Necrosis | 13 | 270799 | 2062 | 28207089 | -0.58 | -1.54 | 0.37 | 0.67 | 0.39 | 1.15 |
| Pharyngeal haemorrhage | 13 | 270799 | 806 | 28208345 | 0.70 | -0.25 | 1.66 | 1.64 | 0.95 | 2.83 |
| Poor peripheral circulation | 13 | 270799 | 2262 | 28206889 | -0.71 | -1.67 | 0.24 | 0.61 | 0.35 | 1.05 |
| Pyogenic granuloma | 13 | 270799 | 311 | 28208840 | 1.91 | 0.96 | 2.87 | 3.87 | 2.22 | 6.75 |
| Tricuspid valve incompetence | 13 | 270799 | 3022 | 28206129 | -1.12 | -2.08 | -0.16 | 0.46 | 0.27 | 0.79 |
| Aortic aneurysm | 12 | 270800 | 3161 | 28205990 | -1.29 | -2.30 | -0.29 | 0.41 | 0.23 | 0.71 |
| Gangrene | 12 | 270800 | 2759 | 28206392 | -1.10 | -2.10 | -0.10 | 0.46 | 0.26 | 0.82 |
| Haemarthrosis | 12 | 270800 | 3814 | 28205337 | -1.56 | -2.56 | -0.56 | 0.34 | 0.19 | 0.59 |
| Jugular vein thrombosis | 12 | 270800 | 831 | 28208320 | 0.55 | -0.45 | 1.55 | 1.47 | 0.83 | 2.61 |
| Orthopnoea | 12 | 270800 | 1444 | 28207707 | -0.20 | -1.20 | 0.80 | 0.87 | 0.49 | 1.54 |
| Peripheral arterial occlusive disease | 12 | 270800 | 3224 | 28205927 | -1.32 | -2.32 | -0.32 | 0.40 | 0.23 | 0.70 |
| Retinal artery occlusion | 12 | 270800 | 1447 | 28207704 | -0.20 | -1.20 | 0.80 | 0.87 | 0.49 | 1.53 |
| Tumour haemorrhage | 12 | 270800 | 2583 | 28206568 | -1.01 | -2.01 | -0.01 | 0.49 | 0.28 | 0.87 |
| Vascular purpura | 12 | 270800 | 1585 | 28207566 | -0.33 | -1.33 | 0.67 | 0.80 | 0.45 | 1.40 |
| Vascular rupture | 12 | 270800 | 1043 | 28208108 | 0.25 | -0.75 | 1.25 | 1.19 | 0.67 | 2.10 |
| Venoocclusive disease | 12 | 270800 | 2769 | 28206382 | -1.11 | -2.11 | -0.11 | 0.46 | 0.26 | 0.81 |
| Anaphylactoid reaction | 11 | 270801 | 1558 | 28207593 | -0.42 | -1.47 | 0.63 | 0.74 | 0.41 | 1.35 |
| Essential hypertension | 11 | 270801 | 879 | 28208272 | 0.36 | -0.69 | 1.41 | 1.29 | 0.71 | 2.33 |
| Gastrointestinal polyp haemorrhage | 11 | 270801 | 755 | 28208396 | 0.56 | -0.49 | 1.61 | 1.48 | 0.82 | 2.69 |
| Intra-abdominal haemorrhage | 11 | 270801 | 1461 | 28207690 | -0.33 | -1.38 | 0.72 | 0.79 | 0.44 | 1.43 |
| Intracardiac thrombus | 11 | 270801 | 1690 | 28207461 | -0.54 | -1.59 | 0.51 | 0.69 | 0.38 | 1.24 |
| Peripheral artery occlusion | 11 | 270801 | 1326 | 28207825 | -0.20 | -1.25 | 0.85 | 0.87 | 0.48 | 1.57 |
| Stoma site haemorrhage | 11 | 270801 | 1387 | 28207764 | -0.26 | -1.31 | 0.79 | 0.83 | 0.46 | 1.51 |
| Superior vena cava syndrome | 11 | 270801 | 514 | 28208637 | 1.07 | 0.02 | 2.12 | 2.12 | 1.16 | 3.85 |
| Supraventricular extrasystoles | 11 | 270801 | 1651 | 28207500 | -0.50 | -1.55 | 0.55 | 0.70 | 0.39 | 1.27 |
| Abdominal wall haematoma | 10 | 270802 | 1542 | 28207609 | -0.54 | -1.65 | 0.57 | 0.69 | 0.37 | 1.28 |
| Arteriospasm coronary | 10 | 270802 | 1635 | 28207516 | -0.62 | -1.73 | 0.49 | 0.65 | 0.35 | 1.21 |
| Atrioventricular block | 10 | 270802 | 2960 | 28206191 | -1.45 | -2.56 | -0.35 | 0.36 | 0.20 | 0.68 |
| Atrioventricular block complete | 10 | 270802 | 3184 | 28205967 | -1.56 | -2.66 | -0.45 | 0.34 | 0.18 | 0.63 |
| Cerebral ischaemia | 10 | 270802 | 2676 | 28206475 | -1.31 | -2.42 | -0.20 | 0.40 | 0.22 | 0.75 |
| Embolic stroke | 10 | 270802 | 3370 | 28205781 | -1.64 | -2.74 | -0.53 | 0.32 | 0.17 | 0.59 |
| Eosinophilic granulomatosis with polyangiitis | 10 | 270802 | 794 | 28208357 | 0.37 | -0.74 | 1.47 | 1.29 | 0.69 | 2.41 |
| Gastritis haemorrhagic | 10 | 270802 | 1467 | 28207684 | -0.47 | -1.58 | 0.64 | 0.72 | 0.39 | 1.34 |
| Gastrointestinal ulcer haemorrhage | 10 | 270802 | 903 | 28208248 | 0.19 | -0.91 | 1.30 | 1.15 | 0.61 | 2.14 |
| Haemothorax | 10 | 270802 | 2347 | 28206804 | -1.13 | -2.23 | -0.02 | 0.46 | 0.24 | 0.85 |
| Ischaemia | 10 | 270802 | 1903 | 28207248 | -0.83 | -1.94 | 0.27 | 0.56 | 0.30 | 1.04 |
| Oesophageal haemorrhage | 10 | 270802 | 893 | 28208258 | 0.21 | -0.90 | 1.32 | 1.16 | 0.62 | 2.16 |
| Peptic ulcer haemorrhage | 10 | 270802 | 738 | 28208413 | 0.46 | -0.64 | 1.57 | 1.38 | 0.74 | 2.58 |
| Retroperitoneal haematoma | 10 | 270802 | 1890 | 28207261 | -0.82 | -1.93 | 0.28 | 0.56 | 0.30 | 1.05 |
| Subcutaneous haematoma | 10 | 270802 | 1583 | 28207568 | -0.58 | -1.68 | 0.53 | 0.67 | 0.36 | 1.25 |
| Telangiectasia | 10 | 270802 | 497 | 28208654 | 0.98 | -0.13 | 2.09 | 1.99 | 1.07 | 3.73 |
| Vascular dementia | 10 | 270802 | 897 | 28208254 | 0.20 | -0.90 | 1.31 | 1.15 | 0.62 | 2.15 |
| Vascular pseudoaneurysm | 10 | 270802 | 2023 | 28207128 | -0.92 | -2.02 | 0.19 | 0.53 | 0.28 | 0.98 |
| Atrial thrombosis | 9 | 270803 | 1387 | 28207764 | -0.54 | -1.71 | 0.64 | 0.69 | 0.36 | 1.32 |
| Cardiac tamponade | 9 | 270803 | 2727 | 28206424 | -1.48 | -2.65 | -0.31 | 0.36 | 0.19 | 0.69 |
| Injection site haematoma | 9 | 270803 | 1424 | 28207727 | -0.57 | -1.75 | 0.60 | 0.67 | 0.35 | 1.29 |
| Lymphangitis | 9 | 270803 | 414 | 28208737 | 1.07 | -0.10 | 2.24 | 2.12 | 1.10 | 4.11 |
| Postural orthostatic tachycardia syndrome | 9 | 270803 | 904 | 28208247 | 0.05 | -1.12 | 1.22 | 1.04 | 0.54 | 2.00 |
| Prerenal failure | 9 | 270803 | 1443 | 28207708 | -0.59 | -1.76 | 0.58 | 0.66 | 0.34 | 1.27 |
| Prinzmetal angina | 9 | 270803 | 1075 | 28208076 | -0.19 | -1.36 | 0.99 | 0.88 | 0.46 | 1.69 |
| Renal cortical necrosis | 9 | 270803 | 104 | 28209047 | 2.59 | 1.42 | 3.77 | 6.34 | 3.21 | 12.53 |
| Spinal epidural haematoma | 9 | 270803 | 592 | 28208559 | 0.61 | -0.56 | 1.79 | 1.54 | 0.80 | 2.97 |
| Tongue haemorrhage | 9 | 270803 | 709 | 28208442 | 0.37 | -0.80 | 1.55 | 1.30 | 0.67 | 2.51 |
| Vascular compression | 9 | 270803 | 237 | 28208914 | 1.74 | 0.57 | 2.92 | 3.42 | 1.76 | 6.66 |
| Vascular pain | 9 | 270803 | 729 | 28208422 | 0.34 | -0.84 | 1.51 | 1.27 | 0.66 | 2.44 |
| Vascular stent occlusion | 9 | 270803 | 761 | 28208390 | 0.28 | -0.89 | 1.45 | 1.22 | 0.63 | 2.35 |
| White matter lesion | 9 | 270803 | 896 | 28208255 | 0.06 | -1.11 | 1.24 | 1.04 | 0.54 | 2.01 |
| Anal haemorrhage | 8 | 270804 | 1044 | 28208107 | -0.31 | -1.56 | 0.95 | 0.81 | 0.40 | 1.62 |
| Aneurysm | 8 | 270804 | 2061 | 28207090 | -1.25 | -2.50 | 0.01 | 0.42 | 0.21 | 0.84 |
| Application site haemorrhage | 8 | 270804 | 1732 | 28207419 | -1.00 | -2.26 | 0.25 | 0.50 | 0.25 | 0.99 |
| Arteriosclerosis | 8 | 270804 | 2667 | 28206484 | -1.61 | -2.86 | -0.35 | 0.33 | 0.16 | 0.65 |
| Cardiac valve disease | 8 | 270804 | 2146 | 28207005 | -1.30 | -2.56 | -0.05 | 0.40 | 0.20 | 0.81 |
| Cerebellar haemorrhage | 8 | 270804 | 1400 | 28207751 | -0.71 | -1.96 | 0.55 | 0.61 | 0.30 | 1.22 |
| Coronary artery stenosis | 8 | 270804 | 2046 | 28207105 | -1.24 | -2.49 | 0.02 | 0.42 | 0.21 | 0.84 |
| Endocarditis | 8 | 270804 | 2247 | 28206904 | -1.37 | -2.62 | -0.11 | 0.39 | 0.19 | 0.77 |
| Gastrointestinal necrosis | 8 | 270804 | 1322 | 28207829 | -0.63 | -1.88 | 0.63 | 0.64 | 0.32 | 1.29 |
| Hyperaemia | 8 | 270804 | 608 | 28208543 | 0.42 | -0.84 | 1.67 | 1.34 | 0.67 | 2.69 |
| Ischaemic cerebral infarction | 8 | 270804 | 1048 | 28208103 | -0.31 | -1.57 | 0.94 | 0.80 | 0.40 | 1.61 |
| Localised oedema | 8 | 270804 | 1826 | 28207325 | -1.08 | -2.33 | 0.18 | 0.47 | 0.24 | 0.94 |
| Myocardial oedema | 8 | 270804 | 170 | 28208981 | 1.95 | 0.70 | 3.21 | 3.99 | 1.96 | 8.10 |
| Nocturnal dyspnoea | 8 | 270804 | 725 | 28208426 | 0.19 | -1.07 | 1.44 | 1.14 | 0.57 | 2.29 |
| Peripheral artery stenosis | 8 | 270804 | 629 | 28208522 | 0.37 | -0.88 | 1.63 | 1.30 | 0.65 | 2.61 |
| Peripheral artery thrombosis | 8 | 270804 | 1182 | 28207969 | -0.48 | -1.73 | 0.78 | 0.72 | 0.36 | 1.44 |
| Peripheral embolism | 8 | 270804 | 720 | 28208431 | 0.20 | -1.06 | 1.45 | 1.15 | 0.57 | 2.30 |
| Renal haematoma | 8 | 270804 | 674 | 28208477 | 0.28 | -0.97 | 1.54 | 1.22 | 0.61 | 2.45 |
| Subclavian vein thrombosis | 8 | 270804 | 898 | 28208253 | -0.10 | -1.36 | 1.15 | 0.93 | 0.46 | 1.87 |
| Traumatic haematoma | 8 | 270804 | 1168 | 28207983 | -0.46 | -1.71 | 0.80 | 0.73 | 0.36 | 1.45 |
| Traumatic haemorrhage | 8 | 270804 | 1200 | 28207951 | -0.50 | -1.75 | 0.76 | 0.71 | 0.35 | 1.42 |
| Acute pulmonary oedema | 7 | 270805 | 2316 | 28206835 | -1.59 | -2.95 | -0.24 | 0.33 | 0.16 | 0.69 |
| Angiopathy | 7 | 270805 | 1723 | 28207428 | -1.18 | -2.53 | 0.18 | 0.44 | 0.21 | 0.92 |
| Arteriosclerosis coronary artery | 7 | 270805 | 1762 | 28207389 | -1.21 | -2.56 | 0.15 | 0.43 | 0.20 | 0.90 |
| Atrioventricular block second degree | 7 | 270805 | 1446 | 28207705 | -0.93 | -2.29 | 0.42 | 0.52 | 0.25 | 1.10 |
| Conduction disorder | 7 | 270805 | 705 | 28208446 | 0.04 | -1.31 | 1.40 | 1.03 | 0.49 | 2.17 |
| Enterocolitis haemorrhagic | 7 | 270805 | 968 | 28208183 | -0.38 | -1.74 | 0.97 | 0.77 | 0.36 | 1.61 |
| Gastric antral vascular ectasia | 7 | 270805 | 269 | 28208882 | 1.26 | -0.09 | 2.62 | 2.43 | 1.15 | 5.15 |
| Heart valve incompetence | 7 | 270805 | 1304 | 28207847 | -0.79 | -2.15 | 0.57 | 0.58 | 0.27 | 1.21 |
| Hypertensive heart disease | 7 | 270805 | 903 | 28208248 | -0.29 | -1.64 | 1.07 | 0.82 | 0.39 | 1.72 |
| Lip haemorrhage | 7 | 270805 | 427 | 28208724 | 0.70 | -0.66 | 2.05 | 1.63 | 0.77 | 3.44 |
| Lymphangioleiomyomatosis | 7 | 270805 | 98 | 28209053 | 2.32 | 0.97 | 3.68 | 5.21 | 2.42 | 11.21 |
| Mesenteric artery thrombosis | 7 | 270805 | 301 | 28208850 | 1.13 | -0.23 | 2.48 | 2.21 | 1.05 | 4.68 |
| Multiple system atrophy | 7 | 270805 | 393 | 28208758 | 0.80 | -0.55 | 2.16 | 1.76 | 0.83 | 3.71 |
| Periorbital haematoma | 7 | 270805 | 337 | 28208814 | 0.99 | -0.36 | 2.35 | 2.01 | 0.95 | 4.24 |
| Peripheral venous disease | 7 | 270805 | 1333 | 28207818 | -0.82 | -2.18 | 0.54 | 0.56 | 0.27 | 1.19 |
| Polyarteritis nodosa | 7 | 270805 | 279 | 28208872 | 1.22 | -0.14 | 2.58 | 2.36 | 1.11 | 5.00 |
| Portal hypertension | 7 | 270805 | 1388 | 28207763 | -0.88 | -2.23 | 0.48 | 0.54 | 0.26 | 1.14 |
| Pulmonary artery thrombosis | 7 | 270805 | 722 | 28208429 | 0.01 | -1.34 | 1.37 | 1.01 | 0.48 | 2.12 |
| Spontaneous haemorrhage | 7 | 270805 | 412 | 28208739 | 0.74 | -0.61 | 2.10 | 1.68 | 0.80 | 3.55 |
| Superior mesenteric artery syndrome | 7 | 270805 | 240 | 28208911 | 1.40 | 0.04 | 2.75 | 2.67 | 1.26 | 5.67 |
| Thrombosis mesenteric vessel | 7 | 270805 | 169 | 28208982 | 1.79 | 0.43 | 3.14 | 3.53 | 1.66 | 7.53 |
| Vascular device infection | 7 | 270805 | 1932 | 28207219 | -1.34 | -2.69 | 0.02 | 0.39 | 0.19 | 0.83 |
| Vitreous haemorrhage | 7 | 270805 | 2659 | 28206492 | -1.79 | -3.14 | -0.43 | 0.29 | 0.14 | 0.61 |
| Aortic aneurysm rupture | 6 | 270806 | 726 | 28208425 | -0.20 | -1.68 | 1.28 | 0.87 | 0.39 | 1.94 |
| Aortic stenosis | 6 | 270806 | 1398 | 28207753 | -1.09 | -2.57 | 0.39 | 0.47 | 0.21 | 1.04 |
| Aortic valve stenosis | 6 | 270806 | 698 | 28208453 | -0.15 | -1.63 | 1.34 | 0.90 | 0.40 | 2.02 |
| Blood pressure inadequately controlled | 6 | 270806 | 1858 | 28207293 | -1.49 | -2.97 | 0.00 | 0.35 | 0.16 | 0.79 |
| Bundle branch block right | 6 | 270806 | 1225 | 28207926 | -0.91 | -2.39 | 0.57 | 0.53 | 0.24 | 1.18 |
| Cardiac dysfunction | 6 | 270806 | 1044 | 28208107 | -0.69 | -2.17 | 0.79 | 0.62 | 0.28 | 1.38 |
| Cardiac fibrillation | 6 | 270806 | 536 | 28208615 | 0.20 | -1.28 | 1.68 | 1.15 | 0.51 | 2.57 |
| Cardiorenal syndrome | 6 | 270806 | 239 | 28208912 | 1.20 | -0.28 | 2.68 | 2.33 | 1.03 | 5.23 |
| Cerebellar infarction | 6 | 270806 | 961 | 28208190 | -0.58 | -2.06 | 0.91 | 0.67 | 0.30 | 1.49 |
| Cerebral thrombosis | 6 | 270806 | 1365 | 28207786 | -1.06 | -2.54 | 0.42 | 0.48 | 0.21 | 1.07 |
| Conjunctival haemorrhage | 6 | 270806 | 1773 | 28207378 | -1.42 | -2.91 | 0.06 | 0.37 | 0.17 | 0.83 |
| Cutaneous vasculitis | 6 | 270806 | 708 | 28208443 | -0.17 | -1.65 | 1.32 | 0.89 | 0.40 | 1.99 |
| Diastolic dysfunction | 6 | 270806 | 849 | 28208302 | -0.41 | -1.89 | 1.07 | 0.75 | 0.34 | 1.68 |
| Embolism venous | 6 | 270806 | 1530 | 28207621 | -1.22 | -2.70 | 0.27 | 0.43 | 0.19 | 0.95 |
| Extremity necrosis | 6 | 270806 | 841 | 28208310 | -0.40 | -1.88 | 1.09 | 0.76 | 0.34 | 1.69 |
| Genital haemorrhage | 6 | 270806 | 4564 | 28204587 | -2.76 | -4.24 | -1.27 | 0.15 | 0.07 | 0.33 |
| Haemorrhagic erosive gastritis | 6 | 270806 | 580 | 28208571 | 0.10 | -1.39 | 1.58 | 1.07 | 0.48 | 2.39 |
| Incision site haemorrhage | 6 | 270806 | 514 | 28208637 | 0.26 | -1.23 | 1.74 | 1.20 | 0.53 | 2.67 |
| Mesenteric vein thrombosis | 6 | 270806 | 488 | 28208663 | 0.32 | -1.16 | 1.81 | 1.25 | 0.56 | 2.80 |
| Peritoneal haemorrhage | 6 | 270806 | 1352 | 28207799 | -1.05 | -2.53 | 0.44 | 0.48 | 0.22 | 1.08 |
| Post procedural haematoma | 6 | 270806 | 1607 | 28207544 | -1.28 | -2.77 | 0.20 | 0.41 | 0.18 | 0.91 |
| Retinal oedema | 6 | 270806 | 699 | 28208452 | -0.15 | -1.63 | 1.33 | 0.90 | 0.40 | 2.01 |
| Retinopathy | 6 | 270806 | 1763 | 28207388 | -1.41 | -2.90 | 0.07 | 0.37 | 0.17 | 0.83 |
| Right ventricular dysfunction | 6 | 270806 | 544 | 28208607 | 0.18 | -1.30 | 1.67 | 1.14 | 0.51 | 2.54 |
| Single umbilical artery | 6 | 270806 | 159 | 28208992 | 1.65 | 0.17 | 3.13 | 3.21 | 1.42 | 7.25 |
| Sinus arrest | 6 | 270806 | 1076 | 28208075 | -0.73 | -2.21 | 0.75 | 0.60 | 0.27 | 1.34 |
| Systemic scleroderma | 6 | 270806 | 429 | 28208722 | 0.49 | -1.00 | 1.97 | 1.41 | 0.63 | 3.15 |
| Thalamic infarction | 6 | 270806 | 895 | 28208256 | -0.48 | -1.96 | 1.00 | 0.71 | 0.32 | 1.60 |
| Thrombotic stroke | 6 | 270806 | 607 | 28208544 | 0.04 | -1.44 | 1.52 | 1.03 | 0.46 | 2.30 |
| Vascular injury | 6 | 270806 | 974 | 28208177 | -0.60 | -2.08 | 0.89 | 0.66 | 0.30 | 1.47 |
| Vascular stent stenosis | 6 | 270806 | 706 | 28208445 | -0.16 | -1.64 | 1.32 | 0.89 | 0.40 | 1.99 |
| Vasoconstriction | 6 | 270806 | 451 | 28208700 | 0.42 | -1.06 | 1.91 | 1.35 | 0.60 | 3.01 |
| Ventricular failure | 6 | 270806 | 197 | 28208954 | 1.42 | -0.06 | 2.90 | 2.72 | 1.21 | 6.12 |
| Aortic dissection | 5 | 270807 | 1758 | 28207393 | -1.65 | -3.30 | 0.00 | 0.32 | 0.13 | 0.76 |
| Atrial septal defect | 5 | 270807 | 2481 | 28206670 | -2.13 | -3.79 | -0.48 | 0.23 | 0.09 | 0.54 |
| Atrioventricular block first degree | 5 | 270807 | 1611 | 28207540 | -1.53 | -3.18 | 0.13 | 0.34 | 0.14 | 0.83 |
| Cardiopulmonary failure | 5 | 270807 | 1653 | 28207498 | -1.56 | -3.22 | 0.09 | 0.34 | 0.14 | 0.81 |
| Carotid artery stenosis | 5 | 270807 | 1132 | 28208019 | -1.04 | -2.69 | 0.61 | 0.48 | 0.20 | 1.16 |
| Cerebral haematoma | 5 | 270807 | 2102 | 28207049 | -1.90 | -3.56 | -0.25 | 0.27 | 0.11 | 0.64 |
| Cerebrovascular disorder | 5 | 270807 | 1055 | 28208096 | -0.94 | -2.60 | 0.71 | 0.52 | 0.21 | 1.25 |
| Chronic pigmented purpura | 5 | 270807 | 156 | 28208995 | 1.44 | -0.22 | 3.09 | 2.75 | 1.13 | 6.71 |
| Ear haemorrhage | 5 | 270807 | 901 | 28208250 | -0.73 | -2.38 | 0.93 | 0.60 | 0.25 | 1.45 |
| Foetal placental thrombosis | 5 | 270807 | 34 | 28209117 | 2.66 | 1.00 | 4.31 | 6.66 | 2.60 | 17.02 |
| Heart valve stenosis | 5 | 270807 | 211 | 28208940 | 1.11 | -0.55 | 2.76 | 2.18 | 0.90 | 5.29 |
| Hypertensive cardiomyopathy | 5 | 270807 | 142 | 28209009 | 1.54 | -0.12 | 3.19 | 2.95 | 1.21 | 7.20 |
| Hypertensive emergency | 5 | 270807 | 784 | 28208367 | -0.54 | -2.20 | 1.11 | 0.69 | 0.28 | 1.65 |
| Intestinal infarction | 5 | 270807 | 374 | 28208777 | 0.42 | -1.23 | 2.08 | 1.34 | 0.56 | 3.25 |
| Intra-abdominal haematoma | 5 | 270807 | 971 | 28208180 | -0.83 | -2.49 | 0.82 | 0.56 | 0.23 | 1.35 |
| Intracranial venous sinus thrombosis | 5 | 270807 | 710 | 28208441 | -0.41 | -2.06 | 1.25 | 0.75 | 0.31 | 1.81 |
| Labile blood pressure | 5 | 270807 | 509 | 28208642 | 0.03 | -1.62 | 1.68 | 1.02 | 0.42 | 2.46 |
| Lacunar infarction | 5 | 270807 | 1720 | 28207431 | -1.62 | -3.27 | 0.03 | 0.32 | 0.13 | 0.78 |
| Mallory-weiss syndrome | 5 | 270807 | 937 | 28208214 | -0.78 | -2.44 | 0.87 | 0.58 | 0.24 | 1.39 |
| Mitral valve disease | 5 | 270807 | 933 | 28208218 | -0.78 | -2.43 | 0.88 | 0.58 | 0.24 | 1.40 |
| Placental infarction | 5 | 270807 | 78 | 28209073 | 2.09 | 0.44 | 3.75 | 4.40 | 1.78 | 10.88 |
| Placental insufficiency | 5 | 270807 | 419 | 28208732 | 0.28 | -1.38 | 1.93 | 1.22 | 0.50 | 2.94 |
| Postmenopausal haemorrhage | 5 | 270807 | 1399 | 28207752 | -1.33 | -2.99 | 0.32 | 0.39 | 0.16 | 0.95 |
| Renal ischaemia | 5 | 270807 | 276 | 28208875 | 0.79 | -0.86 | 2.45 | 1.75 | 0.72 | 4.23 |
| Renal vessel disorder | 5 | 270807 | 128 | 28209023 | 1.64 | -0.01 | 3.29 | 3.18 | 1.30 | 7.77 |
| Reversible cerebral vasoconstriction syndrome | 5 | 270807 | 1297 | 28207854 | -1.23 | -2.88 | 0.43 | 0.42 | 0.18 | 1.02 |
| Sinoatrial block | 5 | 270807 | 464 | 28208687 | 0.15 | -1.51 | 1.80 | 1.11 | 0.46 | 2.68 |
| Splenic haemorrhage | 5 | 270807 | 548 | 28208603 | -0.07 | -1.72 | 1.59 | 0.95 | 0.40 | 2.30 |
| Spontaneous haematoma | 5 | 270807 | 681 | 28208470 | -0.35 | -2.01 | 1.30 | 0.78 | 0.32 | 1.88 |
| Supine hypertension | 5 | 270807 | 356 | 28208795 | 0.48 | -1.17 | 2.14 | 1.40 | 0.58 | 3.39 |
| Systolic dysfunction | 5 | 270807 | 653 | 28208498 | -0.30 | -1.95 | 1.36 | 0.81 | 0.34 | 1.96 |
| Tooth socket haemorrhage | 5 | 270807 | 238 | 28208913 | 0.97 | -0.69 | 2.62 | 1.97 | 0.81 | 4.79 |
| Vein collapse | 5 | 270807 | 709 | 28208442 | -0.41 | -2.06 | 1.25 | 0.75 | 0.31 | 1.81 |
| Vein rupture | 5 | 270807 | 1358 | 28207793 | -1.29 | -2.95 | 0.36 | 0.41 | 0.17 | 0.98 |
| Venoocclusive liver disease | 5 | 270807 | 6284 | 28202867 | -3.45 | -5.11 | -1.80 | 0.09 | 0.04 | 0.22 |
| Anaphylactoid shock | 4 | 270808 | 325 | 28208826 | 0.31 | -1.59 | 2.21 | 1.24 | 0.46 | 3.33 |
| Anti-neutrophil cytoplasmic antibody positive vasculitis | 4 | 270808 | 780 | 28208371 | -0.82 | -2.72 | 1.08 | 0.56 | 0.21 | 1.50 |
| Aortic valve disease | 4 | 270808 | 640 | 28208511 | -0.56 | -2.46 | 1.34 | 0.68 | 0.25 | 1.81 |
| Atrial tachycardia | 4 | 270808 | 639 | 28208512 | -0.56 | -2.46 | 1.34 | 0.68 | 0.25 | 1.81 |
| Blood loss anaemia | 4 | 270808 | 523 | 28208628 | -0.29 | -2.19 | 1.61 | 0.82 | 0.30 | 2.18 |
| Bloody discharge | 4 | 270808 | 391 | 28208760 | 0.08 | -1.82 | 1.98 | 1.06 | 0.40 | 2.83 |
| Bradyarrhythmia | 4 | 270808 | 654 | 28208497 | -0.59 | -2.49 | 1.31 | 0.66 | 0.25 | 1.77 |
| Brain stem haemorrhage | 4 | 270808 | 787 | 28208364 | -0.83 | -2.73 | 1.07 | 0.56 | 0.21 | 1.49 |
| Bundle branch block left | 4 | 270808 | 1314 | 28207837 | -1.53 | -3.43 | 0.37 | 0.34 | 0.13 | 0.92 |
| Cardiotoxicity | 4 | 270808 | 3448 | 28205703 | -2.89 | -4.79 | -0.99 | 0.13 | 0.05 | 0.36 |
| Carotid artery disease | 4 | 270808 | 383 | 28208768 | 0.11 | -1.79 | 2.01 | 1.08 | 0.40 | 2.89 |
| Carotid artery occlusion | 4 | 270808 | 1143 | 28208008 | -1.34 | -3.24 | 0.56 | 0.39 | 0.15 | 1.05 |
| Cerebral vasoconstriction | 4 | 270808 | 257 | 28208894 | 0.59 | -1.31 | 2.49 | 1.52 | 0.56 | 4.07 |
| Cor pulmonale | 4 | 270808 | 362 | 28208789 | 0.18 | -1.72 | 2.08 | 1.13 | 0.42 | 3.03 |
| Coronary artery thrombosis | 4 | 270808 | 698 | 28208453 | -0.67 | -2.57 | 1.23 | 0.62 | 0.23 | 1.67 |
| Coronary vein stenosis | 4 | 270808 | 38 | 28209113 | 2.32 | 0.42 | 4.22 | 5.20 | 1.86 | 14.58 |
| Diabetic retinopathy | 4 | 270808 | 1145 | 28208006 | -1.34 | -3.24 | 0.56 | 0.39 | 0.15 | 1.05 |
| Dyspnoea at rest | 4 | 270808 | 1233 | 28207918 | -1.45 | -3.35 | 0.45 | 0.36 | 0.14 | 0.97 |
| Eye contusion | 4 | 270808 | 964 | 28208187 | -1.11 | -3.01 | 0.79 | 0.46 | 0.17 | 1.23 |
| Haemangioma | 4 | 270808 | 740 | 28208411 | -0.75 | -2.65 | 1.15 | 0.59 | 0.22 | 1.58 |
| Haemangioma of liver | 4 | 270808 | 328 | 28208823 | 0.30 | -1.60 | 2.20 | 1.23 | 0.46 | 3.31 |
| Haematospermia | 4 | 270808 | 443 | 28208708 | -0.08 | -1.98 | 1.82 | 0.95 | 0.35 | 2.53 |
| Haemorrhage subcutaneous | 4 | 270808 | 1901 | 28207250 | -2.05 | -3.95 | -0.15 | 0.24 | 0.09 | 0.64 |
| Heat stroke | 4 | 270808 | 783 | 28208368 | -0.83 | -2.73 | 1.07 | 0.56 | 0.21 | 1.50 |
| Hepatic congestion | 4 | 270808 | 449 | 28208702 | -0.10 | -2.00 | 1.80 | 0.94 | 0.35 | 2.50 |
| Infusion site haemorrhage | 4 | 270808 | 1204 | 28207947 | -1.41 | -3.31 | 0.49 | 0.37 | 0.14 | 1.00 |
| Laryngeal haematoma | 4 | 270808 | 45 | 28209106 | 2.22 | 0.32 | 4.12 | 4.83 | 1.74 | 13.43 |
| Left ventricular hypertrophy | 4 | 270808 | 1344 | 28207807 | -1.57 | -3.47 | 0.33 | 0.34 | 0.13 | 0.90 |
| Microangiopathy | 4 | 270808 | 492 | 28208659 | -0.21 | -2.11 | 1.69 | 0.86 | 0.32 | 2.30 |
| Mitral valve prolapse | 4 | 270808 | 770 | 28208381 | -0.80 | -2.70 | 1.10 | 0.57 | 0.21 | 1.52 |
| Mitral valve stenosis | 4 | 270808 | 255 | 28208896 | 0.60 | -1.30 | 2.50 | 1.53 | 0.57 | 4.10 |
| Naevus flammeus | 4 | 270808 | 111 | 28209040 | 1.50 | -0.40 | 3.40 | 2.87 | 1.06 | 7.79 |
| Oesophagitis haemorrhagic | 4 | 270808 | 564 | 28208587 | -0.39 | -2.29 | 1.51 | 0.76 | 0.28 | 2.03 |
| Optic ischaemic neuropathy | 4 | 270808 | 1218 | 28207933 | -1.43 | -3.33 | 0.47 | 0.37 | 0.14 | 0.98 |
| Patent ductus arteriosus | 4 | 270808 | 1555 | 28207596 | -1.77 | -3.67 | 0.13 | 0.29 | 0.11 | 0.78 |
| Portal shunt | 4 | 270808 | 77 | 28209074 | 1.82 | -0.08 | 3.72 | 3.63 | 1.33 | 9.92 |
| Postoperative thrombosis | 4 | 270808 | 288 | 28208863 | 0.46 | -1.44 | 2.36 | 1.38 | 0.51 | 3.70 |
| Procedural hypotension | 4 | 270808 | 583 | 28208568 | -0.43 | -2.33 | 1.47 | 0.74 | 0.28 | 1.97 |
| Pulmonary infarction | 4 | 270808 | 1052 | 28208099 | -1.23 | -3.13 | 0.67 | 0.42 | 0.16 | 1.13 |
| Pulmonary valve stenosis | 4 | 270808 | 339 | 28208812 | 0.26 | -1.64 | 2.16 | 1.20 | 0.45 | 3.21 |
| Puncture site haemorrhage | 4 | 270808 | 598 | 28208553 | -0.47 | -2.37 | 1.43 | 0.72 | 0.27 | 1.93 |
| Shunt thrombosis | 4 | 270808 | 102 | 28209049 | 1.58 | -0.32 | 3.48 | 3.04 | 1.12 | 8.26 |
| Splenic vein thrombosis | 4 | 270808 | 197 | 28208954 | 0.90 | -1.00 | 2.80 | 1.88 | 0.70 | 5.06 |
| Superior vena cava occlusion | 4 | 270808 | 231 | 28208920 | 0.72 | -1.18 | 2.62 | 1.66 | 0.62 | 4.45 |
| Takayasu’s arteritis | 4 | 270808 | 430 | 28208721 | -0.04 | -1.94 | 1.86 | 0.97 | 0.36 | 2.60 |
| Thrombotic cerebral infarction | 4 | 270808 | 897 | 28208254 | -1.01 | -2.91 | 0.89 | 0.49 | 0.18 | 1.32 |
| Toxic shock syndrome | 4 | 270808 | 362 | 28208789 | 0.18 | -1.72 | 2.08 | 1.13 | 0.42 | 3.03 |
| Traumatic intracranial haemorrhage | 4 | 270808 | 990 | 28208161 | -1.15 | -3.05 | 0.75 | 0.45 | 0.17 | 1.20 |
| Urogenital haemorrhage | 4 | 270808 | 355 | 28208796 | 0.20 | -1.70 | 2.10 | 1.15 | 0.43 | 3.09 |
| Vascular calcification | 4 | 270808 | 412 | 28208739 | 0.01 | -1.89 | 1.91 | 1.01 | 0.38 | 2.70 |
| Vascular insufficiency | 4 | 270808 | 290 | 28208861 | 0.45 | -1.45 | 2.35 | 1.37 | 0.51 | 3.68 |
| Vein discolouration | 4 | 270808 | 282 | 28208869 | 0.48 | -1.42 | 2.38 | 1.40 | 0.52 | 3.76 |
| Venous injury | 4 | 270808 | 413 | 28208738 | 0.01 | -1.89 | 1.91 | 1.01 | 0.38 | 2.70 |
| Accelerated hypertension | 3 | 270809 | 175 | 28208976 | 0.67 | -1.62 | 2.96 | 1.61 | 0.51 | 5.03 |
| Anastomotic haemorrhage | 3 | 270809 | 104 | 28209047 | 1.21 | -1.08 | 3.50 | 2.34 | 0.74 | 7.36 |
| Angina bullosa haemorrhagica | 3 | 270809 | 254 | 28208897 | 0.25 | -2.04 | 2.54 | 1.19 | 0.38 | 3.72 |
| Aortic valve sclerosis | 3 | 270809 | 135 | 28209016 | 0.95 | -1.34 | 3.24 | 1.95 | 0.62 | 6.12 |
| Arrhythmia supraventricular | 3 | 270809 | 409 | 28208742 | -0.34 | -2.63 | 1.95 | 0.79 | 0.25 | 2.46 |
| Arterial disorder | 3 | 270809 | 759 | 28208392 | -1.15 | -3.44 | 1.14 | 0.45 | 0.14 | 1.40 |
| Arterial insufficiency | 3 | 270809 | 108 | 28209043 | 1.17 | -1.12 | 3.46 | 2.28 | 0.72 | 7.17 |
| Arterial stenosis | 3 | 270809 | 417 | 28208734 | -0.36 | -2.65 | 1.93 | 0.78 | 0.25 | 2.42 |
| Arterial thrombosis | 3 | 270809 | 779 | 28208372 | -1.18 | -3.47 | 1.11 | 0.44 | 0.14 | 1.36 |
| Arteriovenous fistula site haemorrhage | 3 | 270809 | 184 | 28208967 | 0.62 | -1.67 | 2.91 | 1.54 | 0.49 | 4.83 |
| Autonomic nervous system imbalance | 3 | 270809 | 858 | 28208293 | -1.31 | -3.60 | 0.98 | 0.40 | 0.13 | 1.24 |
| Capillary leak syndrome | 3 | 270809 | 741 | 28208410 | -1.11 | -3.40 | 1.18 | 0.46 | 0.15 | 1.43 |
| Cardiac discomfort | 3 | 270809 | 684 | 28208467 | -1.01 | -3.30 | 1.28 | 0.50 | 0.16 | 1.54 |
| Carotid arteriosclerosis | 3 | 270809 | 298 | 28208853 | 0.06 | -2.23 | 2.35 | 1.04 | 0.33 | 3.25 |
| Catheter site haemorrhage | 3 | 270809 | 1113 | 28208038 | -1.67 | -3.96 | 0.62 | 0.31 | 0.10 | 0.97 |
| Cerebral venous thrombosis | 3 | 270809 | 936 | 28208215 | -1.43 | -3.72 | 0.86 | 0.37 | 0.12 | 1.15 |
| Coronary artery embolism | 3 | 270809 | 126 | 28209025 | 1.02 | -1.27 | 3.31 | 2.05 | 0.65 | 6.43 |
| Cushingoid | 3 | 270809 | 1081 | 28208070 | -1.63 | -3.92 | 0.66 | 0.32 | 0.10 | 1.00 |
| Device related thrombosis | 3 | 270809 | 572 | 28208579 | -0.77 | -3.06 | 1.52 | 0.58 | 0.19 | 1.82 |
| Diabetic ulcer | 3 | 270809 | 543 | 28208608 | -0.70 | -2.99 | 1.59 | 0.61 | 0.20 | 1.91 |
| Extradural haematoma | 3 | 270809 | 684 | 28208467 | -1.01 | -3.30 | 1.28 | 0.50 | 0.16 | 1.54 |
| Foetal arrhythmia | 3 | 270809 | 136 | 28209015 | 0.94 | -1.35 | 3.23 | 1.94 | 0.62 | 6.09 |
| Gastric varices | 3 | 270809 | 150 | 28209001 | 0.84 | -1.45 | 3.13 | 1.80 | 0.58 | 5.66 |
| Granulomatosis with polyangiitis | 3 | 270809 | 507 | 28208644 | -0.61 | -2.90 | 1.68 | 0.65 | 0.21 | 2.03 |
| Haemobilia | 3 | 270809 | 265 | 28208886 | 0.20 | -2.09 | 2.49 | 1.15 | 0.37 | 3.59 |
| Haemodynamic instability | 3 | 270809 | 2082 | 28207069 | -2.54 | -4.83 | -0.25 | 0.17 | 0.06 | 0.53 |
| Haemorrhagic infarction | 3 | 270809 | 355 | 28208796 | -0.16 | -2.45 | 2.13 | 0.90 | 0.29 | 2.79 |
| Heart disease congenital | 3 | 270809 | 1502 | 28207649 | -2.08 | -4.37 | 0.21 | 0.23 | 0.08 | 0.73 |
| Hellp syndrome | 3 | 270809 | 223 | 28208928 | 0.40 | -1.89 | 2.69 | 1.33 | 0.42 | 4.14 |
| Heparin-induced thrombocytopenia | 3 | 270809 | 1484 | 28207667 | -2.06 | -4.35 | 0.23 | 0.24 | 0.08 | 0.74 |
| Hepatic fibrosis | 3 | 270809 | 2590 | 28206561 | -2.85 | -5.14 | -0.56 | 0.14 | 0.04 | 0.43 |
| Intermittent claudication | 3 | 270809 | 645 | 28208506 | -0.93 | -3.22 | 1.36 | 0.52 | 0.17 | 1.63 |
| Ischaemic nephropathy | 3 | 270809 | 21 | 28209130 | 2.26 | -0.03 | 4.56 | 4.99 | 1.49 | 16.72 |
| Left ventricle outflow tract obstruction | 3 | 270809 | 256 | 28208895 | 0.24 | -2.05 | 2.53 | 1.18 | 0.38 | 3.69 |
| Malignant hypertension | 3 | 270809 | 631 | 28208520 | -0.90 | -3.19 | 1.39 | 0.53 | 0.17 | 1.66 |
| Malignant renal hypertension | 3 | 270809 | 26 | 28209125 | 2.17 | -0.12 | 4.46 | 4.67 | 1.41 | 15.43 |
| Penile contusion | 3 | 270809 | 264 | 28208887 | 0.20 | -2.09 | 2.49 | 1.15 | 0.37 | 3.60 |
| Penile haematoma | 3 | 270809 | 212 | 28208939 | 0.46 | -1.83 | 2.75 | 1.38 | 0.44 | 4.31 |
| Pharyngeal haematoma | 3 | 270809 | 86 | 28209065 | 1.38 | -0.91 | 3.67 | 2.64 | 0.84 | 8.35 |
| Portal hypertensive gastropathy | 3 | 270809 | 230 | 28208921 | 0.37 | -1.92 | 2.66 | 1.29 | 0.41 | 4.04 |
| Post procedural contusion | 3 | 270809 | 239 | 28208912 | 0.32 | -1.97 | 2.61 | 1.25 | 0.40 | 3.91 |
| Post thrombotic syndrome | 3 | 270809 | 298 | 28208853 | 0.06 | -2.23 | 2.35 | 1.04 | 0.33 | 3.25 |
| Pulmonary oil microembolism | 3 | 270809 | 100 | 28209051 | 1.24 | -1.05 | 3.53 | 2.40 | 0.76 | 7.56 |
| Renal artery arteriosclerosis | 3 | 270809 | 126 | 28209025 | 1.02 | -1.27 | 3.31 | 2.05 | 0.65 | 6.43 |
| Renal artery occlusion | 3 | 270809 | 134 | 28209017 | 0.96 | -1.33 | 3.25 | 1.96 | 0.62 | 6.15 |
| Renal vein thrombosis | 3 | 270809 | 284 | 28208867 | 0.12 | -2.17 | 2.41 | 1.08 | 0.35 | 3.38 |
| Restrictive cardiomyopathy | 3 | 270809 | 263 | 28208888 | 0.21 | -2.08 | 2.50 | 1.16 | 0.37 | 3.61 |
| Retinal exudates | 3 | 270809 | 311 | 28208840 | 0.01 | -2.28 | 2.30 | 1.00 | 0.32 | 3.13 |
| Retinal vein thrombosis | 3 | 270809 | 390 | 28208761 | -0.28 | -2.57 | 2.01 | 0.82 | 0.26 | 2.57 |
| Rheumatoid vasculitis | 3 | 270809 | 210 | 28208941 | 0.47 | -1.82 | 2.76 | 1.39 | 0.45 | 4.35 |
| Right-to-left cardiac shunt | 3 | 270809 | 154 | 28208997 | 0.81 | -1.48 | 3.10 | 1.77 | 0.56 | 5.55 |
| Secondary hypertension | 3 | 270809 | 240 | 28208911 | 0.32 | -1.97 | 2.61 | 1.25 | 0.40 | 3.90 |
| Septic embolus | 3 | 270809 | 356 | 28208795 | -0.16 | -2.45 | 2.13 | 0.89 | 0.29 | 2.78 |
| Shock symptom | 3 | 270809 | 315 | 28208836 | -0.01 | -2.30 | 2.28 | 0.99 | 0.32 | 3.10 |
| Small intestinal ulcer haemorrhage | 3 | 270809 | 173 | 28208978 | 0.69 | -1.60 | 2.98 | 1.62 | 0.52 | 5.07 |
| Spider naevus | 3 | 270809 | 180 | 28208971 | 0.64 | -1.65 | 2.93 | 1.57 | 0.50 | 4.92 |
| Splenic artery aneurysm | 3 | 270809 | 162 | 28208989 | 0.76 | -1.53 | 3.05 | 1.70 | 0.54 | 5.34 |
| Stasis dermatitis | 3 | 270809 | 631 | 28208520 | -0.90 | -3.19 | 1.39 | 0.53 | 0.17 | 1.66 |
| Stroke in evolution | 3 | 270809 | 476 | 28208675 | -0.53 | -2.82 | 1.76 | 0.69 | 0.22 | 2.15 |
| Superficial vein prominence | 3 | 270809 | 135 | 28209016 | 0.95 | -1.34 | 3.24 | 1.95 | 0.62 | 6.12 |
| Superior sagittal sinus thrombosis | 3 | 270809 | 359 | 28208792 | -0.17 | -2.46 | 2.12 | 0.89 | 0.28 | 2.76 |
| Tachycardia paroxysmal | 3 | 270809 | 445 | 28208706 | -0.44 | -2.73 | 1.85 | 0.73 | 0.24 | 2.28 |
| Thalamus haemorrhage | 3 | 270809 | 1713 | 28207438 | -2.26 | -4.55 | 0.03 | 0.21 | 0.07 | 0.64 |
| Thrombosed varicose vein | 3 | 270809 | 35 | 28209116 | 2.02 | -0.27 | 4.31 | 4.19 | 1.29 | 13.61 |
| Tracheal haemorrhage | 3 | 270809 | 370 | 28208781 | -0.21 | -2.50 | 2.08 | 0.86 | 0.28 | 2.69 |
| Trifascicular block | 3 | 270809 | 164 | 28208987 | 0.75 | -1.54 | 3.04 | 1.69 | 0.54 | 5.29 |
| Truncus coeliacus thrombosis | 3 | 270809 | 21 | 28209130 | 2.26 | -0.03 | 4.56 | 4.99 | 1.49 | 16.72 |
| Varicose ulceration | 3 | 270809 | 195 | 28208956 | 0.55 | -1.74 | 2.84 | 1.48 | 0.47 | 4.61 |
| Vascular encephalopathy | 3 | 270809 | 200 | 28208951 | 0.53 | -1.76 | 2.82 | 1.45 | 0.46 | 4.52 |
| Vascular malformation | 3 | 270809 | 277 | 28208874 | 0.15 | -2.14 | 2.44 | 1.11 | 0.36 | 3.46 |
| Vena cava thrombosis | 3 | 270809 | 1506 | 28207645 | -2.08 | -4.38 | 0.21 | 0.23 | 0.08 | 0.73 |
| Ventricular flutter | 3 | 270809 | 133 | 28209018 | 0.96 | -1.33 | 3.25 | 1.97 | 0.63 | 6.19 |
| Wolff-parkinson-white syndrome | 3 | 270809 | 467 | 28208684 | -0.51 | -2.80 | 1.78 | 0.70 | 0.23 | 2.19 |

**Table S3.** Disproportionality results of cardiac and vascular toxicities associated with lansoprazole

| PT | a | b | c | d | IC | IC_025_ | IC_975_ | ROR | ROR_025_ | ROR_975_ |
| --- | --- | --- | --- | --- | --- | --- | --- | --- | --- | --- |
| Dyspnoea | 334 | 109308 | 217815 | 28152506 | -1.33 | -1.51 | -1.15 | 0.40 | 0.36 | 0.44 |
| Renal haemangioma | 315 | 109327 | 1767 | 28368554 | 5.21 | 5.03 | 5.40 | 43.16 | 38.28 | 48.67 |
| Syncope | 281 | 109361 | 75034 | 28295287 | -0.05 | -0.24 | 0.15 | 0.97 | 0.86 | 1.09 |
| Dizziness | 221 | 109421 | 172615 | 28197706 | -1.59 | -1.81 | -1.37 | 0.33 | 0.29 | 0.38 |
| Hypertension | 187 | 109455 | 87977 | 28282344 | -0.86 | -1.10 | -0.62 | 0.55 | 0.48 | 0.63 |
| Myocardial infarction | 183 | 109459 | 96147 | 28274174 | -1.02 | -1.26 | -0.77 | 0.49 | 0.43 | 0.57 |
| Gastrointestinal haemorrhage | 174 | 109468 | 118595 | 28251726 | -1.39 | -1.64 | -1.14 | 0.38 | 0.33 | 0.44 |
| Pulmonary embolism | 172 | 109470 | 69903 | 28300418 | -0.65 | -0.90 | -0.40 | 0.64 | 0.55 | 0.74 |
| Hypotension | 169 | 109473 | 87299 | 28283022 | -0.99 | -1.25 | -0.74 | 0.50 | 0.43 | 0.58 |
| Anaphylactic reaction | 168 | 109474 | 25520 | 28344801 | 0.76 | 0.51 | 1.02 | 1.70 | 1.46 | 1.98 |
| Peripheral swelling | 166 | 109476 | 104425 | 28265896 | -1.28 | -1.53 | -1.02 | 0.41 | 0.35 | 0.48 |
| Palpitations | 165 | 109477 | 52604 | 28317717 | -0.30 | -0.56 | -0.04 | 0.81 | 0.70 | 0.95 |
| Rectal haemorrhage | 158 | 109484 | 47844 | 28322477 | -0.23 | -0.49 | 0.04 | 0.85 | 0.73 | 1.00 |
| Chest pain | 138 | 109504 | 67873 | 28302448 | -0.92 | -1.20 | -0.64 | 0.53 | 0.45 | 0.62 |
| Oedema peripheral | 128 | 109514 | 50267 | 28320054 | -0.60 | -0.89 | -0.31 | 0.66 | 0.55 | 0.78 |
| Tachycardia | 126 | 109516 | 44521 | 28325800 | -0.45 | -0.74 | -0.15 | 0.73 | 0.62 | 0.87 |
| Osteonecrosis of jaw | 125 | 109517 | 26498 | 28343823 | 0.29 | -0.01 | 0.58 | 1.22 | 1.02 | 1.45 |
| Thrombosis | 119 | 109523 | 63561 | 28306760 | -1.04 | -1.34 | -0.74 | 0.48 | 0.41 | 0.58 |
| Upper gastrointestinal haemorrhage | 118 | 109524 | 38409 | 28331912 | -0.33 | -0.63 | -0.02 | 0.80 | 0.66 | 0.95 |
| Subdural haematoma | 110 | 109532 | 20578 | 28349743 | 0.46 | 0.15 | 0.78 | 1.38 | 1.15 | 1.67 |
| Melaena | 107 | 109535 | 15204 | 28355117 | 0.85 | 0.53 | 1.18 | 1.81 | 1.50 | 2.20 |
| Septic shock | 104 | 109538 | 38287 | 28332034 | -0.50 | -0.83 | -0.18 | 0.70 | 0.58 | 0.85 |
| Cerebral infarction | 99 | 109543 | 16068 | 28354253 | 0.67 | 0.33 | 1.00 | 1.59 | 1.30 | 1.94 |
| Orthostatic hypotension | 99 | 109543 | 12425 | 28357896 | 1.03 | 0.70 | 1.36 | 2.05 | 1.68 | 2.50 |
| Cardiac failure | 93 | 109549 | 38289 | 28332032 | -0.67 | -1.01 | -0.32 | 0.63 | 0.51 | 0.77 |
| Transient ischaemic attack | 91 | 109551 | 30301 | 28340020 | -0.36 | -0.71 | -0.01 | 0.78 | 0.63 | 0.96 |
| Atrial fibrillation | 82 | 109560 | 44995 | 28325326 | -1.08 | -1.44 | -0.71 | 0.47 | 0.38 | 0.59 |
| Migraine | 81 | 109561 | 42502 | 28327819 | -1.01 | -1.38 | -0.64 | 0.49 | 0.40 | 0.61 |
| Haematuria | 80 | 109562 | 23186 | 28347135 | -0.16 | -0.53 | 0.21 | 0.89 | 0.72 | 1.11 |
| Ischaemic stroke | 73 | 109569 | 18163 | 28352158 | 0.06 | -0.33 | 0.45 | 1.04 | 0.83 | 1.31 |
| Subarachnoid haemorrhage | 72 | 109570 | 10101 | 28360220 | 0.87 | 0.48 | 1.26 | 1.83 | 1.45 | 2.31 |
| Cerebrovascular accident | 71 | 109571 | 78055 | 28292266 | -2.08 | -2.47 | -1.68 | 0.24 | 0.19 | 0.30 |
| Haemorrhage | 71 | 109571 | 44620 | 28325701 | -1.27 | -1.67 | -0.88 | 0.41 | 0.33 | 0.52 |
| Presyncope | 70 | 109572 | 15091 | 28355230 | 0.26 | -0.14 | 0.66 | 1.20 | 0.95 | 1.52 |
| Cerebral haemorrhage | 69 | 109573 | 23979 | 28346342 | -0.42 | -0.82 | -0.02 | 0.75 | 0.59 | 0.94 |
| Haematemesis | 64 | 109578 | 12368 | 28357953 | 0.42 | 0.00 | 0.83 | 1.34 | 1.04 | 1.71 |
| Skin ulcer | 60 | 109582 | 18253 | 28352068 | -0.23 | -0.66 | 0.20 | 0.85 | 0.66 | 1.10 |
| Chest discomfort | 58 | 109584 | 32134 | 28338187 | -1.09 | -1.53 | -0.65 | 0.47 | 0.36 | 0.61 |
| Hypertensive nephropathy | 56 | 109586 | 354 | 28369967 | 4.76 | 4.32 | 5.21 | 30.26 | 22.82 | 40.11 |
| Haemoptysis | 53 | 109589 | 18428 | 28351893 | -0.42 | -0.88 | 0.04 | 0.75 | 0.57 | 0.98 |
| Bradycardia | 52 | 109590 | 19765 | 28350556 | -0.55 | -1.01 | -0.09 | 0.68 | 0.52 | 0.90 |
| Epistaxis | 52 | 109590 | 40009 | 28330312 | -1.56 | -2.02 | -1.10 | 0.34 | 0.26 | 0.44 |
| Deep vein thrombosis | 51 | 109591 | 33195 | 28337126 | -1.32 | -1.79 | -0.85 | 0.40 | 0.30 | 0.53 |
| Pulmonary oedema | 51 | 109591 | 27732 | 28342589 | -1.06 | -1.53 | -0.59 | 0.48 | 0.36 | 0.63 |
| Cardiac failure congestive | 50 | 109592 | 43128 | 28327193 | -1.72 | -2.20 | -1.25 | 0.30 | 0.23 | 0.40 |
| Acute myocardial infarction | 48 | 109594 | 21884 | 28348437 | -0.81 | -1.29 | -0.33 | 0.57 | 0.43 | 0.76 |
| Cardiac arrest | 48 | 109594 | 31364 | 28338957 | -1.32 | -1.81 | -0.84 | 0.40 | 0.30 | 0.53 |
| Ventricular fibrillation | 45 | 109597 | 8395 | 28361926 | 0.46 | -0.03 | 0.96 | 1.38 | 1.03 | 1.85 |
| Contusion | 44 | 109598 | 35294 | 28335027 | -1.62 | -2.12 | -1.11 | 0.32 | 0.24 | 0.44 |
| Shock | 43 | 109599 | 10740 | 28359581 | 0.05 | -0.46 | 0.56 | 1.04 | 0.77 | 1.40 |
| Hot flush | 39 | 109603 | 27990 | 28342331 | -1.46 | -1.99 | -0.92 | 0.36 | 0.27 | 0.50 |
| Pulmonary arterial hypertension | 39 | 109603 | 13853 | 28356468 | -0.45 | -0.99 | 0.09 | 0.73 | 0.53 | 1.00 |
| Embolic stroke | 38 | 109604 | 3342 | 28366979 | 1.51 | 0.97 | 2.05 | 2.87 | 2.08 | 3.95 |
| Flushing | 38 | 109604 | 30327 | 28339994 | -1.61 | -2.15 | -1.06 | 0.33 | 0.24 | 0.45 |
| Sudden death | 38 | 109604 | 11060 | 28359261 | -0.17 | -0.71 | 0.38 | 0.89 | 0.65 | 1.22 |
| Haematoma | 37 | 109605 | 14716 | 28355605 | -0.61 | -1.16 | -0.06 | 0.65 | 0.47 | 0.90 |
| Lower gastrointestinal haemorrhage | 37 | 109605 | 17879 | 28352442 | -0.89 | -1.44 | -0.34 | 0.54 | 0.39 | 0.74 |
| Anaphylactic shock | 36 | 109606 | 13015 | 28357306 | -0.48 | -1.03 | 0.08 | 0.72 | 0.52 | 1.00 |
| Cardio-respiratory arrest | 36 | 109606 | 17193 | 28353128 | -0.87 | -1.43 | -0.31 | 0.55 | 0.39 | 0.76 |
| Sinus bradycardia | 36 | 109606 | 6196 | 28364125 | 0.58 | 0.02 | 1.13 | 1.49 | 1.08 | 2.07 |
| Angina pectoris | 35 | 109607 | 12435 | 28357886 | -0.45 | -1.02 | 0.12 | 0.73 | 0.52 | 1.02 |
| Haematochezia | 35 | 109607 | 24434 | 28345887 | -1.42 | -1.98 | -0.85 | 0.37 | 0.27 | 0.52 |
| Coronary artery disease | 33 | 109609 | 13768 | 28356553 | -0.68 | -1.26 | -0.09 | 0.62 | 0.44 | 0.88 |
| Ventricular tachycardia | 32 | 109610 | 12771 | 28357550 | -0.62 | -1.21 | -0.02 | 0.65 | 0.46 | 0.92 |
| Shock haemorrhagic | 31 | 109611 | 9147 | 28361174 | -0.19 | -0.79 | 0.42 | 0.88 | 0.62 | 1.25 |
| Vasculitis | 31 | 109611 | 7330 | 28362991 | 0.13 | -0.48 | 0.73 | 1.09 | 0.77 | 1.56 |
| Thrombotic microangiopathy | 30 | 109612 | 7777 | 28362544 | 0.00 | -0.62 | 0.61 | 1.00 | 0.70 | 1.43 |
| Pallor | 29 | 109613 | 8763 | 28361558 | -0.22 | -0.85 | 0.41 | 0.86 | 0.60 | 1.24 |
| Ventricular extrasystoles | 29 | 109613 | 6304 | 28364017 | 0.25 | -0.38 | 0.87 | 1.19 | 0.82 | 1.71 |
| Torsade de pointes | 28 | 109614 | 5436 | 28364885 | 0.40 | -0.23 | 1.04 | 1.33 | 0.91 | 1.92 |
| Myocardial ischaemia | 27 | 109615 | 5812 | 28364509 | 0.26 | -0.39 | 0.91 | 1.20 | 0.82 | 1.75 |
| Pericardial effusion | 27 | 109615 | 12259 | 28358062 | -0.80 | -1.45 | -0.15 | 0.57 | 0.39 | 0.84 |
| Pulmonary hypertension | 27 | 109615 | 14385 | 28355936 | -1.03 | -1.67 | -0.38 | 0.49 | 0.34 | 0.71 |
| Cardiac disorder | 25 | 109617 | 32358 | 28337963 | -2.30 | -2.97 | -1.62 | 0.20 | 0.14 | 0.30 |
| Disseminated intravascular coagulation | 25 | 109617 | 5053 | 28365268 | 0.35 | -0.33 | 1.02 | 1.27 | 0.86 | 1.89 |
| Gastric haemorrhage | 25 | 109617 | 6001 | 28364320 | 0.11 | -0.57 | 0.78 | 1.08 | 0.73 | 1.59 |
| Arrhythmia | 24 | 109618 | 17094 | 28353227 | -1.44 | -2.13 | -0.75 | 0.37 | 0.25 | 0.55 |
| Myocarditis | 24 | 109618 | 5749 | 28364572 | 0.11 | -0.58 | 0.80 | 1.08 | 0.72 | 1.61 |
| Renal artery stenosis | 23 | 109619 | 665 | 28369656 | 2.90 | 2.19 | 3.61 | 7.66 | 5.05 | 11.60 |
| Right ventricular failure | 23 | 109619 | 7403 | 28362918 | -0.31 | -1.01 | 0.40 | 0.81 | 0.54 | 1.22 |
| Gingival bleeding | 22 | 109620 | 5360 | 28364961 | 0.08 | -0.64 | 0.81 | 1.06 | 0.70 | 1.61 |
| Vaginal haemorrhage | 22 | 109620 | 26985 | 28343336 | -2.22 | -2.94 | -1.49 | 0.21 | 0.14 | 0.33 |
| Injection site bruising | 21 | 109621 | 29549 | 28340772 | -2.41 | -3.15 | -1.67 | 0.19 | 0.12 | 0.29 |
| Osteonecrosis | 21 | 109621 | 9623 | 28360698 | -0.81 | -1.55 | -0.07 | 0.57 | 0.37 | 0.88 |
| Haemorrhage intracranial | 20 | 109622 | 13816 | 28356505 | -1.39 | -2.15 | -0.63 | 0.38 | 0.25 | 0.59 |
| Supraventricular tachycardia | 20 | 109622 | 7827 | 28362494 | -0.58 | -1.34 | 0.18 | 0.67 | 0.43 | 1.03 |
| Circulatory collapse | 19 | 109623 | 6441 | 28363880 | -0.38 | -1.16 | 0.40 | 0.77 | 0.49 | 1.20 |
| Dyspnoea exertional | 19 | 109623 | 15024 | 28355297 | -1.58 | -2.36 | -0.80 | 0.33 | 0.21 | 0.52 |
| Subdural haemorrhage | 19 | 109623 | 3390 | 28366931 | 0.52 | -0.26 | 1.30 | 1.43 | 0.91 | 2.25 |
| Tumour haemorrhage | 19 | 109623 | 2576 | 28367745 | 0.89 | 0.11 | 1.68 | 1.87 | 1.19 | 2.93 |
| Venous thrombosis limb | 19 | 109623 | 2749 | 28367572 | 0.81 | 0.02 | 1.59 | 1.75 | 1.12 | 2.75 |
| Cardiac tamponade | 18 | 109624 | 2718 | 28367603 | 0.75 | -0.06 | 1.55 | 1.68 | 1.06 | 2.67 |
| Internal haemorrhage | 18 | 109624 | 19688 | 28350633 | -2.05 | -2.85 | -1.24 | 0.24 | 0.15 | 0.38 |
| Renal infarct | 18 | 109624 | 722 | 28369599 | 2.47 | 1.66 | 3.27 | 5.62 | 3.52 | 8.98 |
| Mouth haemorrhage | 17 | 109625 | 3861 | 28366460 | 0.18 | -0.65 | 1.01 | 1.13 | 0.70 | 1.83 |
| Pulmonary alveolar haemorrhage | 17 | 109625 | 3965 | 28366356 | 0.14 | -0.68 | 0.97 | 1.11 | 0.69 | 1.78 |
| Acute coronary syndrome | 16 | 109626 | 4868 | 28365453 | -0.23 | -1.08 | 0.63 | 0.85 | 0.52 | 1.40 |
| Colitis ischaemic | 16 | 109626 | 3826 | 28366495 | 0.11 | -0.75 | 0.97 | 1.08 | 0.66 | 1.76 |
| Thrombotic cerebral infarction | 16 | 109626 | 885 | 28369436 | 2.06 | 1.20 | 2.91 | 4.21 | 2.57 | 6.90 |
| Cardiogenic shock | 15 | 109627 | 4830 | 28365491 | -0.31 | -1.19 | 0.58 | 0.81 | 0.49 | 1.34 |
| Embolism | 15 | 109627 | 5664 | 28364657 | -0.53 | -1.42 | 0.36 | 0.69 | 0.42 | 1.15 |
| Hypertensive crisis | 15 | 109627 | 8067 | 28362254 | -1.03 | -1.91 | -0.14 | 0.49 | 0.29 | 0.81 |
| Pericarditis | 15 | 109627 | 6163 | 28364158 | -0.65 | -1.53 | 0.24 | 0.64 | 0.38 | 1.06 |
| Ulcer haemorrhage | 15 | 109627 | 5936 | 28364385 | -0.59 | -1.48 | 0.29 | 0.66 | 0.40 | 1.10 |
| Vascular pseudoaneurysm | 15 | 109627 | 2018 | 28368303 | 0.90 | 0.01 | 1.78 | 1.87 | 1.12 | 3.10 |
| Embolism venous | 14 | 109628 | 1522 | 28368799 | 1.18 | 0.26 | 2.10 | 2.27 | 1.34 | 3.85 |
| Hypersensitivity vasculitis | 14 | 109628 | 3664 | 28366657 | -0.02 | -0.94 | 0.90 | 0.99 | 0.59 | 1.67 |
| Pulmonary haemorrhage | 14 | 109628 | 5662 | 28364659 | -0.62 | -1.54 | 0.30 | 0.65 | 0.38 | 1.09 |
| Purpura | 14 | 109628 | 4368 | 28365953 | -0.26 | -1.18 | 0.66 | 0.83 | 0.49 | 1.41 |
| Sinus tachycardia | 14 | 109628 | 6674 | 28363647 | -0.86 | -1.78 | 0.06 | 0.55 | 0.33 | 0.93 |
| Stress cardiomyopathy | 14 | 109628 | 4865 | 28365456 | -0.41 | -1.33 | 0.51 | 0.75 | 0.44 | 1.27 |
| Atrioventricular block complete | 13 | 109629 | 3181 | 28367140 | 0.08 | -0.88 | 1.04 | 1.06 | 0.61 | 1.82 |
| Cyanosis | 13 | 109629 | 4125 | 28366196 | -0.28 | -1.24 | 0.67 | 0.82 | 0.48 | 1.42 |
| Gastric ulcer haemorrhage | 13 | 109629 | 4887 | 28365434 | -0.52 | -1.48 | 0.44 | 0.70 | 0.40 | 1.20 |
| Haemodynamic instability | 13 | 109629 | 2072 | 28368249 | 0.66 | -0.30 | 1.62 | 1.59 | 0.92 | 2.74 |
| Haemorrhage subcutaneous | 13 | 109629 | 1892 | 28368429 | 0.79 | -0.17 | 1.74 | 1.73 | 1.00 | 2.98 |
| Injection site haemorrhage | 13 | 109629 | 24893 | 28345428 | -2.84 | -3.79 | -1.88 | 0.14 | 0.08 | 0.24 |
| Intestinal ischaemia | 13 | 109629 | 2881 | 28367440 | 0.21 | -0.74 | 1.17 | 1.16 | 0.67 | 2.00 |
| Muscle haemorrhage | 13 | 109629 | 3085 | 28367236 | 0.12 | -0.84 | 1.08 | 1.09 | 0.63 | 1.87 |
| Pericardial haemorrhage | 13 | 109629 | 3160 | 28367161 | 0.09 | -0.87 | 1.04 | 1.06 | 0.62 | 1.83 |
| Renal hypertension | 13 | 109629 | 362 | 28369959 | 2.80 | 1.84 | 3.75 | 7.11 | 4.09 | 12.36 |
| Sinus node dysfunction | 13 | 109629 | 2848 | 28367473 | 0.23 | -0.73 | 1.19 | 1.17 | 0.68 | 2.02 |
| Sudden cardiac death | 13 | 109629 | 2808 | 28367513 | 0.25 | -0.71 | 1.21 | 1.19 | 0.69 | 2.05 |
| Ventricular septal defect | 13 | 109629 | 4603 | 28365718 | -0.44 | -1.39 | 0.52 | 0.74 | 0.43 | 1.27 |
| Cardiovascular disorder | 12 | 109630 | 6163 | 28364158 | -0.96 | -1.96 | 0.04 | 0.51 | 0.29 | 0.91 |
| Diarrhoea haemorrhagic | 12 | 109630 | 3899 | 28366422 | -0.32 | -1.32 | 0.69 | 0.80 | 0.46 | 1.42 |
| Haemorrhage urinary tract | 12 | 109630 | 1681 | 28368640 | 0.83 | -0.17 | 1.83 | 1.79 | 1.01 | 3.15 |
| Peripheral arterial occlusive disease | 12 | 109630 | 3224 | 28367097 | -0.05 | -1.05 | 0.95 | 0.96 | 0.55 | 1.70 |
| Pulmonary congestion | 12 | 109630 | 6235 | 28364086 | -0.97 | -1.97 | 0.03 | 0.51 | 0.29 | 0.90 |
| Pulmonary thrombosis | 12 | 109630 | 7493 | 28362828 | -1.23 | -2.23 | -0.23 | 0.42 | 0.24 | 0.75 |
| Thalamus haemorrhage | 12 | 109630 | 1704 | 28368617 | 0.81 | -0.19 | 1.82 | 1.76 | 1.00 | 3.11 |
| Tricuspid valve incompetence | 12 | 109630 | 3023 | 28367298 | 0.04 | -0.96 | 1.04 | 1.03 | 0.58 | 1.81 |
| Venous occlusion | 12 | 109630 | 1385 | 28368936 | 1.09 | 0.09 | 2.09 | 2.14 | 1.21 | 3.77 |
| Cardiac failure acute | 11 | 109631 | 4228 | 28366093 | -0.55 | -1.60 | 0.50 | 0.68 | 0.38 | 1.23 |
| Coronary artery stenosis | 11 | 109631 | 2043 | 28368278 | 0.45 | -0.60 | 1.50 | 1.37 | 0.76 | 2.48 |
| Dizziness postural | 11 | 109631 | 3468 | 28366853 | -0.27 | -1.32 | 0.78 | 0.83 | 0.46 | 1.50 |
| Embolic cerebral infarction | 11 | 109631 | 672 | 28369649 | 1.88 | 0.83 | 2.93 | 3.71 | 2.05 | 6.74 |
| Fluid overload | 11 | 109631 | 10324 | 28359997 | -1.81 | -2.86 | -0.76 | 0.28 | 0.16 | 0.51 |
| Haemorrhoidal haemorrhage | 11 | 109631 | 4581 | 28365740 | -0.66 | -1.71 | 0.39 | 0.63 | 0.35 | 1.14 |
| Mitral valve incompetence | 11 | 109631 | 3867 | 28366454 | -0.42 | -1.47 | 0.63 | 0.74 | 0.41 | 1.35 |
| Peripheral coldness | 11 | 109631 | 6063 | 28364258 | -1.05 | -2.10 | 0.00 | 0.48 | 0.27 | 0.87 |
| Prerenal failure | 11 | 109631 | 1441 | 28368880 | 0.92 | -0.13 | 1.97 | 1.89 | 1.05 | 3.43 |
| Putamen haemorrhage | 11 | 109631 | 565 | 28369756 | 2.08 | 1.03 | 3.13 | 4.29 | 2.36 | 7.78 |
| Retinal haemorrhage | 11 | 109631 | 4019 | 28366302 | -0.48 | -1.53 | 0.57 | 0.72 | 0.40 | 1.30 |
| Thrombotic thrombocytopenic purpura | 11 | 109631 | 2933 | 28367388 | -0.04 | -1.09 | 1.01 | 0.97 | 0.54 | 1.76 |
| Arterial occlusive disease | 10 | 109632 | 3114 | 28367207 | -0.25 | -1.36 | 0.85 | 0.84 | 0.45 | 1.56 |
| Cardiomyopathy | 10 | 109632 | 6245 | 28364076 | -1.23 | -2.33 | -0.12 | 0.43 | 0.23 | 0.79 |
| Lacunar infarction | 10 | 109632 | 1715 | 28368606 | 0.56 | -0.55 | 1.66 | 1.47 | 0.79 | 2.74 |
| Large intestinal haemorrhage | 10 | 109632 | 1974 | 28368347 | 0.37 | -0.74 | 1.47 | 1.29 | 0.69 | 2.40 |
| Small intestinal haemorrhage | 10 | 109632 | 2737 | 28367584 | -0.08 | -1.18 | 1.03 | 0.95 | 0.51 | 1.76 |
| Stoma site haemorrhage | 10 | 109632 | 1388 | 28368933 | 0.84 | -0.27 | 1.94 | 1.79 | 0.96 | 3.34 |
| Tachyarrhythmia | 10 | 109632 | 2352 | 28367969 | 0.13 | -0.98 | 1.24 | 1.09 | 0.59 | 2.04 |
| Vasoplegia syndrome | 10 | 109632 | 976 | 28369345 | 1.29 | 0.18 | 2.40 | 2.46 | 1.32 | 4.58 |
| Anaphylactoid reaction | 9 | 109633 | 1560 | 28368761 | 0.54 | -0.64 | 1.71 | 1.46 | 0.76 | 2.80 |
| Extrasystoles | 9 | 109633 | 2608 | 28367713 | -0.15 | -1.33 | 1.02 | 0.90 | 0.47 | 1.73 |
| Haemorrhoids | 9 | 109633 | 8810 | 28361511 | -1.86 | -3.03 | -0.68 | 0.27 | 0.14 | 0.53 |
| Haemothorax | 9 | 109633 | 2348 | 28367973 | -0.01 | -1.19 | 1.16 | 0.99 | 0.52 | 1.91 |
| Increased tendency to bruise | 9 | 109633 | 4641 | 28365680 | -0.95 | -2.13 | 0.22 | 0.52 | 0.27 | 0.99 |
| Ischaemia | 9 | 109633 | 1904 | 28368417 | 0.27 | -0.90 | 1.45 | 1.21 | 0.63 | 2.33 |
| Long qt syndrome | 9 | 109633 | 1981 | 28368340 | 0.22 | -0.95 | 1.39 | 1.16 | 0.61 | 2.24 |
| Lymphoedema | 9 | 109633 | 3148 | 28367173 | -0.41 | -1.59 | 0.76 | 0.75 | 0.39 | 1.44 |
| Oesophageal varices haemorrhage | 9 | 109633 | 2156 | 28368165 | 0.10 | -1.07 | 1.28 | 1.08 | 0.56 | 2.07 |
| Peripheral vascular disorder | 9 | 109633 | 2795 | 28367526 | -0.25 | -1.42 | 0.92 | 0.84 | 0.44 | 1.62 |
| Raynaud’s phenomenon | 9 | 109633 | 2957 | 28367364 | -0.33 | -1.50 | 0.85 | 0.80 | 0.41 | 1.53 |
| Skin haemorrhage | 9 | 109633 | 6342 | 28363979 | -1.39 | -2.57 | -0.22 | 0.38 | 0.20 | 0.73 |
| Subcutaneous haematoma | 9 | 109633 | 1584 | 28368737 | 0.52 | -0.66 | 1.69 | 1.43 | 0.75 | 2.76 |
| Varicose vein | 9 | 109633 | 3374 | 28366947 | -0.51 | -1.68 | 0.66 | 0.70 | 0.36 | 1.35 |
| Vasculitic rash | 9 | 109633 | 862 | 28369459 | 1.30 | 0.13 | 2.48 | 2.48 | 1.29 | 4.78 |
| Ventricular arrhythmia | 9 | 109633 | 2609 | 28367712 | -0.16 | -1.33 | 1.02 | 0.90 | 0.47 | 1.73 |
| Wound haemorrhage | 9 | 109633 | 3871 | 28366450 | -0.70 | -1.87 | 0.47 | 0.61 | 0.32 | 1.18 |
| Atrial flutter | 8 | 109634 | 4739 | 28365582 | -1.14 | -2.40 | 0.11 | 0.45 | 0.23 | 0.90 |
| Cardiac failure chronic | 8 | 109634 | 2081 | 28368240 | -0.01 | -1.26 | 1.25 | 1.00 | 0.50 | 1.99 |
| Enterocolitis haemorrhagic | 8 | 109634 | 967 | 28369354 | 1.00 | -0.26 | 2.25 | 2.01 | 1.00 | 4.02 |
| Hypoperfusion | 8 | 109634 | 309 | 28370012 | 2.30 | 1.05 | 3.56 | 5.02 | 2.49 | 10.12 |
| Infarction | 8 | 109634 | 3371 | 28366950 | -0.67 | -1.92 | 0.59 | 0.63 | 0.31 | 1.26 |
| Left ventricular dysfunction | 8 | 109634 | 4495 | 28365826 | -1.07 | -2.32 | 0.19 | 0.48 | 0.24 | 0.95 |
| Peripheral artery thrombosis | 8 | 109634 | 1182 | 28369139 | 0.74 | -0.51 | 2.00 | 1.68 | 0.84 | 3.36 |
| Systemic inflammatory response syndrome | 8 | 109634 | 5249 | 28365072 | -1.29 | -2.54 | -0.03 | 0.41 | 0.20 | 0.82 |
| Thrombophlebitis | 8 | 109634 | 2945 | 28367376 | -0.48 | -1.74 | 0.77 | 0.72 | 0.36 | 1.43 |
| Thrombophlebitis superficial | 8 | 109634 | 3338 | 28366983 | -0.65 | -1.91 | 0.60 | 0.63 | 0.32 | 1.27 |
| Varices oesophageal | 8 | 109634 | 2036 | 28368285 | 0.02 | -1.23 | 1.28 | 1.02 | 0.51 | 2.03 |
| Aortic dissection | 7 | 109635 | 1756 | 28368565 | 0.04 | -1.31 | 1.40 | 1.03 | 0.49 | 2.16 |
| Axillary vein thrombosis | 7 | 109635 | 125 | 28370196 | 2.90 | 1.54 | 4.25 | 7.63 | 3.56 | 16.33 |
| Brain stem haemorrhage | 7 | 109635 | 784 | 28369537 | 1.08 | -0.27 | 2.44 | 2.12 | 1.01 | 4.47 |
| Cardiac flutter | 7 | 109635 | 2557 | 28367764 | -0.47 | -1.82 | 0.89 | 0.72 | 0.34 | 1.52 |
| Conjunctival haemorrhage | 7 | 109635 | 1772 | 28368549 | 0.03 | -1.33 | 1.38 | 1.02 | 0.49 | 2.14 |
| Duodenal ulcer haemorrhage | 7 | 109635 | 2417 | 28367904 | -0.39 | -1.75 | 0.96 | 0.76 | 0.36 | 1.60 |
| Haemorrhagic arteriovenous malformation | 7 | 109635 | 2829 | 28367492 | -0.61 | -1.96 | 0.75 | 0.66 | 0.31 | 1.38 |
| Haemorrhagic stroke | 7 | 109635 | 10151 | 28360170 | -2.40 | -3.76 | -1.05 | 0.19 | 0.09 | 0.40 |
| Hyperaemia | 7 | 109635 | 609 | 28369712 | 1.39 | 0.03 | 2.74 | 2.63 | 1.25 | 5.54 |
| Intestinal haemorrhage | 7 | 109635 | 3034 | 28367287 | -0.70 | -2.06 | 0.65 | 0.61 | 0.29 | 1.29 |
| Intracranial aneurysm | 7 | 109635 | 2077 | 28368244 | -0.18 | -1.54 | 1.17 | 0.88 | 0.42 | 1.85 |
| Intracranial haematoma | 7 | 109635 | 493 | 28369828 | 1.63 | 0.27 | 2.98 | 3.12 | 1.48 | 6.58 |
| Localised oedema | 7 | 109635 | 1827 | 28368494 | -0.01 | -1.37 | 1.34 | 0.99 | 0.47 | 2.08 |
| Mallory-weiss syndrome | 7 | 109635 | 935 | 28369386 | 0.86 | -0.49 | 2.22 | 1.82 | 0.87 | 3.84 |
| Petechiae | 7 | 109635 | 4892 | 28365429 | -1.37 | -2.72 | -0.01 | 0.39 | 0.18 | 0.81 |
| Pulseless electrical activity | 7 | 109635 | 2383 | 28367938 | -0.37 | -1.73 | 0.98 | 0.77 | 0.37 | 1.62 |
| Renal haemorrhage | 7 | 109635 | 2555 | 28367766 | -0.47 | -1.82 | 0.89 | 0.72 | 0.34 | 1.52 |
| Temporal arteritis | 7 | 109635 | 1272 | 28369049 | 0.47 | -0.89 | 1.82 | 1.38 | 0.66 | 2.91 |
| Venous thrombosis | 7 | 109635 | 3561 | 28366760 | -0.92 | -2.28 | 0.43 | 0.53 | 0.25 | 1.10 |
| Angina unstable | 6 | 109636 | 4064 | 28366257 | -1.31 | -2.80 | 0.17 | 0.40 | 0.18 | 0.89 |
| Ascites | 6 | 109636 | 10761 | 28359560 | -2.69 | -4.17 | -1.21 | 0.15 | 0.07 | 0.34 |
| Atrioventricular block | 6 | 109636 | 2964 | 28367357 | -0.88 | -2.36 | 0.61 | 0.54 | 0.24 | 1.21 |
| Atrioventricular block first degree | 6 | 109636 | 1610 | 28368711 | -0.05 | -1.53 | 1.44 | 0.97 | 0.43 | 2.16 |
| Blood pressure fluctuation | 6 | 109636 | 8371 | 28361950 | -2.33 | -3.82 | -0.85 | 0.20 | 0.09 | 0.44 |
| Cardiorenal syndrome | 6 | 109636 | 239 | 28370082 | 2.17 | 0.69 | 3.65 | 4.57 | 2.03 | 10.27 |
| Diverticulum intestinal haemorrhagic | 6 | 109636 | 3246 | 28367075 | -1.00 | -2.49 | 0.48 | 0.50 | 0.22 | 1.11 |
| Essential hypertension | 6 | 109636 | 884 | 28369437 | 0.73 | -0.76 | 2.21 | 1.66 | 0.74 | 3.70 |
| Gastrointestinal necrosis | 6 | 109636 | 1324 | 28368997 | 0.21 | -1.27 | 1.69 | 1.16 | 0.52 | 2.58 |
| Lymphangiosis carcinomatosa | 6 | 109636 | 551 | 28369770 | 1.30 | -0.19 | 2.78 | 2.47 | 1.11 | 5.53 |
| Papilloedema | 6 | 109636 | 2145 | 28368176 | -0.43 | -1.92 | 1.05 | 0.74 | 0.33 | 1.65 |
| Peripheral ischaemia | 6 | 109636 | 3054 | 28367267 | -0.92 | -2.40 | 0.57 | 0.53 | 0.24 | 1.18 |
| Phlebitis | 6 | 109636 | 2831 | 28367490 | -0.81 | -2.30 | 0.67 | 0.57 | 0.26 | 1.27 |
| Pleuropericarditis | 6 | 109636 | 213 | 28370108 | 2.27 | 0.79 | 3.76 | 4.91 | 2.18 | 11.06 |
| Poor venous access | 6 | 109636 | 3199 | 28367122 | -0.98 | -2.47 | 0.50 | 0.51 | 0.23 | 1.13 |
| Post procedural haemorrhage | 6 | 109636 | 7354 | 28362967 | -2.15 | -3.63 | -0.67 | 0.22 | 0.10 | 0.50 |
| Renal artery arteriosclerosis | 6 | 109636 | 123 | 28370198 | 2.71 | 1.22 | 4.19 | 6.66 | 2.94 | 15.12 |
| Spinal cord haemorrhage | 6 | 109636 | 410 | 28369911 | 1.63 | 0.15 | 3.11 | 3.12 | 1.39 | 6.98 |
| Splenic infarction | 6 | 109636 | 1154 | 28369167 | 0.39 | -1.09 | 1.87 | 1.31 | 0.59 | 2.92 |
| Takayasu’s arteritis | 6 | 109636 | 428 | 28369893 | 1.58 | 0.10 | 3.07 | 3.02 | 1.35 | 6.75 |
| Vascular device infection | 6 | 109636 | 1933 | 28368388 | -0.29 | -1.78 | 1.19 | 0.82 | 0.37 | 1.82 |
| White matter lesion | 6 | 109636 | 899 | 28369422 | 0.71 | -0.78 | 2.19 | 1.64 | 0.73 | 3.65 |
| Abdominal wall haematoma | 5 | 109637 | 1547 | 28368774 | -0.24 | -1.89 | 1.42 | 0.85 | 0.35 | 2.04 |
| Aortic aneurysm | 5 | 109637 | 3168 | 28367153 | -1.21 | -2.86 | 0.45 | 0.43 | 0.18 | 1.04 |
| Basilar artery occlusion | 5 | 109637 | 83 | 28370238 | 2.71 | 1.06 | 4.37 | 6.70 | 2.72 | 16.52 |
| Cardiac death | 5 | 109637 | 622 | 28369699 | 0.92 | -0.74 | 2.57 | 1.89 | 0.79 | 4.57 |
| Cardiotoxicity | 5 | 109637 | 3447 | 28366874 | -1.33 | -2.98 | 0.33 | 0.40 | 0.17 | 0.96 |
| Cerebellar infarction | 5 | 109637 | 962 | 28369359 | 0.38 | -1.27 | 2.04 | 1.30 | 0.54 | 3.14 |
| Gangrene | 5 | 109637 | 2766 | 28367555 | -1.02 | -2.68 | 0.63 | 0.49 | 0.20 | 1.18 |
| Genital haemorrhage | 5 | 109637 | 4565 | 28365756 | -1.72 | -3.37 | -0.06 | 0.30 | 0.13 | 0.73 |
| Haemorrhagic infarction | 5 | 109637 | 353 | 28369968 | 1.55 | -0.10 | 3.20 | 2.95 | 1.22 | 7.13 |
| Hypovolaemic shock | 5 | 109637 | 2722 | 28367599 | -1.00 | -2.65 | 0.65 | 0.50 | 0.21 | 1.20 |
| Hypoxic-ischaemic encephalopathy | 5 | 109637 | 1225 | 28369096 | 0.07 | -1.58 | 1.73 | 1.05 | 0.44 | 2.53 |
| Intermittent claudication | 5 | 109637 | 643 | 28369678 | 0.88 | -0.78 | 2.53 | 1.84 | 0.76 | 4.44 |
| Intra-abdominal haematoma | 5 | 109637 | 971 | 28369350 | 0.37 | -1.29 | 2.02 | 1.29 | 0.54 | 3.11 |
| Intra-abdominal haemorrhage | 5 | 109637 | 1467 | 28368854 | -0.17 | -1.82 | 1.49 | 0.89 | 0.37 | 2.15 |
| Intracranial tumour haemorrhage | 5 | 109637 | 325 | 28369996 | 1.64 | -0.02 | 3.29 | 3.13 | 1.29 | 7.58 |
| Ischaemic cardiomyopathy | 5 | 109637 | 1496 | 28368825 | -0.19 | -1.85 | 1.46 | 0.88 | 0.36 | 2.11 |
| Mesenteric artery embolism | 5 | 109637 | 131 | 28370190 | 2.43 | 0.77 | 4.08 | 5.47 | 2.24 | 13.35 |
| Optic ischaemic neuropathy | 5 | 109637 | 1217 | 28369104 | 0.08 | -1.58 | 1.73 | 1.06 | 0.44 | 2.54 |
| Orthopnoea | 5 | 109637 | 1451 | 28368870 | -0.15 | -1.81 | 1.50 | 0.90 | 0.37 | 2.17 |
| Peripheral artery occlusion | 5 | 109637 | 1332 | 28368989 | -0.04 | -1.69 | 1.62 | 0.97 | 0.40 | 2.34 |
| Portal vein thrombosis | 5 | 109637 | 1996 | 28368325 | -0.58 | -2.23 | 1.08 | 0.67 | 0.28 | 1.61 |
| Premature separation of placenta | 5 | 109637 | 819 | 28369502 | 0.58 | -1.07 | 2.24 | 1.50 | 0.62 | 3.62 |
| Prostatic haemorrhage | 5 | 109637 | 349 | 28369972 | 1.56 | -0.09 | 3.22 | 2.98 | 1.23 | 7.19 |
| Rectal ulcer haemorrhage | 5 | 109637 | 319 | 28370002 | 1.65 | 0.00 | 3.31 | 3.17 | 1.31 | 7.68 |
| Retinal vein occlusion | 5 | 109637 | 1901 | 28368420 | -0.51 | -2.17 | 1.14 | 0.70 | 0.29 | 1.69 |
| Retinal vein thrombosis | 5 | 109637 | 388 | 28369933 | 1.45 | -0.20 | 3.10 | 2.75 | 1.14 | 6.65 |
| Retroperitoneal haematoma | 5 | 109637 | 1895 | 28368426 | -0.51 | -2.16 | 1.15 | 0.70 | 0.29 | 1.69 |
| Stent-graft endoleak | 5 | 109637 | 79 | 28370242 | 2.74 | 1.09 | 4.39 | 6.83 | 2.77 | 16.86 |
| Thrombosis in device | 5 | 109637 | 2181 | 28368140 | -0.70 | -2.35 | 0.96 | 0.62 | 0.26 | 1.48 |
| Urinary bladder haemorrhage | 5 | 109637 | 2370 | 28367951 | -0.81 | -2.46 | 0.84 | 0.57 | 0.24 | 1.37 |
| Uterine haemorrhage | 5 | 109637 | 3024 | 28367297 | -1.14 | -2.80 | 0.51 | 0.45 | 0.19 | 1.09 |
| Vascular rupture | 5 | 109637 | 1050 | 28369271 | 0.27 | -1.38 | 1.92 | 1.21 | 0.50 | 2.91 |
| Vertebral artery occlusion | 5 | 109637 | 306 | 28370015 | 1.70 | 0.04 | 3.35 | 3.27 | 1.35 | 7.91 |
| Vitreous haemorrhage | 5 | 109637 | 2661 | 28367660 | -0.97 | -2.62 | 0.69 | 0.51 | 0.21 | 1.23 |
| Aortic valve incompetence | 4 | 109638 | 978 | 28369343 | 0.07 | -1.83 | 1.97 | 1.05 | 0.39 | 2.81 |
| Arteriosclerosis | 4 | 109638 | 2671 | 28367650 | -1.26 | -3.16 | 0.64 | 0.42 | 0.16 | 1.11 |
| Arteriosclerosis coronary artery | 4 | 109638 | 1765 | 28368556 | -0.70 | -2.60 | 1.20 | 0.61 | 0.23 | 1.64 |
| Atrial septal defect acquired | 4 | 109638 | 109 | 28370212 | 2.27 | 0.37 | 4.17 | 4.88 | 1.80 | 13.25 |
| Bundle branch block | 4 | 109638 | 290 | 28370031 | 1.46 | -0.44 | 3.36 | 2.78 | 1.04 | 7.45 |
| Cerebral artery embolism | 4 | 109638 | 607 | 28369714 | 0.66 | -1.24 | 2.56 | 1.58 | 0.59 | 4.23 |
| Cerebral haematoma | 4 | 109638 | 2103 | 28368218 | -0.94 | -2.84 | 0.96 | 0.52 | 0.20 | 1.39 |
| Cerebral ischaemia | 4 | 109638 | 2682 | 28367639 | -1.27 | -3.17 | 0.63 | 0.41 | 0.16 | 1.10 |
| Cutaneous vasculitis | 4 | 109638 | 710 | 28369611 | 0.47 | -1.43 | 2.37 | 1.39 | 0.52 | 3.71 |
| Gastritis haemorrhagic | 4 | 109638 | 1473 | 28368848 | -0.46 | -2.36 | 1.44 | 0.73 | 0.27 | 1.94 |
| Haemorrhagic cerebral infarction | 4 | 109638 | 584 | 28369737 | 0.70 | -1.20 | 2.60 | 1.63 | 0.61 | 4.36 |
| Injection site haematoma | 4 | 109638 | 1429 | 28368892 | -0.42 | -2.32 | 1.48 | 0.75 | 0.28 | 1.99 |
| Ischaemic cerebral infarction | 4 | 109638 | 1052 | 28369269 | -0.02 | -1.92 | 1.88 | 0.99 | 0.37 | 2.63 |
| Migraine with aura | 4 | 109638 | 1326 | 28368995 | -0.32 | -2.22 | 1.58 | 0.80 | 0.30 | 2.13 |
| Necrosis | 4 | 109638 | 2071 | 28368250 | -0.92 | -2.82 | 0.98 | 0.53 | 0.20 | 1.41 |
| Nodal arrhythmia | 4 | 109638 | 383 | 28369938 | 1.18 | -0.72 | 3.08 | 2.27 | 0.85 | 6.09 |
| Oesophageal haemorrhage | 4 | 109638 | 899 | 28369422 | 0.18 | -1.72 | 2.08 | 1.13 | 0.42 | 3.02 |
| Peripheral embolism | 4 | 109638 | 724 | 28369597 | 0.45 | -1.45 | 2.35 | 1.36 | 0.51 | 3.65 |
| Poor peripheral circulation | 4 | 109638 | 2271 | 28368050 | -1.04 | -2.94 | 0.86 | 0.49 | 0.18 | 1.29 |
| Pulmonary artery atresia | 4 | 109638 | 62 | 28370259 | 2.58 | 0.68 | 4.48 | 6.08 | 2.21 | 16.72 |
| Renal ischaemia | 4 | 109638 | 277 | 28370044 | 1.51 | -0.39 | 3.41 | 2.87 | 1.07 | 7.69 |
| Retinal vascular occlusion | 4 | 109638 | 420 | 28369901 | 1.08 | -0.82 | 2.98 | 2.12 | 0.79 | 5.67 |
| Retroperitoneal haemorrhage | 4 | 109638 | 1953 | 28368368 | -0.84 | -2.74 | 1.06 | 0.56 | 0.21 | 1.49 |
| Small intestinal ulcer haemorrhage | 4 | 109638 | 172 | 28370149 | 1.93 | 0.03 | 3.83 | 3.86 | 1.43 | 10.41 |
| Splenic haematoma | 4 | 109638 | 294 | 28370027 | 1.45 | -0.45 | 3.35 | 2.75 | 1.03 | 7.38 |
| Telangiectasia | 4 | 109638 | 503 | 28369818 | 0.88 | -1.02 | 2.78 | 1.84 | 0.69 | 4.93 |
| Vascular injury | 4 | 109638 | 976 | 28369345 | 0.07 | -1.83 | 1.97 | 1.05 | 0.39 | 2.81 |
| Vasodilatation | 4 | 109638 | 1983 | 28368338 | -0.86 | -2.76 | 1.04 | 0.55 | 0.21 | 1.47 |
| Vena cava thrombosis | 4 | 109638 | 1505 | 28368816 | -0.49 | -2.39 | 1.41 | 0.71 | 0.27 | 1.90 |
| Angiopathy | 3 | 109639 | 1727 | 28368594 | -1.03 | -3.32 | 1.26 | 0.49 | 0.16 | 1.51 |
| Anti-neutrophil cytoplasmic antibody positive vasculitis | 3 | 109639 | 781 | 28369540 | -0.01 | -2.30 | 2.28 | 0.99 | 0.32 | 3.09 |
| Aortic aneurysm rupture | 3 | 109639 | 729 | 28369592 | 0.08 | -2.21 | 2.37 | 1.06 | 0.34 | 3.28 |
| Aortic stenosis | 3 | 109639 | 1401 | 28368920 | -0.75 | -3.04 | 1.54 | 0.59 | 0.19 | 1.84 |
| Arterial stiffness | 3 | 109639 | 4 | 28370317 | 2.73 | 0.44 | 5.02 | 6.79 | 1.52 | 30.34 |
| Arteriospasm coronary | 3 | 109639 | 1642 | 28368679 | -0.97 | -3.26 | 1.33 | 0.51 | 0.16 | 1.59 |
| Atrial tachycardia | 3 | 109639 | 640 | 28369681 | 0.23 | -2.06 | 2.52 | 1.18 | 0.38 | 3.66 |
| Atrial thrombosis | 3 | 109639 | 1393 | 28368928 | -0.75 | -3.04 | 1.54 | 0.59 | 0.19 | 1.85 |
| Atrioventricular block second degree | 3 | 109639 | 1450 | 28368871 | -0.80 | -3.09 | 1.49 | 0.57 | 0.18 | 1.78 |
| Bradyarrhythmia | 3 | 109639 | 655 | 28369666 | 0.21 | -2.08 | 2.50 | 1.15 | 0.37 | 3.59 |
| Cardiac amyloidosis | 3 | 109639 | 291 | 28370030 | 1.10 | -1.19 | 3.39 | 2.15 | 0.69 | 6.72 |
| Cardiac valve disease | 3 | 109639 | 2151 | 28368170 | -1.33 | -3.62 | 0.96 | 0.40 | 0.13 | 1.23 |
| Cerebellar haemorrhage | 3 | 109639 | 1405 | 28368916 | -0.76 | -3.05 | 1.53 | 0.59 | 0.19 | 1.83 |
| Congestive cardiomyopathy | 3 | 109639 | 2629 | 28367692 | -1.60 | -3.89 | 0.69 | 0.33 | 0.11 | 1.02 |
| Coronary artery occlusion | 3 | 109639 | 6038 | 28364283 | -2.76 | -5.05 | -0.47 | 0.15 | 0.05 | 0.46 |
| Coronary artery thrombosis | 3 | 109639 | 699 | 28369622 | 0.13 | -2.16 | 2.42 | 1.09 | 0.35 | 3.40 |
| Diastolic dysfunction | 3 | 109639 | 852 | 28369469 | -0.12 | -2.41 | 2.17 | 0.92 | 0.30 | 2.87 |
| Embolism arterial | 3 | 109639 | 808 | 28369513 | -0.05 | -2.34 | 2.24 | 0.97 | 0.31 | 3.00 |
| Extradural haematoma | 3 | 109639 | 684 | 28369637 | 0.15 | -2.14 | 2.44 | 1.11 | 0.36 | 3.46 |
| Eye haemorrhage | 3 | 109639 | 6057 | 28364264 | -2.77 | -5.06 | -0.48 | 0.15 | 0.05 | 0.45 |
| Fat embolism | 3 | 109639 | 125 | 28370196 | 1.82 | -0.47 | 4.11 | 3.56 | 1.13 | 11.19 |
| Gastric varices haemorrhage | 3 | 109639 | 171 | 28370150 | 1.58 | -0.71 | 3.87 | 3.02 | 0.96 | 9.44 |
| Gastrointestinal polyp haemorrhage | 3 | 109639 | 763 | 28369558 | 0.02 | -2.27 | 2.31 | 1.01 | 0.33 | 3.15 |
| Gastrointestinal vascular malformation haemorrhagic | 3 | 109639 | 373 | 28369948 | 0.85 | -1.44 | 3.14 | 1.80 | 0.58 | 5.62 |
| Haemorrhagic diathesis | 3 | 109639 | 1804 | 28368517 | -1.09 | -3.38 | 1.20 | 0.47 | 0.15 | 1.45 |
| Heart transplant rejection | 3 | 109639 | 454 | 28369867 | 0.63 | -1.66 | 2.92 | 1.55 | 0.50 | 4.83 |
| Henoch-schonlein purpura | 3 | 109639 | 907 | 28369414 | -0.19 | -2.48 | 2.10 | 0.87 | 0.28 | 2.71 |
| Heparin-induced thrombocytopenia | 3 | 109639 | 1484 | 28368837 | -0.83 | -3.12 | 1.46 | 0.56 | 0.18 | 1.74 |
| Hypertensive emergency | 3 | 109639 | 786 | 28369535 | -0.02 | -2.31 | 2.27 | 0.99 | 0.32 | 3.07 |
| Hypertensive heart disease | 3 | 109639 | 907 | 28369414 | -0.19 | -2.48 | 2.10 | 0.87 | 0.28 | 2.71 |
| Intracardiac thrombus | 3 | 109639 | 1698 | 28368623 | -1.01 | -3.30 | 1.28 | 0.50 | 0.16 | 1.54 |
| Intraventricular haemorrhage | 3 | 109639 | 2191 | 28368130 | -1.35 | -3.64 | 0.94 | 0.39 | 0.13 | 1.21 |
| Ischaemic nephropathy | 3 | 109639 | 21 | 28370300 | 2.56 | 0.27 | 4.85 | 6.02 | 1.80 | 20.19 |
| Left ventricular failure | 3 | 109639 | 2428 | 28367893 | -1.49 | -3.78 | 0.80 | 0.35 | 0.11 | 1.10 |
| Microscopic polyangiitis | 3 | 109639 | 159 | 28370162 | 1.64 | -0.65 | 3.93 | 3.14 | 1.00 | 9.84 |
| Mitral valve disease | 3 | 109639 | 935 | 28369386 | -0.23 | -2.52 | 2.06 | 0.85 | 0.27 | 2.64 |
| Myocardial necrosis | 3 | 109639 | 117 | 28370204 | 1.86 | -0.43 | 4.15 | 3.68 | 1.17 | 11.56 |
| Myoglobinuria | 3 | 109639 | 157 | 28370164 | 1.65 | -0.64 | 3.94 | 3.16 | 1.01 | 9.91 |
| Pelvic venous thrombosis | 3 | 109639 | 578 | 28369743 | 0.35 | -1.94 | 2.65 | 1.28 | 0.41 | 3.98 |
| Peptic ulcer haemorrhage | 3 | 109639 | 745 | 28369576 | 0.05 | -2.24 | 2.34 | 1.04 | 0.33 | 3.22 |
| Peripheral artery stenosis | 3 | 109639 | 634 | 28369687 | 0.25 | -2.04 | 2.54 | 1.19 | 0.38 | 3.69 |
| Pituitary haemorrhage | 3 | 109639 | 258 | 28370063 | 1.22 | -1.07 | 3.51 | 2.34 | 0.75 | 7.30 |
| Portal hypertension | 3 | 109639 | 1392 | 28368929 | -0.75 | -3.04 | 1.54 | 0.60 | 0.19 | 1.85 |
| Post procedural haematoma | 3 | 109639 | 1610 | 28368711 | -0.94 | -3.23 | 1.35 | 0.52 | 0.17 | 1.62 |
| Pre-eclampsia | 3 | 109639 | 2332 | 28367989 | -1.44 | -3.73 | 0.85 | 0.37 | 0.12 | 1.14 |
| Pulmonary artery thrombosis | 3 | 109639 | 726 | 28369595 | 0.08 | -2.21 | 2.37 | 1.06 | 0.34 | 3.29 |
| Renal cortical necrosis | 3 | 109639 | 110 | 28370211 | 1.90 | -0.39 | 4.19 | 3.78 | 1.20 | 11.91 |
| Renal cyst haemorrhage | 3 | 109639 | 270 | 28370051 | 1.17 | -1.12 | 3.46 | 2.27 | 0.73 | 7.08 |
| Retinal artery occlusion | 3 | 109639 | 1456 | 28368865 | -0.81 | -3.10 | 1.48 | 0.57 | 0.18 | 1.77 |
| Retinal oedema | 3 | 109639 | 702 | 28369619 | 0.12 | -2.17 | 2.41 | 1.09 | 0.35 | 3.39 |
| Spider naevus | 3 | 109639 | 180 | 28370141 | 1.54 | -0.75 | 3.83 | 2.93 | 0.94 | 9.16 |
| Spinal cord infarction | 3 | 109639 | 152 | 28370169 | 1.67 | -0.62 | 3.96 | 3.22 | 1.03 | 10.09 |
| Superior vena cava syndrome | 3 | 109639 | 522 | 28369799 | 0.47 | -1.82 | 2.76 | 1.39 | 0.45 | 4.33 |
| Supraventricular extrasystoles | 3 | 109639 | 1659 | 28368662 | -0.98 | -3.27 | 1.31 | 0.51 | 0.16 | 1.57 |
| Systemic scleroderma | 3 | 109639 | 432 | 28369889 | 0.69 | -1.60 | 2.98 | 1.61 | 0.52 | 5.02 |
| Systolic hypertension | 3 | 109639 | 374 | 28369947 | 0.84 | -1.45 | 3.13 | 1.80 | 0.58 | 5.60 |
| Thrombotic stroke | 3 | 109639 | 610 | 28369711 | 0.29 | -2.00 | 2.58 | 1.22 | 0.39 | 3.81 |
| Tracheal haemorrhage | 3 | 109639 | 370 | 28369951 | 0.85 | -1.44 | 3.14 | 1.81 | 0.58 | 5.65 |
| Transverse sinus thrombosis | 3 | 109639 | 392 | 28369929 | 0.79 | -1.50 | 3.08 | 1.74 | 0.56 | 5.41 |
| Traumatic haematoma | 3 | 109639 | 1173 | 28369148 | -0.52 | -2.81 | 1.77 | 0.70 | 0.22 | 2.16 |
| Trousseau’s syndrome | 3 | 109639 | 357 | 28369964 | 0.89 | -1.40 | 3.18 | 1.86 | 0.60 | 5.80 |
| Varicocele | 3 | 109639 | 159 | 28370162 | 1.64 | -0.65 | 3.93 | 3.14 | 1.00 | 9.84 |
| Vascular compression | 3 | 109639 | 243 | 28370078 | 1.27 | -1.02 | 3.56 | 2.43 | 0.78 | 7.59 |
| Vascular stent stenosis | 3 | 109639 | 709 | 28369612 | 0.11 | -2.18 | 2.40 | 1.08 | 0.35 | 3.36 |
| Vascular stent thrombosis | 3 | 109639 | 3609 | 28366712 | -2.04 | -4.33 | 0.25 | 0.24 | 0.08 | 0.75 |
| Vena cava embolism | 3 | 109639 | 90 | 28370231 | 2.03 | -0.26 | 4.32 | 4.13 | 1.31 | 13.04 |
| Venoocclusive liver disease | 3 | 109639 | 6286 | 28364035 | -2.82 | -5.11 | -0.53 | 0.14 | 0.05 | 0.44 |

**Table S4.** Disproportionality results of cardiac and vascular toxicities associated with pantoprazole

| PT | a | b | c | d | IC | IC_025_ | IC_975_ | ROR | ROR_025_ | ROR_975_ |
| --- | --- | --- | --- | --- | --- | --- | --- | --- | --- | --- |
| Gastrointestinal haemorrhage | 1035 | 170465 | 117734 | 28190729 | 0.53 | 0.43 | 0.64 | 1.45 | 1.37 | 1.55 |
| Dyspnoea | 1008 | 170492 | 217141 | 28091322 | -0.38 | -0.49 | -0.28 | 0.76 | 0.72 | 0.81 |
| Syncope | 707 | 170793 | 74608 | 28233855 | 0.64 | 0.52 | 0.76 | 1.57 | 1.45 | 1.69 |
| Peripheral swelling | 562 | 170938 | 104029 | 28204434 | -0.16 | -0.30 | -0.02 | 0.89 | 0.82 | 0.97 |
| Pulmonary embolism | 548 | 170952 | 69527 | 28238936 | 0.38 | 0.24 | 0.52 | 1.30 | 1.20 | 1.42 |
| Myocardial infarction | 515 | 170985 | 95815 | 28212648 | -0.17 | -0.32 | -0.03 | 0.89 | 0.81 | 0.97 |
| Dizziness | 513 | 170987 | 172323 | 28136140 | -1.02 | -1.17 | -0.87 | 0.49 | 0.45 | 0.53 |
| Rectal haemorrhage | 484 | 171016 | 47518 | 28260945 | 0.74 | 0.59 | 0.89 | 1.68 | 1.54 | 1.84 |
| Hypertension | 471 | 171029 | 87693 | 28220770 | -0.17 | -0.32 | -0.02 | 0.89 | 0.81 | 0.97 |
| Hypotension | 468 | 171032 | 87000 | 28221463 | -0.17 | -0.32 | -0.02 | 0.89 | 0.81 | 0.97 |
| Thrombosis | 465 | 171035 | 63215 | 28245248 | 0.28 | 0.12 | 0.43 | 1.21 | 1.11 | 1.33 |
| Upper gastrointestinal haemorrhage | 419 | 171081 | 38108 | 28270355 | 0.85 | 0.69 | 1.01 | 1.82 | 1.65 | 2.00 |
| Cerebrovascular accident | 358 | 171142 | 77768 | 28230695 | -0.39 | -0.57 | -0.22 | 0.76 | 0.68 | 0.84 |
| Oedema peripheral | 320 | 171180 | 50075 | 28258388 | 0.08 | -0.11 | 0.26 | 1.05 | 0.94 | 1.18 |
| Tachycardia | 288 | 171212 | 44359 | 28264104 | 0.10 | -0.10 | 0.29 | 1.07 | 0.95 | 1.20 |
| Chest pain | 281 | 171219 | 67730 | 28240733 | -0.54 | -0.74 | -0.35 | 0.68 | 0.61 | 0.77 |
| Septic shock | 278 | 171222 | 38113 | 28270350 | 0.27 | 0.07 | 0.46 | 1.20 | 1.07 | 1.35 |
| Haematochezia | 265 | 171235 | 24204 | 28284259 | 0.84 | 0.64 | 1.05 | 1.81 | 1.60 | 2.04 |
| Palpitations | 260 | 171240 | 52509 | 28255954 | -0.29 | -0.49 | -0.08 | 0.82 | 0.72 | 0.92 |
| Transient ischaemic attack | 219 | 171281 | 30173 | 28278290 | 0.26 | 0.03 | 0.48 | 1.20 | 1.05 | 1.37 |
| Pulmonary oedema | 214 | 171286 | 27569 | 28280894 | 0.35 | 0.13 | 0.58 | 1.28 | 1.12 | 1.47 |
| Haemorrhage | 213 | 171287 | 44478 | 28263985 | -0.34 | -0.56 | -0.11 | 0.79 | 0.69 | 0.90 |
| Melaena | 201 | 171299 | 15110 | 28293353 | 1.12 | 0.89 | 1.35 | 2.19 | 1.91 | 2.52 |
| Atrial fibrillation | 188 | 171312 | 44889 | 28263574 | -0.53 | -0.77 | -0.29 | 0.69 | 0.60 | 0.80 |
| Migraine | 186 | 171314 | 42397 | 28266066 | -0.46 | -0.70 | -0.22 | 0.72 | 0.63 | 0.84 |
| Epistaxis | 180 | 171320 | 39881 | 28268582 | -0.42 | -0.67 | -0.17 | 0.75 | 0.64 | 0.86 |
| Cardiac failure | 178 | 171322 | 38204 | 28270259 | -0.38 | -0.62 | -0.13 | 0.77 | 0.66 | 0.89 |
| Deep vein thrombosis | 161 | 171339 | 33085 | 28275378 | -0.31 | -0.57 | -0.05 | 0.80 | 0.69 | 0.94 |
| Internal haemorrhage | 157 | 171343 | 19549 | 28288914 | 0.40 | 0.14 | 0.67 | 1.32 | 1.13 | 1.55 |
| Haematuria | 156 | 171344 | 23110 | 28285353 | 0.15 | -0.11 | 0.42 | 1.11 | 0.95 | 1.30 |
| Lower gastrointestinal haemorrhage | 156 | 171344 | 17760 | 28290703 | 0.53 | 0.26 | 0.80 | 1.45 | 1.24 | 1.70 |
| Subdural haematoma | 148 | 171352 | 20540 | 28287923 | 0.25 | -0.02 | 0.52 | 1.19 | 1.01 | 1.40 |
| Cardiac failure congestive | 139 | 171361 | 43039 | 28265424 | -0.90 | -1.18 | -0.62 | 0.53 | 0.45 | 0.63 |
| Renal haemangioma | 139 | 171361 | 1943 | 28306520 | 3.42 | 3.14 | 3.70 | 11.38 | 9.58 | 13.51 |
| Ischaemic stroke | 126 | 171374 | 18110 | 28290353 | 0.20 | -0.10 | 0.49 | 1.15 | 0.96 | 1.37 |
| Hot flush | 123 | 171377 | 27906 | 28280557 | -0.45 | -0.75 | -0.16 | 0.73 | 0.61 | 0.87 |
| Haematemesis | 122 | 171378 | 12310 | 28296153 | 0.70 | 0.40 | 1.00 | 1.63 | 1.37 | 1.95 |
| Cerebral haemorrhage | 116 | 171384 | 23932 | 28284531 | -0.32 | -0.63 | -0.01 | 0.80 | 0.67 | 0.96 |
| Presyncope | 113 | 171387 | 15048 | 28293415 | 0.31 | -0.01 | 0.62 | 1.24 | 1.03 | 1.49 |
| Cardiac disorder | 109 | 171391 | 32274 | 28276189 | -0.84 | -1.15 | -0.52 | 0.56 | 0.46 | 0.67 |
| Haematoma | 107 | 171393 | 14646 | 28293817 | 0.27 | -0.05 | 0.59 | 1.20 | 1.00 | 1.46 |
| Shock haemorrhagic | 106 | 171394 | 9072 | 28299391 | 0.93 | 0.61 | 1.26 | 1.92 | 1.59 | 2.33 |
| Anaphylactic reaction | 103 | 171397 | 25585 | 28282878 | -0.58 | -0.91 | -0.26 | 0.67 | 0.55 | 0.81 |
| Cardiac arrest | 100 | 171400 | 31312 | 28277151 | -0.92 | -1.25 | -0.58 | 0.53 | 0.43 | 0.64 |
| Haemoptysis | 100 | 171400 | 18381 | 28290082 | -0.15 | -0.49 | 0.18 | 0.90 | 0.74 | 1.09 |
| Pulmonary hypertension | 98 | 171402 | 14314 | 28294149 | 0.17 | -0.16 | 0.51 | 1.13 | 0.93 | 1.38 |
| Dyspnoea exertional | 93 | 171407 | 14950 | 28293513 | 0.04 | -0.31 | 0.38 | 1.03 | 0.84 | 1.26 |
| Ulcer haemorrhage | 93 | 171407 | 5858 | 28302605 | 1.36 | 1.02 | 1.71 | 2.60 | 2.12 | 3.19 |
| Contusion | 91 | 171409 | 35247 | 28273216 | -1.22 | -1.57 | -0.87 | 0.43 | 0.35 | 0.52 |
| Osteonecrosis of jaw | 90 | 171410 | 26533 | 28281930 | -0.83 | -1.18 | -0.48 | 0.56 | 0.46 | 0.69 |
| Ventricular tachycardia | 89 | 171411 | 12714 | 28295749 | 0.21 | -0.15 | 0.56 | 1.15 | 0.94 | 1.42 |
| Vaginal haemorrhage | 88 | 171412 | 26919 | 28281544 | -0.88 | -1.24 | -0.53 | 0.54 | 0.44 | 0.67 |
| Subarachnoid haemorrhage | 87 | 171413 | 10086 | 28298377 | 0.50 | 0.15 | 0.86 | 1.42 | 1.15 | 1.75 |
| Flushing | 85 | 171415 | 30280 | 28278183 | -1.10 | -1.46 | -0.74 | 0.46 | 0.38 | 0.57 |
| Pallor | 83 | 171417 | 8709 | 28299754 | 0.64 | 0.28 | 1.01 | 1.57 | 1.26 | 1.95 |
| Orthostatic hypotension | 82 | 171418 | 12442 | 28296021 | 0.12 | -0.25 | 0.49 | 1.09 | 0.88 | 1.35 |
| Acute myocardial infarction | 81 | 171419 | 21851 | 28286612 | -0.70 | -1.07 | -0.33 | 0.61 | 0.49 | 0.76 |
| Pulmonary arterial hypertension | 80 | 171420 | 13812 | 28294651 | -0.06 | -0.44 | 0.31 | 0.96 | 0.77 | 1.19 |
| Skin ulcer | 80 | 171420 | 18233 | 28290230 | -0.46 | -0.83 | -0.09 | 0.73 | 0.58 | 0.90 |
| Pericardial effusion | 71 | 171429 | 12215 | 28296248 | -0.06 | -0.45 | 0.34 | 0.96 | 0.76 | 1.21 |
| Bradycardia | 70 | 171430 | 19747 | 28288716 | -0.77 | -1.16 | -0.37 | 0.59 | 0.46 | 0.74 |
| Supraventricular tachycardia | 69 | 171431 | 7778 | 28300685 | 0.54 | 0.14 | 0.94 | 1.46 | 1.15 | 1.85 |
| Vasculitis | 69 | 171431 | 7292 | 28301171 | 0.63 | 0.23 | 1.03 | 1.56 | 1.23 | 1.97 |
| Haemorrhagic stroke | 67 | 171433 | 10091 | 28298372 | 0.13 | -0.28 | 0.54 | 1.10 | 0.86 | 1.39 |
| Anaphylactic shock | 66 | 171434 | 12985 | 28295478 | -0.25 | -0.66 | 0.16 | 0.84 | 0.66 | 1.07 |
| Hypertensive crisis | 64 | 171436 | 8018 | 28300445 | 0.39 | -0.02 | 0.81 | 1.31 | 1.03 | 1.68 |
| Ventricular septal defect | 64 | 171436 | 4552 | 28303911 | 1.19 | 0.77 | 1.61 | 2.30 | 1.80 | 2.94 |
| Coronary artery disease | 63 | 171437 | 13738 | 28294725 | -0.40 | -0.82 | 0.02 | 0.76 | 0.59 | 0.97 |
| Haemorrhoids | 63 | 171437 | 8756 | 28299707 | 0.24 | -0.18 | 0.66 | 1.19 | 0.93 | 1.52 |
| Haemorrhage intracranial | 62 | 171438 | 13774 | 28294689 | -0.42 | -0.85 | 0.00 | 0.74 | 0.58 | 0.96 |
| Arrhythmia | 61 | 171439 | 17057 | 28291406 | -0.75 | -1.18 | -0.33 | 0.59 | 0.46 | 0.76 |
| Chest discomfort | 60 | 171440 | 32132 | 28276331 | -1.68 | -2.11 | -1.25 | 0.31 | 0.24 | 0.40 |
| Sudden death | 58 | 171442 | 11040 | 28297423 | -0.20 | -0.64 | 0.24 | 0.87 | 0.67 | 1.12 |
| Angina pectoris | 54 | 171446 | 12416 | 28296047 | -0.47 | -0.93 | -0.02 | 0.72 | 0.55 | 0.94 |
| Injection site bruising | 54 | 171446 | 29516 | 28278947 | -1.71 | -2.17 | -1.26 | 0.30 | 0.23 | 0.40 |
| Shock | 54 | 171446 | 10729 | 28297734 | -0.26 | -0.72 | 0.19 | 0.83 | 0.64 | 1.09 |
| Gastric haemorrhage | 52 | 171448 | 5974 | 28302489 | 0.51 | 0.05 | 0.98 | 1.43 | 1.09 | 1.88 |
| Sinus bradycardia | 52 | 171448 | 6180 | 28302283 | 0.47 | 0.00 | 0.93 | 1.38 | 1.05 | 1.82 |
| Pericardial haemorrhage | 51 | 171449 | 3122 | 28305341 | 1.39 | 0.93 | 1.86 | 2.65 | 2.01 | 3.50 |
| Post procedural haemorrhage | 50 | 171450 | 7310 | 28301153 | 0.17 | -0.30 | 0.64 | 1.13 | 0.85 | 1.49 |
| Systemic inflammatory response syndrome | 50 | 171450 | 5207 | 28303256 | 0.65 | 0.18 | 1.12 | 1.58 | 1.19 | 2.08 |
| Ventricular extrasystoles | 50 | 171450 | 6283 | 28302180 | 0.39 | -0.09 | 0.86 | 1.31 | 0.99 | 1.73 |
| Ventricular fibrillation | 49 | 171451 | 8391 | 28300072 | -0.05 | -0.53 | 0.43 | 0.96 | 0.73 | 1.28 |
| Diarrhoea haemorrhagic | 48 | 171452 | 3863 | 28304600 | 1.01 | 0.53 | 1.49 | 2.03 | 1.53 | 2.70 |
| Right ventricular failure | 48 | 171452 | 7378 | 28301085 | 0.10 | -0.38 | 0.58 | 1.07 | 0.81 | 1.43 |
| Sinus tachycardia | 48 | 171452 | 6640 | 28301823 | 0.25 | -0.23 | 0.73 | 1.19 | 0.90 | 1.58 |
| Cerebral infarction | 47 | 171453 | 16120 | 28292343 | -1.04 | -1.53 | -0.56 | 0.48 | 0.36 | 0.64 |
| Pulmonary thrombosis | 47 | 171453 | 7458 | 28301005 | 0.06 | -0.43 | 0.54 | 1.04 | 0.78 | 1.39 |
| Hypersensitivity vasculitis | 46 | 171454 | 3632 | 28304831 | 1.04 | 0.54 | 1.53 | 2.07 | 1.55 | 2.76 |
| Wound haemorrhage | 45 | 171455 | 3835 | 28304628 | 0.93 | 0.43 | 1.43 | 1.92 | 1.43 | 2.57 |
| Cardio-respiratory arrest | 43 | 171457 | 17186 | 28291277 | -1.26 | -1.77 | -0.75 | 0.42 | 0.31 | 0.56 |
| Thrombophlebitis | 40 | 171460 | 2913 | 28305550 | 1.15 | 0.62 | 1.68 | 2.23 | 1.63 | 3.05 |
| Venous thrombosis limb | 40 | 171460 | 2728 | 28305735 | 1.24 | 0.71 | 1.77 | 2.38 | 1.74 | 3.25 |
| Fluid overload | 39 | 171461 | 10296 | 28298167 | -0.67 | -1.20 | -0.13 | 0.63 | 0.46 | 0.86 |
| Injection site haemorrhage | 39 | 171461 | 24867 | 28283596 | -1.93 | -2.47 | -1.39 | 0.26 | 0.19 | 0.36 |
| Osteonecrosis | 39 | 171461 | 9605 | 28298858 | -0.57 | -1.11 | -0.03 | 0.67 | 0.49 | 0.92 |
| Pulmonary congestion | 39 | 171461 | 6208 | 28302255 | 0.05 | -0.49 | 0.59 | 1.04 | 0.76 | 1.42 |
| Venoocclusive liver disease | 39 | 171461 | 6250 | 28302213 | 0.04 | -0.49 | 0.58 | 1.03 | 0.75 | 1.41 |
| Cardiovascular disorder | 38 | 171462 | 6137 | 28302326 | 0.03 | -0.51 | 0.57 | 1.02 | 0.74 | 1.41 |
| Coronary artery occlusion | 38 | 171462 | 6003 | 28302460 | 0.06 | -0.48 | 0.61 | 1.04 | 0.76 | 1.44 |
| Intestinal haemorrhage | 38 | 171462 | 3003 | 28305460 | 1.03 | 0.49 | 1.58 | 2.06 | 1.50 | 2.84 |
| Venous thrombosis | 38 | 171462 | 3530 | 28304933 | 0.81 | 0.26 | 1.35 | 1.76 | 1.28 | 2.42 |
| Hypertensive nephropathy | 37 | 171463 | 373 | 28308090 | 3.66 | 3.11 | 4.21 | 13.59 | 9.69 | 19.05 |
| Pulseless electrical activity | 37 | 171463 | 2353 | 28306110 | 1.33 | 0.78 | 1.88 | 2.54 | 1.84 | 3.52 |
| Ascites | 36 | 171464 | 10731 | 28297732 | -0.84 | -1.40 | -0.28 | 0.56 | 0.40 | 0.77 |
| Gastric ulcer haemorrhage | 36 | 171464 | 4864 | 28303599 | 0.28 | -0.28 | 0.84 | 1.22 | 0.88 | 1.69 |
| Pericarditis | 36 | 171464 | 6142 | 28302321 | -0.05 | -0.61 | 0.51 | 0.97 | 0.70 | 1.34 |
| Myocarditis | 35 | 171465 | 5738 | 28302725 | 0.01 | -0.56 | 0.58 | 1.01 | 0.72 | 1.40 |
| Pulmonary haemorrhage | 35 | 171465 | 5641 | 28302822 | 0.03 | -0.53 | 0.60 | 1.02 | 0.73 | 1.43 |
| Small intestinal haemorrhage | 35 | 171465 | 2712 | 28305751 | 1.06 | 0.49 | 1.63 | 2.10 | 1.50 | 2.93 |
| Torsade de pointes | 35 | 171465 | 5429 | 28303034 | 0.09 | -0.48 | 0.66 | 1.06 | 0.76 | 1.48 |
| Haemorrhoidal haemorrhage | 34 | 171466 | 4558 | 28303905 | 0.29 | -0.28 | 0.87 | 1.23 | 0.88 | 1.72 |
| Vascular purpura | 34 | 171466 | 1563 | 28306900 | 1.77 | 1.19 | 2.35 | 3.46 | 2.46 | 4.86 |
| Oesophageal varices haemorrhage | 33 | 171467 | 2132 | 28306331 | 1.31 | 0.72 | 1.89 | 2.50 | 1.77 | 3.52 |
| Tachyarrhythmia | 33 | 171467 | 2329 | 28306134 | 1.19 | 0.60 | 1.77 | 2.29 | 1.63 | 3.23 |
| Tricuspid valve incompetence | 33 | 171467 | 3002 | 28305461 | 0.84 | 0.25 | 1.42 | 1.79 | 1.27 | 2.53 |
| Cerebral ischaemia | 31 | 171469 | 2655 | 28305808 | 0.92 | 0.31 | 1.52 | 1.90 | 1.33 | 2.71 |
| Subdural haemorrhage | 31 | 171469 | 3378 | 28305085 | 0.58 | -0.02 | 1.19 | 1.50 | 1.05 | 2.14 |
| Vascular stent thrombosis | 31 | 171469 | 3581 | 28304882 | 0.50 | -0.10 | 1.11 | 1.42 | 1.00 | 2.02 |
| Ventricular arrhythmia | 31 | 171469 | 2587 | 28305876 | 0.95 | 0.35 | 1.56 | 1.95 | 1.37 | 2.78 |
| Renal artery stenosis | 30 | 171470 | 658 | 28307805 | 2.72 | 2.10 | 3.33 | 6.80 | 4.72 | 9.80 |
| Skin haemorrhage | 30 | 171470 | 6321 | 28302142 | -0.35 | -0.96 | 0.27 | 0.79 | 0.55 | 1.13 |
| Mitral valve incompetence | 29 | 171471 | 3849 | 28304614 | 0.31 | -0.32 | 0.93 | 1.24 | 0.86 | 1.78 |
| Stress cardiomyopathy | 29 | 171471 | 4850 | 28303613 | -0.02 | -0.64 | 0.61 | 0.99 | 0.69 | 1.42 |
| Circulatory collapse | 28 | 171472 | 6432 | 28302031 | -0.47 | -1.10 | 0.17 | 0.72 | 0.50 | 1.05 |
| Embolism | 28 | 171472 | 5651 | 28302812 | -0.28 | -0.92 | 0.35 | 0.82 | 0.57 | 1.19 |
| Haemorrhagic arteriovenous malformation | 28 | 171472 | 2808 | 28305655 | 0.70 | 0.06 | 1.33 | 1.63 | 1.12 | 2.36 |
| Petechiae | 28 | 171472 | 4871 | 28303592 | -0.07 | -0.71 | 0.56 | 0.95 | 0.65 | 1.38 |
| Purpura | 28 | 171472 | 4354 | 28304109 | 0.08 | -0.55 | 0.72 | 1.06 | 0.73 | 1.54 |
| Thrombotic thrombocytopenic purpura | 28 | 171472 | 2916 | 28305547 | 0.64 | 0.01 | 1.28 | 1.57 | 1.08 | 2.28 |
| Blood pressure fluctuation | 27 | 171473 | 8350 | 28300113 | -0.89 | -1.54 | -0.24 | 0.54 | 0.37 | 0.79 |
| Peripheral arterial occlusive disease | 26 | 171474 | 3210 | 28305253 | 0.41 | -0.26 | 1.07 | 1.33 | 0.90 | 1.95 |
| Varices oesophageal | 26 | 171474 | 2018 | 28306445 | 1.05 | 0.39 | 1.71 | 2.08 | 1.41 | 3.07 |
| Hypovolaemic shock | 25 | 171475 | 2702 | 28305761 | 0.59 | -0.08 | 1.27 | 1.51 | 1.02 | 2.24 |
| Lymphoedema | 25 | 171475 | 3132 | 28305331 | 0.39 | -0.29 | 1.06 | 1.31 | 0.88 | 1.94 |
| Myocardial ischaemia | 25 | 171475 | 5814 | 28302649 | -0.48 | -1.16 | 0.19 | 0.71 | 0.48 | 1.06 |
| Peripheral coldness | 25 | 171475 | 6049 | 28302414 | -0.54 | -1.22 | 0.14 | 0.69 | 0.46 | 1.02 |
| Intraventricular haemorrhage | 24 | 171476 | 2170 | 28306293 | 0.84 | 0.15 | 1.53 | 1.80 | 1.20 | 2.68 |
| Left ventricular failure | 24 | 171476 | 2407 | 28306056 | 0.69 | 0.00 | 1.39 | 1.62 | 1.09 | 2.43 |
| Poor venous access | 24 | 171476 | 3181 | 28305282 | 0.31 | -0.38 | 1.00 | 1.24 | 0.83 | 1.85 |
| Varicose vein | 24 | 171476 | 3359 | 28305104 | 0.23 | -0.46 | 0.92 | 1.18 | 0.79 | 1.76 |
| Acute coronary syndrome | 23 | 171477 | 4861 | 28303602 | -0.35 | -1.05 | 0.36 | 0.78 | 0.52 | 1.18 |
| Congestive cardiomyopathy | 23 | 171477 | 2609 | 28305854 | 0.52 | -0.18 | 1.23 | 1.44 | 0.96 | 2.17 |
| Renal hypertension | 23 | 171477 | 352 | 28308111 | 3.09 | 2.38 | 3.80 | 8.93 | 5.86 | 13.61 |
| Cardiomyopathy | 22 | 171478 | 6233 | 28302230 | -0.76 | -1.49 | -0.04 | 0.59 | 0.39 | 0.89 |
| Gingival bleeding | 22 | 171478 | 5360 | 28303103 | -0.55 | -1.27 | 0.17 | 0.68 | 0.45 | 1.04 |
| Prerenal failure | 22 | 171478 | 1430 | 28307033 | 1.28 | 0.56 | 2.01 | 2.46 | 1.61 | 3.74 |
| Vascular occlusion | 22 | 171478 | 1745 | 28306718 | 1.01 | 0.29 | 1.74 | 2.03 | 1.33 | 3.09 |
| Left ventricular dysfunction | 21 | 171479 | 4482 | 28303981 | -0.36 | -1.10 | 0.38 | 0.78 | 0.51 | 1.19 |
| Mouth haemorrhage | 21 | 171479 | 3857 | 28304606 | -0.15 | -0.89 | 0.59 | 0.90 | 0.59 | 1.38 |
| Orthostatic intolerance | 21 | 171479 | 695 | 28307768 | 2.16 | 1.42 | 2.90 | 4.56 | 2.96 | 7.05 |
| Peripheral ischaemia | 21 | 171479 | 3039 | 28305424 | 0.18 | -0.56 | 0.92 | 1.14 | 0.74 | 1.75 |
| Portal vein thrombosis | 21 | 171479 | 1980 | 28306483 | 0.78 | 0.04 | 1.52 | 1.72 | 1.12 | 2.65 |
| Renal haemorrhage | 21 | 171479 | 2541 | 28305922 | 0.43 | -0.31 | 1.17 | 1.35 | 0.88 | 2.08 |
| Sudden cardiac death | 21 | 171479 | 2800 | 28305663 | 0.30 | -0.44 | 1.04 | 1.23 | 0.80 | 1.89 |
| Thrombophlebitis superficial | 21 | 171479 | 3325 | 28305138 | 0.06 | -0.68 | 0.80 | 1.04 | 0.68 | 1.60 |
| Diverticulum intestinal haemorrhagic | 20 | 171480 | 3232 | 28305231 | 0.03 | -0.73 | 0.79 | 1.02 | 0.66 | 1.58 |
| Eye haemorrhage | 20 | 171480 | 6040 | 28302423 | -0.85 | -1.61 | -0.09 | 0.55 | 0.36 | 0.86 |
| Large intestinal haemorrhage | 20 | 171480 | 1964 | 28306499 | 0.72 | -0.04 | 1.48 | 1.65 | 1.06 | 2.57 |
| Vascular device infection | 20 | 171480 | 1919 | 28306544 | 0.75 | -0.01 | 1.51 | 1.69 | 1.09 | 2.63 |
| Vascular stent stenosis | 20 | 171480 | 692 | 28307771 | 2.10 | 1.34 | 2.86 | 4.37 | 2.80 | 6.82 |
| Atrial flutter | 19 | 171481 | 4728 | 28303735 | -0.58 | -1.36 | 0.20 | 0.67 | 0.43 | 1.05 |
| Endocarditis | 19 | 171481 | 2236 | 28306227 | 0.47 | -0.31 | 1.25 | 1.39 | 0.88 | 2.18 |
| Phlebitis | 19 | 171481 | 2818 | 28305645 | 0.15 | -0.63 | 0.93 | 1.11 | 0.71 | 1.74 |
| Thrombotic microangiopathy | 19 | 171481 | 7788 | 28300675 | -1.28 | -2.07 | -0.50 | 0.41 | 0.26 | 0.64 |
| Vein disorder | 19 | 171481 | 3347 | 28305116 | -0.09 | -0.87 | 0.69 | 0.94 | 0.60 | 1.47 |
| Atrioventricular block complete | 18 | 171482 | 3176 | 28305287 | -0.09 | -0.90 | 0.71 | 0.94 | 0.59 | 1.49 |
| Cardiomegaly | 18 | 171482 | 3286 | 28305177 | -0.14 | -0.94 | 0.66 | 0.91 | 0.57 | 1.44 |
| Increased tendency to bruise | 18 | 171482 | 4632 | 28303831 | -0.62 | -1.43 | 0.18 | 0.65 | 0.41 | 1.03 |
| Retinal vein occlusion | 18 | 171482 | 1888 | 28306575 | 0.63 | -0.18 | 1.43 | 1.55 | 0.97 | 2.47 |
| Stoma site haemorrhage | 18 | 171482 | 1380 | 28307083 | 1.05 | 0.25 | 1.86 | 2.09 | 1.31 | 3.32 |
| Cardiogenic shock | 17 | 171483 | 4828 | 28303635 | -0.76 | -1.59 | 0.07 | 0.59 | 0.37 | 0.95 |
| Colitis ischaemic | 17 | 171483 | 3825 | 28304638 | -0.43 | -1.26 | 0.40 | 0.74 | 0.46 | 1.19 |
| Coronary artery stenosis | 17 | 171483 | 2037 | 28306426 | 0.44 | -0.39 | 1.27 | 1.36 | 0.85 | 2.20 |
| Retroperitoneal haematoma | 17 | 171483 | 1883 | 28306580 | 0.55 | -0.28 | 1.38 | 1.47 | 0.91 | 2.37 |
| Vascular pseudoaneurysm | 17 | 171483 | 2016 | 28306447 | 0.46 | -0.37 | 1.29 | 1.38 | 0.85 | 2.22 |
| Vein rupture | 17 | 171483 | 1346 | 28307117 | 1.01 | 0.18 | 1.84 | 2.02 | 1.25 | 3.26 |
| Venous occlusion | 17 | 171483 | 1380 | 28307083 | 0.97 | 0.14 | 1.80 | 1.98 | 1.22 | 3.19 |
| Disseminated intravascular coagulation | 16 | 171484 | 5062 | 28303401 | -0.91 | -1.77 | -0.06 | 0.53 | 0.32 | 0.86 |
| Dizziness postural | 16 | 171484 | 3463 | 28305000 | -0.38 | -1.23 | 0.48 | 0.77 | 0.47 | 1.26 |
| Gastritis haemorrhagic | 16 | 171484 | 1461 | 28307002 | 0.81 | -0.04 | 1.67 | 1.76 | 1.08 | 2.89 |
| Haemorrhage subcutaneous | 16 | 171484 | 1889 | 28306574 | 0.46 | -0.39 | 1.32 | 1.38 | 0.84 | 2.26 |
| Intestinal ischaemia | 16 | 171484 | 2878 | 28305585 | -0.12 | -0.98 | 0.74 | 0.92 | 0.56 | 1.50 |
| Subcutaneous haematoma | 16 | 171484 | 1577 | 28306886 | 0.71 | -0.15 | 1.57 | 1.64 | 1.00 | 2.69 |
| Urinary bladder haemorrhage | 16 | 171484 | 2359 | 28306104 | 0.16 | -0.70 | 1.01 | 1.12 | 0.68 | 1.82 |
| Vascular graft complication | 16 | 171484 | 151 | 28308312 | 3.45 | 2.60 | 4.31 | 11.66 | 6.97 | 19.53 |
| Duodenal ulcer haemorrhage | 15 | 171485 | 2409 | 28306054 | 0.04 | -0.85 | 0.92 | 1.03 | 0.62 | 1.71 |
| Raynaud’s phenomenon | 15 | 171485 | 2951 | 28305512 | -0.24 | -1.13 | 0.64 | 0.84 | 0.51 | 1.40 |
| Haemarthrosis | 14 | 171486 | 3812 | 28304651 | -0.70 | -1.62 | 0.22 | 0.61 | 0.36 | 1.04 |
| Heparin-induced thrombocytopenia | 14 | 171486 | 1473 | 28306990 | 0.62 | -0.30 | 1.54 | 1.54 | 0.91 | 2.60 |
| Infarction | 14 | 171486 | 3365 | 28305098 | -0.52 | -1.44 | 0.40 | 0.69 | 0.41 | 1.17 |
| Muscle haemorrhage | 14 | 171486 | 3084 | 28305379 | -0.40 | -1.32 | 0.52 | 0.76 | 0.45 | 1.28 |
| Peripheral artery occlusion | 14 | 171486 | 1323 | 28307140 | 0.76 | -0.16 | 1.68 | 1.70 | 1.01 | 2.88 |
| Peripheral vascular disorder | 14 | 171486 | 2790 | 28305673 | -0.26 | -1.18 | 0.66 | 0.83 | 0.49 | 1.41 |
| Embolic stroke | 13 | 171487 | 3367 | 28305096 | -0.63 | -1.59 | 0.33 | 0.65 | 0.37 | 1.11 |
| Haemorrhage urinary tract | 13 | 171487 | 1680 | 28306783 | 0.34 | -0.62 | 1.29 | 1.26 | 0.73 | 2.18 |
| Haemothorax | 13 | 171487 | 2344 | 28306119 | -0.12 | -1.08 | 0.84 | 0.92 | 0.53 | 1.58 |
| Mallory-weiss syndrome | 13 | 171487 | 929 | 28307534 | 1.13 | 0.17 | 2.09 | 2.20 | 1.27 | 3.81 |
| Renal artery arteriosclerosis | 13 | 171487 | 116 | 28308347 | 3.40 | 2.44 | 4.36 | 11.22 | 6.33 | 19.91 |
| Sinus node dysfunction | 13 | 171487 | 2848 | 28305615 | -0.39 | -1.35 | 0.56 | 0.76 | 0.44 | 1.31 |
| Ventricular hypokinesia | 13 | 171487 | 1925 | 28306538 | 0.15 | -0.81 | 1.11 | 1.11 | 0.64 | 1.92 |
| Bundle branch block left | 12 | 171488 | 1306 | 28307157 | 0.57 | -0.43 | 1.57 | 1.49 | 0.84 | 2.62 |
| Cardiac failure acute | 12 | 171488 | 4227 | 28304236 | -1.06 | -2.06 | -0.06 | 0.48 | 0.27 | 0.84 |
| Cyanosis | 12 | 171488 | 4126 | 28304337 | -1.02 | -2.02 | -0.02 | 0.49 | 0.28 | 0.86 |
| Ecchymosis | 12 | 171488 | 2433 | 28306030 | -0.28 | -1.29 | 0.72 | 0.82 | 0.47 | 1.45 |
| Gastrointestinal ulcer haemorrhage | 12 | 171488 | 901 | 28307562 | 1.06 | 0.06 | 2.06 | 2.10 | 1.19 | 3.71 |
| Haemorrhagic transformation stroke | 12 | 171488 | 1716 | 28306747 | 0.20 | -0.80 | 1.20 | 1.15 | 0.65 | 2.02 |
| Peripheral venous disease | 12 | 171488 | 1328 | 28307135 | 0.54 | -0.46 | 1.55 | 1.46 | 0.83 | 2.58 |
| Renal infarct | 12 | 171488 | 728 | 28307735 | 1.33 | 0.33 | 2.34 | 2.55 | 1.44 | 4.50 |
| Retinal haemorrhage | 12 | 171488 | 4018 | 28304445 | -0.99 | -1.99 | 0.01 | 0.50 | 0.29 | 0.89 |
| Trisomy 18 | 12 | 171488 | 476 | 28307987 | 1.86 | 0.86 | 2.86 | 3.69 | 2.08 | 6.55 |
| Tumour haemorrhage | 12 | 171488 | 2583 | 28305880 | -0.37 | -1.37 | 0.63 | 0.77 | 0.44 | 1.36 |
| Ischaemic cerebral infarction | 11 | 171489 | 1045 | 28307418 | 0.75 | -0.30 | 1.80 | 1.68 | 0.93 | 3.05 |
| Optic ischaemic neuropathy | 11 | 171489 | 1211 | 28307252 | 0.55 | -0.50 | 1.60 | 1.47 | 0.81 | 2.66 |
| Postmenopausal haemorrhage | 11 | 171489 | 1393 | 28307070 | 0.36 | -0.69 | 1.41 | 1.29 | 0.71 | 2.33 |
| Retroperitoneal haemorrhage | 11 | 171489 | 1946 | 28306517 | -0.10 | -1.15 | 0.95 | 0.94 | 0.52 | 1.69 |
| Thrombosis in device | 11 | 171489 | 2175 | 28306288 | -0.25 | -1.30 | 0.80 | 0.84 | 0.46 | 1.52 |
| Traumatic haematoma | 11 | 171489 | 1165 | 28307298 | 0.60 | -0.45 | 1.65 | 1.52 | 0.84 | 2.76 |
| Acute pulmonary oedema | 10 | 171490 | 2313 | 28306150 | -0.46 | -1.57 | 0.64 | 0.72 | 0.39 | 1.35 |
| Angina unstable | 10 | 171490 | 4060 | 28304403 | -1.25 | -2.36 | -0.15 | 0.42 | 0.22 | 0.78 |
| Anti-neutrophil cytoplasmic antibody positive vasculitis | 10 | 171490 | 774 | 28307689 | 1.01 | -0.10 | 2.11 | 2.02 | 1.08 | 3.78 |
| Aortic aneurysm | 10 | 171490 | 3163 | 28305300 | -0.90 | -2.01 | 0.21 | 0.53 | 0.29 | 0.99 |
| Atrial thrombosis | 10 | 171490 | 1386 | 28307077 | 0.24 | -0.87 | 1.34 | 1.18 | 0.63 | 2.20 |
| Atrioventricular block | 10 | 171490 | 2960 | 28305503 | -0.81 | -1.91 | 0.30 | 0.57 | 0.31 | 1.06 |
| Atrioventricular block first degree | 10 | 171490 | 1606 | 28306857 | 0.04 | -1.07 | 1.14 | 1.03 | 0.55 | 1.91 |
| Heat stroke | 10 | 171490 | 777 | 28307686 | 1.00 | -0.10 | 2.11 | 2.02 | 1.08 | 3.76 |
| Hypertensive heart disease | 10 | 171490 | 900 | 28307563 | 0.81 | -0.29 | 1.92 | 1.76 | 0.95 | 3.29 |
| Iliac artery occlusion | 10 | 171490 | 260 | 28308203 | 2.30 | 1.20 | 3.41 | 5.06 | 2.69 | 9.52 |
| Oesophageal haemorrhage | 10 | 171490 | 893 | 28307570 | 0.82 | -0.28 | 1.93 | 1.78 | 0.95 | 3.31 |
| Pulmonary alveolar haemorrhage | 10 | 171490 | 3972 | 28304491 | -1.22 | -2.33 | -0.11 | 0.43 | 0.23 | 0.80 |
| Rectal ulcer haemorrhage | 10 | 171490 | 314 | 28308149 | 2.10 | 0.99 | 3.21 | 4.37 | 2.33 | 8.20 |
| Thalamus haemorrhage | 10 | 171490 | 1706 | 28306757 | -0.05 | -1.15 | 1.06 | 0.97 | 0.52 | 1.80 |
| Uterine haemorrhage | 10 | 171490 | 3019 | 28305444 | -0.84 | -1.94 | 0.27 | 0.56 | 0.30 | 1.04 |
| Arterial occlusive disease | 9 | 171491 | 3115 | 28305348 | -1.02 | -2.20 | 0.15 | 0.49 | 0.25 | 0.94 |
| Cardiac septal defect | 9 | 171491 | 327 | 28308136 | 1.91 | 0.74 | 3.09 | 3.83 | 1.97 | 7.43 |
| Cardiac valve disease | 9 | 171491 | 2145 | 28306318 | -0.50 | -1.68 | 0.67 | 0.70 | 0.37 | 1.35 |
| Coronary artery thrombosis | 9 | 171491 | 693 | 28307770 | 1.01 | -0.17 | 2.18 | 2.02 | 1.05 | 3.90 |
| Diastolic dysfunction | 9 | 171491 | 846 | 28307617 | 0.75 | -0.42 | 1.92 | 1.69 | 0.88 | 3.26 |
| Extrasystoles | 9 | 171491 | 2608 | 28305855 | -0.78 | -1.95 | 0.40 | 0.58 | 0.30 | 1.12 |
| Hepatic fibrosis | 9 | 171491 | 2584 | 28305879 | -0.76 | -1.94 | 0.41 | 0.59 | 0.31 | 1.13 |
| Ischaemia | 9 | 171491 | 1904 | 28306559 | -0.34 | -1.51 | 0.83 | 0.79 | 0.41 | 1.52 |
| Mucosal haemorrhage | 9 | 171491 | 519 | 28307944 | 1.37 | 0.19 | 2.54 | 2.61 | 1.35 | 5.04 |
| Portal hypertension | 9 | 171491 | 1386 | 28307077 | 0.09 | -1.08 | 1.27 | 1.07 | 0.55 | 2.06 |
| Respiratory tract haemorrhage | 9 | 171491 | 755 | 28307708 | 0.90 | -0.28 | 2.07 | 1.87 | 0.97 | 3.61 |
| Sinus arrest | 9 | 171491 | 1073 | 28307390 | 0.44 | -0.74 | 1.61 | 1.36 | 0.70 | 2.62 |
| Splenic haemorrhage | 9 | 171491 | 544 | 28307919 | 1.31 | 0.14 | 2.48 | 2.50 | 1.30 | 4.84 |
| Thalamic infarction | 9 | 171491 | 892 | 28307571 | 0.68 | -0.49 | 1.86 | 1.61 | 0.83 | 3.10 |
| Urticarial vasculitis | 9 | 171491 | 572 | 28307891 | 1.25 | 0.07 | 2.42 | 2.40 | 1.24 | 4.63 |
| White matter lesion | 9 | 171491 | 896 | 28307567 | 0.68 | -0.50 | 1.85 | 1.60 | 0.83 | 3.09 |
| Anal haemorrhage | 8 | 171492 | 1044 | 28307419 | 0.31 | -0.94 | 1.57 | 1.25 | 0.62 | 2.50 |
| Arteriosclerosis | 8 | 171492 | 2667 | 28305796 | -0.97 | -2.22 | 0.29 | 0.51 | 0.25 | 1.02 |
| Atrioventricular block second degree | 8 | 171492 | 1445 | 28307018 | -0.12 | -1.38 | 1.13 | 0.92 | 0.46 | 1.84 |
| Cardiac failure chronic | 8 | 171492 | 2081 | 28306382 | -0.62 | -1.88 | 0.63 | 0.65 | 0.32 | 1.30 |
| Cardiac tamponade | 8 | 171492 | 2728 | 28305735 | -1.00 | -2.25 | 0.26 | 0.50 | 0.25 | 1.00 |
| Dyspnoea at rest | 8 | 171492 | 1229 | 28307234 | 0.10 | -1.16 | 1.35 | 1.07 | 0.53 | 2.14 |
| Essential hypertension | 8 | 171492 | 882 | 28307581 | 0.54 | -0.72 | 1.79 | 1.45 | 0.73 | 2.92 |
| Hypertensive emergency | 8 | 171492 | 781 | 28307682 | 0.69 | -0.56 | 1.95 | 1.62 | 0.81 | 3.26 |
| Injection site haematoma | 8 | 171492 | 1425 | 28307038 | -0.10 | -1.36 | 1.15 | 0.93 | 0.46 | 1.86 |
| Intra-abdominal haematoma | 8 | 171492 | 968 | 28307495 | 0.41 | -0.84 | 1.67 | 1.34 | 0.67 | 2.68 |
| Intracardiac thrombus | 8 | 171492 | 1693 | 28306770 | -0.34 | -1.59 | 0.92 | 0.79 | 0.39 | 1.58 |
| Intracranial aneurysm | 8 | 171492 | 2076 | 28306387 | -0.62 | -1.87 | 0.64 | 0.65 | 0.32 | 1.30 |
| Ischaemic cardiomyopathy | 8 | 171492 | 1493 | 28306970 | -0.17 | -1.42 | 1.09 | 0.89 | 0.44 | 1.78 |
| Left ventricular hypertrophy | 8 | 171492 | 1340 | 28307123 | -0.02 | -1.27 | 1.24 | 0.99 | 0.49 | 1.98 |
| Localised oedema | 8 | 171492 | 1826 | 28306637 | -0.44 | -1.70 | 0.81 | 0.74 | 0.37 | 1.47 |
| Long qt syndrome | 8 | 171492 | 1982 | 28306481 | -0.55 | -1.81 | 0.70 | 0.68 | 0.34 | 1.36 |
| Migraine with aura | 8 | 171492 | 1322 | 28307141 | 0.00 | -1.26 | 1.25 | 1.00 | 0.50 | 2.00 |
| Orthopnoea | 8 | 171492 | 1448 | 28307015 | -0.12 | -1.38 | 1.13 | 0.92 | 0.46 | 1.84 |
| Peritoneal haemorrhage | 8 | 171492 | 1350 | 28307113 | -0.03 | -1.28 | 1.23 | 0.98 | 0.49 | 1.96 |
| Pharyngeal haemorrhage | 8 | 171492 | 811 | 28307652 | 0.65 | -0.61 | 1.90 | 1.57 | 0.78 | 3.15 |
| Poor peripheral circulation | 8 | 171492 | 2267 | 28306196 | -0.74 | -2.00 | 0.51 | 0.60 | 0.30 | 1.20 |
| Post procedural haematoma | 8 | 171492 | 1605 | 28306858 | -0.26 | -1.52 | 0.99 | 0.83 | 0.42 | 1.67 |
| Pulmonary artery stenosis | 8 | 171492 | 308 | 28308155 | 1.82 | 0.57 | 3.08 | 3.59 | 1.78 | 7.25 |
| Stasis dermatitis | 8 | 171492 | 626 | 28307837 | 0.98 | -0.28 | 2.23 | 1.98 | 0.99 | 3.98 |
| Vertebral artery stenosis | 8 | 171492 | 82 | 28308381 | 3.03 | 1.77 | 4.28 | 8.53 | 4.13 | 17.62 |
| Cardiac flutter | 7 | 171493 | 2557 | 28305906 | -1.09 | -2.44 | 0.27 | 0.47 | 0.22 | 0.98 |
| Cardiopulmonary failure | 7 | 171493 | 1651 | 28306812 | -0.48 | -1.84 | 0.87 | 0.71 | 0.34 | 1.50 |
| Cerebellar ischaemia | 7 | 171493 | 70 | 28308393 | 2.96 | 1.60 | 4.32 | 8.12 | 3.73 | 17.65 |
| Cerebral haematoma | 7 | 171493 | 2100 | 28306363 | -0.81 | -2.17 | 0.54 | 0.57 | 0.27 | 1.19 |
| Coronary artery dissection | 7 | 171493 | 246 | 28308217 | 1.89 | 0.53 | 3.25 | 3.77 | 1.78 | 7.99 |
| Gastrointestinal necrosis | 7 | 171493 | 1323 | 28307140 | -0.18 | -1.54 | 1.17 | 0.88 | 0.42 | 1.85 |
| Henoch-schonlein purpura | 7 | 171493 | 903 | 28307560 | 0.33 | -1.03 | 1.68 | 1.26 | 0.60 | 2.64 |
| Intra-abdominal haemorrhage | 7 | 171493 | 1465 | 28306998 | -0.32 | -1.68 | 1.04 | 0.80 | 0.38 | 1.68 |
| Microangiopathic haemolytic anaemia | 7 | 171493 | 354 | 28308109 | 1.49 | 0.13 | 2.84 | 2.84 | 1.34 | 5.99 |
| Oesophagitis haemorrhagic | 7 | 171493 | 561 | 28307902 | 0.94 | -0.42 | 2.29 | 1.92 | 0.91 | 4.05 |
| Supraventricular extrasystoles | 7 | 171493 | 1655 | 28306808 | -0.49 | -1.84 | 0.87 | 0.71 | 0.34 | 1.50 |
| Traumatic intracranial haemorrhage | 7 | 171493 | 987 | 28307476 | 0.21 | -1.15 | 1.56 | 1.16 | 0.55 | 2.43 |
| Vena cava thrombosis | 7 | 171493 | 1502 | 28306961 | -0.35 | -1.71 | 1.00 | 0.78 | 0.37 | 1.64 |
| Viral myocarditis | 7 | 171493 | 289 | 28308174 | 1.72 | 0.36 | 3.07 | 3.33 | 1.57 | 7.05 |
| Abdominal wall haematoma | 6 | 171494 | 1546 | 28306917 | -0.60 | -2.08 | 0.88 | 0.66 | 0.30 | 1.47 |
| Atrial septal defect | 6 | 171494 | 2480 | 28305983 | -1.25 | -2.73 | 0.23 | 0.42 | 0.19 | 0.93 |
| Bundle branch block right | 6 | 171494 | 1225 | 28307238 | -0.28 | -1.77 | 1.20 | 0.82 | 0.37 | 1.83 |
| Cerebellar haemorrhage | 6 | 171494 | 1402 | 28307061 | -0.47 | -1.95 | 1.02 | 0.72 | 0.32 | 1.61 |
| Congenital cardiovascular anomaly | 6 | 171494 | 274 | 28308189 | 1.57 | 0.09 | 3.06 | 3.01 | 1.34 | 6.76 |
| Embolic cerebral infarction | 6 | 171494 | 677 | 28307786 | 0.49 | -0.99 | 1.98 | 1.41 | 0.63 | 3.16 |
| Haemorrhagic disorder | 6 | 171494 | 566 | 28307897 | 0.72 | -0.76 | 2.20 | 1.65 | 0.74 | 3.70 |
| Haemorrhagic erosive gastritis | 6 | 171494 | 580 | 28307883 | 0.69 | -0.79 | 2.17 | 1.62 | 0.72 | 3.62 |
| Heart valve incompetence | 6 | 171494 | 1305 | 28307158 | -0.37 | -1.85 | 1.11 | 0.77 | 0.35 | 1.72 |
| Livedo reticularis | 6 | 171494 | 706 | 28307757 | 0.44 | -1.04 | 1.92 | 1.36 | 0.61 | 3.04 |
| Necrosis | 6 | 171494 | 2069 | 28306394 | -1.00 | -2.48 | 0.48 | 0.50 | 0.22 | 1.11 |
| Nocturnal dyspnoea | 6 | 171494 | 727 | 28307736 | 0.40 | -1.08 | 1.89 | 1.33 | 0.59 | 2.96 |
| Nodal arrhythmia | 6 | 171494 | 381 | 28308082 | 1.20 | -0.28 | 2.68 | 2.31 | 1.03 | 5.18 |
| Nodal rhythm | 6 | 171494 | 577 | 28307886 | 0.70 | -0.79 | 2.18 | 1.63 | 0.73 | 3.64 |
| Papilloedema | 6 | 171494 | 2145 | 28306318 | -1.05 | -2.53 | 0.43 | 0.48 | 0.22 | 1.07 |
| Pulmonary artery stenosis congenital | 6 | 171494 | 341 | 28308122 | 1.33 | -0.16 | 2.81 | 2.53 | 1.13 | 5.68 |
| Spinal cord haemorrhage | 6 | 171494 | 410 | 28308053 | 1.11 | -0.37 | 2.60 | 2.18 | 0.97 | 4.88 |
| Subclavian vein thrombosis | 6 | 171494 | 900 | 28307563 | 0.13 | -1.36 | 1.61 | 1.09 | 0.49 | 2.44 |
| Temporal arteritis | 6 | 171494 | 1273 | 28307190 | -0.34 | -1.82 | 1.15 | 0.79 | 0.35 | 1.77 |
| Toxic shock syndrome | 6 | 171494 | 360 | 28308103 | 1.27 | -0.22 | 2.75 | 2.42 | 1.08 | 5.43 |
| Traumatic haemothorax | 6 | 171494 | 455 | 28308008 | 0.99 | -0.49 | 2.47 | 2.00 | 0.89 | 4.47 |
| Vascular compression | 6 | 171494 | 240 | 28308223 | 1.71 | 0.23 | 3.20 | 3.33 | 1.48 | 7.48 |
| Vascular injury | 6 | 171494 | 974 | 28307489 | 0.02 | -1.46 | 1.51 | 1.02 | 0.46 | 2.27 |
| Vascular pain | 6 | 171494 | 732 | 28307731 | 0.39 | -1.09 | 1.88 | 1.32 | 0.59 | 2.94 |
| Vascular rupture | 6 | 171494 | 1049 | 28307414 | -0.08 | -1.56 | 1.41 | 0.95 | 0.43 | 2.12 |
| Vascular stent occlusion | 6 | 171494 | 764 | 28307699 | 0.34 | -1.14 | 1.82 | 1.27 | 0.57 | 2.83 |
| Vertebral artery occlusion | 6 | 171494 | 305 | 28308158 | 1.45 | -0.03 | 2.94 | 2.77 | 1.23 | 6.21 |
| Aortic valve stenosis | 5 | 171495 | 699 | 28307764 | 0.21 | -1.44 | 1.87 | 1.16 | 0.48 | 2.80 |
| Arterial haemorrhage | 5 | 171495 | 579 | 28307884 | 0.45 | -1.20 | 2.11 | 1.37 | 0.57 | 3.31 |
| Arteriospasm coronary | 5 | 171495 | 1640 | 28306823 | -0.92 | -2.57 | 0.73 | 0.53 | 0.22 | 1.27 |
| Brain stem infarction | 5 | 171495 | 698 | 28307765 | 0.22 | -1.44 | 1.87 | 1.16 | 0.48 | 2.80 |
| Cardiac dysfunction | 5 | 171495 | 1045 | 28307418 | -0.31 | -1.97 | 1.34 | 0.81 | 0.33 | 1.94 |
| Cutaneous vasculitis | 5 | 171495 | 709 | 28307754 | 0.20 | -1.46 | 1.85 | 1.15 | 0.48 | 2.76 |
| Gastrointestinal polyp haemorrhage | 5 | 171495 | 761 | 28307702 | 0.11 | -1.55 | 1.76 | 1.08 | 0.45 | 2.59 |
| Gravitational oedema | 5 | 171495 | 391 | 28308072 | 0.93 | -0.72 | 2.59 | 1.92 | 0.79 | 4.63 |
| Haemangioma | 5 | 171495 | 739 | 28307724 | 0.14 | -1.51 | 1.80 | 1.11 | 0.46 | 2.66 |
| Haemodynamic instability | 5 | 171495 | 2080 | 28306383 | -1.25 | -2.90 | 0.41 | 0.42 | 0.17 | 1.01 |
| Heart disease congenital | 5 | 171495 | 1500 | 28306963 | -0.80 | -2.45 | 0.86 | 0.57 | 0.24 | 1.38 |
| Hyperaemia | 5 | 171495 | 611 | 28307852 | 0.39 | -1.27 | 2.04 | 1.31 | 0.54 | 3.16 |
| Incision site haemorrhage | 5 | 171495 | 515 | 28307948 | 0.60 | -1.06 | 2.25 | 1.52 | 0.63 | 3.67 |
| Infusion site bruising | 5 | 171495 | 634 | 28307829 | 0.34 | -1.32 | 1.99 | 1.27 | 0.53 | 3.05 |
| Intestinal infarction | 5 | 171495 | 374 | 28308089 | 0.98 | -0.67 | 2.64 | 1.99 | 0.82 | 4.81 |
| Intracranial tumour haemorrhage | 5 | 171495 | 325 | 28308138 | 1.14 | -0.51 | 2.80 | 2.23 | 0.92 | 5.39 |
| Left atrial dilatation | 5 | 171495 | 331 | 28308132 | 1.12 | -0.53 | 2.78 | 2.20 | 0.91 | 5.31 |
| Lymphocele | 5 | 171495 | 419 | 28308044 | 0.85 | -0.81 | 2.50 | 1.81 | 0.75 | 4.37 |
| Mitral valve disease | 5 | 171495 | 933 | 28307530 | -0.16 | -1.82 | 1.49 | 0.89 | 0.37 | 2.15 |
| Peripheral artery thrombosis | 5 | 171495 | 1185 | 28307278 | -0.48 | -2.13 | 1.18 | 0.72 | 0.30 | 1.72 |
| Post thrombotic syndrome | 5 | 171495 | 296 | 28308167 | 1.25 | -0.40 | 2.90 | 2.40 | 0.99 | 5.80 |
| Prinzmetal angina | 5 | 171495 | 1079 | 28307384 | -0.35 | -2.01 | 1.30 | 0.78 | 0.32 | 1.88 |
| Procedural haemorrhage | 5 | 171495 | 2610 | 28305853 | -1.56 | -3.22 | 0.09 | 0.34 | 0.14 | 0.81 |
| Renal ischaemia | 5 | 171495 | 276 | 28308187 | 1.33 | -0.33 | 2.98 | 2.53 | 1.05 | 6.13 |
| Renal vessel disorder | 5 | 171495 | 128 | 28308335 | 2.08 | 0.43 | 3.73 | 4.31 | 1.76 | 10.54 |
| Stroke in evolution | 5 | 171495 | 474 | 28307989 | 0.70 | -0.95 | 2.36 | 1.63 | 0.68 | 3.94 |
| Superior vena cava syndrome | 5 | 171495 | 520 | 28307943 | 0.59 | -1.07 | 2.24 | 1.51 | 0.62 | 3.64 |
| Systemic scleroderma | 5 | 171495 | 430 | 28308033 | 0.82 | -0.84 | 2.47 | 1.77 | 0.73 | 4.28 |
| Traumatic haemorrhage | 5 | 171495 | 1203 | 28307260 | -0.50 | -2.15 | 1.16 | 0.71 | 0.29 | 1.70 |
| Urethral haemorrhage | 5 | 171495 | 401 | 28308062 | 0.90 | -0.75 | 2.56 | 1.88 | 0.78 | 4.54 |
| Vasculitis necrotising | 5 | 171495 | 440 | 28308023 | 0.79 | -0.86 | 2.45 | 1.74 | 0.72 | 4.20 |
| Vasodilatation | 5 | 171495 | 1982 | 28306481 | -1.18 | -2.84 | 0.47 | 0.44 | 0.18 | 1.06 |
| Vasoplegia syndrome | 5 | 171495 | 981 | 28307482 | -0.23 | -1.88 | 1.43 | 0.85 | 0.35 | 2.06 |
| Vasospasm | 5 | 171495 | 413 | 28308050 | 0.87 | -0.79 | 2.52 | 1.83 | 0.76 | 4.43 |
| Vessel puncture site haematoma | 5 | 171495 | 136 | 28308327 | 2.03 | 0.37 | 3.68 | 4.15 | 1.70 | 10.14 |
| Acute left ventricular failure | 4 | 171496 | 406 | 28308057 | 0.60 | -1.30 | 2.50 | 1.52 | 0.57 | 4.07 |
| Aortic arteriosclerosis | 4 | 171496 | 535 | 28307928 | 0.26 | -1.64 | 2.16 | 1.20 | 0.45 | 3.22 |
| Aortic stenosis | 4 | 171496 | 1400 | 28307063 | -0.99 | -2.89 | 0.91 | 0.50 | 0.19 | 1.34 |
| Cardiorenal syndrome | 4 | 171496 | 241 | 28308222 | 1.19 | -0.71 | 3.09 | 2.30 | 0.85 | 6.17 |
| Cerebral thrombosis | 4 | 171496 | 1367 | 28307096 | -0.96 | -2.86 | 0.94 | 0.51 | 0.19 | 1.37 |
| Cerebrovascular disorder | 4 | 171496 | 1056 | 28307407 | -0.61 | -2.51 | 1.29 | 0.65 | 0.24 | 1.74 |
| Conduction disorder | 4 | 171496 | 708 | 28307755 | -0.09 | -1.99 | 1.81 | 0.94 | 0.35 | 2.51 |
| Conjunctival haemorrhage | 4 | 171496 | 1775 | 28306688 | -1.32 | -3.22 | 0.58 | 0.40 | 0.15 | 1.07 |
| Cushingoid | 4 | 171496 | 1080 | 28307383 | -0.64 | -2.54 | 1.26 | 0.64 | 0.24 | 1.71 |
| Diverticulitis intestinal haemorrhagic | 4 | 171496 | 333 | 28308130 | 0.83 | -1.07 | 2.73 | 1.79 | 0.67 | 4.79 |
| Embolism arterial | 4 | 171496 | 807 | 28307656 | -0.26 | -2.16 | 1.64 | 0.84 | 0.31 | 2.23 |
| Enterocolitis haemorrhagic | 4 | 171496 | 971 | 28307492 | -0.50 | -2.40 | 1.40 | 0.71 | 0.26 | 1.88 |
| Gangrene | 4 | 171496 | 2767 | 28305696 | -1.93 | -3.83 | -0.03 | 0.26 | 0.10 | 0.69 |
| Granulomatosis with polyangiitis | 4 | 171496 | 506 | 28307957 | 0.33 | -1.57 | 2.23 | 1.26 | 0.47 | 3.38 |
| Haematoma infection | 4 | 171496 | 373 | 28308090 | 0.70 | -1.20 | 2.60 | 1.63 | 0.61 | 4.37 |
| Haematoma muscle | 4 | 171496 | 246 | 28308217 | 1.17 | -0.73 | 3.07 | 2.26 | 0.84 | 6.07 |
| Haemorrhagic diathesis | 4 | 171496 | 1803 | 28306660 | -1.34 | -3.24 | 0.56 | 0.39 | 0.15 | 1.05 |
| Ischaemic nephropathy | 4 | 171496 | 20 | 28308443 | 2.80 | 0.90 | 4.70 | 7.24 | 2.48 | 21.20 |
| Kidney congestion | 4 | 171496 | 111 | 28308352 | 1.92 | 0.02 | 3.82 | 3.84 | 1.42 | 10.41 |
| Labile blood pressure | 4 | 171496 | 510 | 28307953 | 0.32 | -1.58 | 2.22 | 1.25 | 0.47 | 3.35 |
| Mitral valve prolapse | 4 | 171496 | 770 | 28307693 | -0.20 | -2.10 | 1.70 | 0.87 | 0.33 | 2.33 |
| Oesophageal ulcer haemorrhage | 4 | 171496 | 399 | 28308064 | 0.62 | -1.28 | 2.52 | 1.54 | 0.58 | 4.13 |
| Oral contusion | 4 | 171496 | 114 | 28308349 | 1.89 | -0.01 | 3.79 | 3.78 | 1.39 | 10.24 |
| Pelvic haematoma | 4 | 171496 | 333 | 28308130 | 0.83 | -1.07 | 2.73 | 1.79 | 0.67 | 4.79 |
| Peripheral artery stenosis | 4 | 171496 | 633 | 28307830 | 0.05 | -1.85 | 1.95 | 1.04 | 0.39 | 2.77 |
| Peripheral embolism | 4 | 171496 | 724 | 28307739 | -0.12 | -2.02 | 1.78 | 0.92 | 0.34 | 2.46 |
| Renal cyst haemorrhage | 4 | 171496 | 269 | 28308194 | 1.07 | -0.83 | 2.97 | 2.11 | 0.79 | 5.67 |
| Retinal artery occlusion | 4 | 171496 | 1455 | 28307008 | -1.05 | -2.95 | 0.85 | 0.48 | 0.18 | 1.29 |
| Retinal vascular occlusion | 4 | 171496 | 420 | 28308043 | 0.56 | -1.34 | 2.46 | 1.48 | 0.55 | 3.96 |
| Retinopathy | 4 | 171496 | 1765 | 28306698 | -1.31 | -3.21 | 0.59 | 0.40 | 0.15 | 1.07 |
| Rheumatic fever | 4 | 171496 | 244 | 28308219 | 1.17 | -0.73 | 3.07 | 2.27 | 0.85 | 6.11 |
| Shock symptom | 4 | 171496 | 314 | 28308149 | 0.90 | -1.00 | 2.80 | 1.87 | 0.70 | 5.02 |
| Silent myocardial infarction | 4 | 171496 | 424 | 28308039 | 0.55 | -1.35 | 2.45 | 1.47 | 0.55 | 3.93 |
| Spinal subdural haematoma | 4 | 171496 | 157 | 28308306 | 1.61 | -0.29 | 3.51 | 3.10 | 1.15 | 8.37 |
| Splenic thrombosis | 4 | 171496 | 162 | 28308301 | 1.59 | -0.31 | 3.49 | 3.04 | 1.13 | 8.19 |
| Spontaneous haematoma | 4 | 171496 | 682 | 28307781 | -0.04 | -1.94 | 1.86 | 0.97 | 0.36 | 2.60 |
| Strangulated hernia | 4 | 171496 | 263 | 28308200 | 1.09 | -0.81 | 2.99 | 2.15 | 0.80 | 5.77 |
| Systolic dysfunction | 4 | 171496 | 654 | 28307809 | 0.01 | -1.89 | 1.91 | 1.01 | 0.38 | 2.70 |
| Tumour necrosis | 4 | 171496 | 789 | 28307674 | -0.23 | -2.13 | 1.67 | 0.85 | 0.32 | 2.28 |
| Vascular graft occlusion | 4 | 171496 | 214 | 28308249 | 1.31 | -0.59 | 3.21 | 2.50 | 0.93 | 6.74 |
| Vasculitic rash | 4 | 171496 | 867 | 28307596 | -0.35 | -2.25 | 1.55 | 0.78 | 0.29 | 2.09 |
| Venoocclusive disease | 4 | 171496 | 2777 | 28305686 | -1.94 | -3.84 | -0.04 | 0.26 | 0.10 | 0.69 |
| Ventricular dysfunction | 4 | 171496 | 934 | 28307529 | -0.45 | -2.35 | 1.45 | 0.73 | 0.27 | 1.95 |
| Ventricular failure | 4 | 171496 | 199 | 28308264 | 1.39 | -0.51 | 3.29 | 2.64 | 0.98 | 7.10 |
| Vitreous haemorrhage | 4 | 171496 | 2662 | 28305801 | -1.88 | -3.78 | 0.02 | 0.27 | 0.10 | 0.72 |
| Anaphylactoid reaction | 3 | 171497 | 1566 | 28306897 | -1.51 | -3.80 | 0.78 | 0.35 | 0.11 | 1.09 |
| Anaphylactoid shock | 3 | 171497 | 326 | 28308137 | 0.50 | -1.79 | 2.79 | 1.41 | 0.45 | 4.41 |
| Application site haemorrhage | 3 | 171497 | 1737 | 28306726 | -1.65 | -3.94 | 0.64 | 0.32 | 0.10 | 0.99 |
| Arteriosclerosis coronary artery | 3 | 171497 | 1766 | 28306697 | -1.67 | -3.96 | 0.62 | 0.31 | 0.10 | 0.97 |
| Atheroembolism | 3 | 171497 | 89 | 28308374 | 1.73 | -0.56 | 4.02 | 3.37 | 1.07 | 10.64 |
| Basal ganglia haemorrhage | 3 | 171497 | 836 | 28307627 | -0.67 | -2.96 | 1.62 | 0.63 | 0.20 | 1.95 |
| Blood pressure inadequately controlled | 3 | 171497 | 1861 | 28306602 | -1.74 | -4.03 | 0.55 | 0.30 | 0.10 | 0.92 |
| Bloody peritoneal effluent | 3 | 171497 | 339 | 28308124 | 0.45 | -1.84 | 2.74 | 1.37 | 0.44 | 4.27 |
| Bundle branch block | 3 | 171497 | 291 | 28308172 | 0.62 | -1.67 | 2.91 | 1.55 | 0.50 | 4.82 |
| Carotid artery occlusion | 3 | 171497 | 1144 | 28307319 | -1.08 | -3.37 | 1.21 | 0.47 | 0.15 | 1.46 |
| Carotid artery stenosis | 3 | 171497 | 1134 | 28307329 | -1.07 | -3.36 | 1.22 | 0.47 | 0.15 | 1.47 |
| Catheter site haemorrhage | 3 | 171497 | 1113 | 28307350 | -1.04 | -3.33 | 1.25 | 0.48 | 0.16 | 1.50 |
| Cerebral artery occlusion | 3 | 171497 | 455 | 28308008 | 0.10 | -2.19 | 2.39 | 1.07 | 0.35 | 3.34 |
| Device related thrombosis | 3 | 171497 | 572 | 28307891 | -0.18 | -2.47 | 2.11 | 0.88 | 0.28 | 2.74 |
| Diabetic foot | 3 | 171497 | 1852 | 28306611 | -1.74 | -4.03 | 0.55 | 0.30 | 0.10 | 0.93 |
| Ear haemorrhage | 3 | 171497 | 903 | 28307560 | -0.77 | -3.06 | 1.52 | 0.59 | 0.19 | 1.82 |
| Extremity necrosis | 3 | 171497 | 844 | 28307619 | -0.68 | -2.97 | 1.61 | 0.62 | 0.20 | 1.94 |
| Eye contusion | 3 | 171497 | 965 | 28307498 | -0.85 | -3.14 | 1.44 | 0.55 | 0.18 | 1.71 |
| Gastrointestinal ischaemia | 3 | 171497 | 112 | 28308351 | 1.55 | -0.74 | 3.84 | 2.97 | 0.94 | 9.35 |
| Haemorrhagic cyst | 3 | 171497 | 203 | 28308260 | 1.01 | -1.28 | 3.30 | 2.02 | 0.65 | 6.33 |
| Haemorrhoids thrombosed | 3 | 171497 | 261 | 28308202 | 0.74 | -1.55 | 3.03 | 1.68 | 0.54 | 5.25 |
| Hepatic haemorrhage | 3 | 171497 | 386 | 28308077 | 0.30 | -1.99 | 2.59 | 1.23 | 0.40 | 3.84 |
| Hypertensive cardiomyopathy | 3 | 171497 | 144 | 28308319 | 1.34 | -0.95 | 3.63 | 2.55 | 0.81 | 8.00 |
| Hypoxic-ischaemic encephalopathy | 3 | 171497 | 1227 | 28307236 | -1.18 | -3.47 | 1.11 | 0.44 | 0.14 | 1.37 |
| Injection site phlebitis | 3 | 171497 | 108 | 28308355 | 1.58 | -0.71 | 3.87 | 3.03 | 0.96 | 9.55 |
| Lymphangiosis carcinomatosa | 3 | 171497 | 554 | 28307909 | -0.14 | -2.43 | 2.15 | 0.91 | 0.29 | 2.82 |
| Medical device site haemorrhage | 3 | 171497 | 157 | 28308306 | 1.26 | -1.03 | 3.55 | 2.41 | 0.77 | 7.56 |
| Mesenteric artery thrombosis | 3 | 171497 | 305 | 28308158 | 0.57 | -1.72 | 2.86 | 1.49 | 0.48 | 4.65 |
| Microangiopathy | 3 | 171497 | 493 | 28307970 | 0.01 | -2.28 | 2.30 | 1.00 | 0.32 | 3.12 |
| Non-cirrhotic portal hypertension | 3 | 171497 | 187 | 28308276 | 1.09 | -1.20 | 3.38 | 2.14 | 0.69 | 6.71 |
| Penile haemorrhage | 3 | 171497 | 585 | 28307878 | -0.21 | -2.50 | 2.08 | 0.87 | 0.28 | 2.69 |
| Periorbital haematoma | 3 | 171497 | 341 | 28308122 | 0.44 | -1.85 | 2.73 | 1.36 | 0.44 | 4.25 |
| Perirenal haematoma | 3 | 171497 | 276 | 28308187 | 0.68 | -1.61 | 2.97 | 1.61 | 0.52 | 5.03 |
| Portal hypertensive gastropathy | 3 | 171497 | 230 | 28308233 | 0.88 | -1.41 | 3.17 | 1.85 | 0.59 | 5.77 |
| Postural orthostatic tachycardia syndrome | 3 | 171497 | 910 | 28307553 | -0.78 | -3.07 | 1.51 | 0.58 | 0.19 | 1.81 |
| Pulmonary infarction | 3 | 171497 | 1053 | 28307410 | -0.97 | -3.26 | 1.32 | 0.51 | 0.16 | 1.58 |
| Pulmonary veno-occlusive disease | 3 | 171497 | 632 | 28307831 | -0.30 | -2.60 | 1.99 | 0.81 | 0.26 | 2.51 |
| Renal arteriosclerosis | 3 | 171497 | 42 | 28308421 | 2.18 | -0.11 | 4.47 | 4.64 | 1.44 | 14.97 |
| Renal haematoma | 3 | 171497 | 679 | 28307784 | -0.40 | -2.69 | 1.89 | 0.76 | 0.24 | 2.36 |
| Renal vasculitis | 3 | 171497 | 168 | 28308295 | 1.19 | -1.10 | 3.48 | 2.31 | 0.74 | 7.22 |
| Reversible cerebral vasoconstriction syndrome | 3 | 171497 | 1299 | 28307164 | -1.25 | -3.54 | 1.04 | 0.42 | 0.13 | 1.30 |
| Sinoatrial block | 3 | 171497 | 466 | 28307997 | 0.07 | -2.22 | 2.36 | 1.05 | 0.34 | 3.28 |
| Soft tissue necrosis | 3 | 171497 | 439 | 28308024 | 0.15 | -2.14 | 2.44 | 1.11 | 0.36 | 3.45 |
| Splenic haematoma | 3 | 171497 | 295 | 28308168 | 0.61 | -1.68 | 2.90 | 1.53 | 0.49 | 4.77 |
| Splenic infarction | 3 | 171497 | 1157 | 28307306 | -1.10 | -3.39 | 1.19 | 0.47 | 0.15 | 1.45 |
| Splenic vein thrombosis | 3 | 171497 | 198 | 28308265 | 1.03 | -1.26 | 3.32 | 2.06 | 0.66 | 6.44 |
| Thrombotic stroke | 3 | 171497 | 610 | 28307853 | -0.26 | -2.55 | 2.03 | 0.83 | 0.27 | 2.59 |
| Tongue haemorrhage | 3 | 171497 | 715 | 28307748 | -0.46 | -2.75 | 1.83 | 0.72 | 0.23 | 2.25 |
| Tooth socket haemorrhage | 3 | 171497 | 240 | 28308223 | 0.83 | -1.46 | 3.12 | 1.79 | 0.57 | 5.59 |
| Vascular calcification | 3 | 171497 | 413 | 28308050 | 0.22 | -2.07 | 2.51 | 1.17 | 0.37 | 3.63 |
| Vascular dementia | 3 | 171497 | 904 | 28307559 | -0.77 | -3.06 | 1.52 | 0.59 | 0.19 | 1.82 |
| Vein discolouration | 3 | 171497 | 283 | 28308180 | 0.66 | -1.63 | 2.95 | 1.58 | 0.51 | 4.93 |
| Ventricular asystole | 3 | 171497 | 208 | 28308255 | 0.98 | -1.31 | 3.27 | 1.99 | 0.64 | 6.22 |
| Vertebrobasilar insufficiency | 3 | 171497 | 145 | 28308318 | 1.33 | -0.96 | 3.62 | 2.54 | 0.81 | 7.97 |
| White coat hypertension | 3 | 171497 | 159 | 28308304 | 1.25 | -1.04 | 3.54 | 2.39 | 0.76 | 7.50 |
| Wolff-parkinson-white syndrome | 3 | 171497 | 467 | 28307996 | 0.07 | -2.22 | 2.36 | 1.05 | 0.34 | 3.27 |

**Table S5.** Disproportionality results of cardiac and vascular toxicities associated with rabeprazole

| PT | a | b | c | d | IC | IC_025_ | IC_975_ | ROR | ROR_025_ | ROR_975_ |
| --- | --- | --- | --- | --- | --- | --- | --- | --- | --- | --- |
| Dyspnoea | 78 | 20017 | 218071 | 28241797 | -0.98 | -1.35 | -0.60 | 0.51 | 0.41 | 0.63 |
| Myocardial infarction | 72 | 20023 | 96258 | 28363610 | 0.08 | -0.31 | 0.47 | 1.06 | 0.84 | 1.34 |
| Peripheral swelling | 63 | 20032 | 104528 | 28355340 | -0.23 | -0.65 | 0.19 | 0.85 | 0.67 | 1.09 |
| Osteonecrosis of jaw | 55 | 20040 | 26568 | 28433300 | 1.53 | 1.08 | 1.97 | 2.89 | 2.21 | 3.76 |
| Dizziness | 54 | 20041 | 172782 | 28287086 | -1.17 | -1.62 | -0.71 | 0.44 | 0.34 | 0.58 |
| Oedema peripheral | 53 | 20042 | 50342 | 28409526 | 0.57 | 0.11 | 1.03 | 1.49 | 1.13 | 1.95 |
| Cerebral infarction | 50 | 20045 | 16117 | 28443751 | 2.08 | 1.61 | 2.56 | 4.26 | 3.23 | 5.62 |
| Syncope | 50 | 20045 | 75265 | 28384603 | -0.09 | -0.56 | 0.39 | 0.94 | 0.71 | 1.24 |
| Septic shock | 48 | 20047 | 38343 | 28421525 | 0.81 | 0.33 | 1.30 | 1.76 | 1.33 | 2.34 |
| Hypertension | 41 | 20054 | 88123 | 28371745 | -0.60 | -1.12 | -0.07 | 0.66 | 0.49 | 0.90 |
| Gastrointestinal haemorrhage | 40 | 20055 | 118729 | 28341139 | -1.06 | -1.59 | -0.53 | 0.48 | 0.35 | 0.65 |
| Chest pain | 37 | 20058 | 67974 | 28391894 | -0.37 | -0.92 | 0.18 | 0.77 | 0.56 | 1.07 |
| Hypotension | 33 | 20062 | 87435 | 28372433 | -0.89 | -1.48 | -0.31 | 0.54 | 0.38 | 0.76 |
| Pulmonary embolism | 33 | 20062 | 70042 | 28389826 | -0.58 | -1.16 | 0.01 | 0.67 | 0.48 | 0.94 |
| Right ventricular failure | 33 | 20062 | 7393 | 28452475 | 2.55 | 1.96 | 3.13 | 5.86 | 4.16 | 8.26 |
| Palpitations | 31 | 20064 | 52738 | 28407130 | -0.26 | -0.86 | 0.34 | 0.83 | 0.59 | 1.19 |
| Subdural haematoma | 28 | 20067 | 20660 | 28439208 | 0.92 | 0.28 | 1.55 | 1.89 | 1.30 | 2.74 |
| Haematemesis | 26 | 20069 | 12406 | 28447462 | 1.52 | 0.85 | 2.18 | 2.86 | 1.95 | 4.21 |
| Cardiac failure | 24 | 20071 | 38358 | 28421510 | -0.17 | -0.86 | 0.52 | 0.89 | 0.60 | 1.33 |
| Migraine | 23 | 20072 | 42560 | 28417308 | -0.38 | -1.08 | 0.33 | 0.77 | 0.51 | 1.16 |
| Pulmonary hypertension | 22 | 20073 | 14390 | 28445478 | 1.08 | 0.35 | 1.80 | 2.11 | 1.39 | 3.21 |
| Vasculitis | 22 | 20073 | 7339 | 28452529 | 1.98 | 1.26 | 2.71 | 3.96 | 2.61 | 6.02 |
| Flushing | 21 | 20074 | 30344 | 28429524 | -0.03 | -0.77 | 0.71 | 0.98 | 0.64 | 1.50 |
| Melaena | 21 | 20074 | 15290 | 28444578 | 0.93 | 0.19 | 1.67 | 1.90 | 1.24 | 2.92 |
| Pulmonary arterial hypertension | 21 | 20074 | 13871 | 28445997 | 1.06 | 0.32 | 1.80 | 2.09 | 1.36 | 3.21 |
| Pulmonary oedema | 20 | 20075 | 27763 | 28432105 | 0.03 | -0.73 | 0.79 | 1.02 | 0.66 | 1.58 |
| Thrombosis | 20 | 20075 | 63660 | 28396208 | -1.15 | -1.91 | -0.39 | 0.45 | 0.29 | 0.70 |
| Haemorrhage | 19 | 20076 | 44672 | 28415196 | -0.72 | -1.50 | 0.07 | 0.61 | 0.39 | 0.95 |
| Rectal haemorrhage | 19 | 20076 | 47983 | 28411885 | -0.82 | -1.60 | -0.04 | 0.57 | 0.36 | 0.89 |
| Tachycardia | 19 | 20076 | 44628 | 28415240 | -0.71 | -1.50 | 0.07 | 0.61 | 0.39 | 0.95 |
| Upper gastrointestinal haemorrhage | 19 | 20076 | 38508 | 28421360 | -0.51 | -1.29 | 0.28 | 0.70 | 0.45 | 1.10 |
| Cerebrovascular accident | 18 | 20077 | 78108 | 28381760 | -1.59 | -2.39 | -0.78 | 0.33 | 0.21 | 0.53 |
| Skin ulcer | 18 | 20077 | 18295 | 28441573 | 0.46 | -0.34 | 1.27 | 1.38 | 0.87 | 2.19 |
| Haematochezia | 16 | 20079 | 24453 | 28435415 | -0.11 | -0.96 | 0.75 | 0.93 | 0.57 | 1.52 |
| Small intestinal haemorrhage | 16 | 20079 | 2731 | 28457137 | 2.76 | 1.90 | 3.61 | 6.80 | 4.16 | 11.12 |
| Transient ischaemic attack | 16 | 20079 | 30376 | 28429492 | -0.41 | -1.27 | 0.44 | 0.75 | 0.46 | 1.23 |
| Anaphylactic reaction | 15 | 20080 | 25673 | 28434195 | -0.26 | -1.15 | 0.62 | 0.83 | 0.50 | 1.38 |
| Epistaxis | 15 | 20080 | 40046 | 28419822 | -0.89 | -1.78 | -0.01 | 0.54 | 0.32 | 0.89 |
| Ventricular fibrillation | 15 | 20080 | 8425 | 28451443 | 1.26 | 0.38 | 2.15 | 2.40 | 1.45 | 3.99 |
| Chest discomfort | 14 | 20081 | 32178 | 28427690 | -0.68 | -1.60 | 0.24 | 0.62 | 0.37 | 1.05 |
| Enterocolitis haemorrhagic | 14 | 20081 | 961 | 28458907 | 3.61 | 2.69 | 4.53 | 12.31 | 7.26 | 20.86 |
| Osteonecrosis | 14 | 20081 | 9630 | 28450238 | 0.99 | 0.07 | 1.91 | 1.99 | 1.18 | 3.36 |
| Shock haemorrhagic | 14 | 20081 | 9164 | 28450704 | 1.06 | 0.14 | 1.98 | 2.08 | 1.23 | 3.52 |
| Supraventricular tachycardia | 14 | 20081 | 7833 | 28452035 | 1.26 | 0.34 | 2.18 | 2.41 | 1.42 | 4.06 |
| Atrial fibrillation | 13 | 20082 | 45064 | 28414804 | -1.26 | -2.22 | -0.30 | 0.42 | 0.24 | 0.72 |
| Cardiac failure acute | 13 | 20082 | 4226 | 28455642 | 1.95 | 0.99 | 2.91 | 3.88 | 2.25 | 6.68 |
| Cerebral haemorrhage | 13 | 20082 | 24035 | 28435833 | -0.37 | -1.33 | 0.59 | 0.77 | 0.45 | 1.33 |
| Haematuria | 13 | 20082 | 23253 | 28436615 | -0.33 | -1.28 | 0.63 | 0.80 | 0.46 | 1.37 |
| Orthostatic hypotension | 13 | 20082 | 12511 | 28447357 | 0.53 | -0.43 | 1.49 | 1.45 | 0.84 | 2.49 |
| Pericardial effusion | 13 | 20082 | 12273 | 28447595 | 0.56 | -0.40 | 1.52 | 1.47 | 0.86 | 2.54 |
| Anaphylactic shock | 12 | 20083 | 13039 | 28446829 | 0.36 | -0.64 | 1.37 | 1.29 | 0.73 | 2.27 |
| Aortic dissection | 12 | 20083 | 1751 | 28458117 | 2.84 | 1.84 | 3.84 | 7.20 | 4.08 | 12.71 |
| Deep vein thrombosis | 12 | 20083 | 33234 | 28426634 | -0.94 | -1.94 | 0.06 | 0.52 | 0.30 | 0.92 |
| Hot flush | 12 | 20083 | 28017 | 28431851 | -0.70 | -1.70 | 0.30 | 0.62 | 0.35 | 1.09 |
| Ischaemic stroke | 12 | 20083 | 18224 | 28441644 | -0.10 | -1.10 | 0.90 | 0.94 | 0.53 | 1.65 |
| Ventricular tachycardia | 12 | 20083 | 12791 | 28447077 | 0.39 | -0.61 | 1.39 | 1.31 | 0.74 | 2.31 |
| Acute myocardial infarction | 11 | 20084 | 21921 | 28437947 | -0.47 | -1.52 | 0.58 | 0.72 | 0.40 | 1.30 |
| Arrhythmia | 11 | 20084 | 17107 | 28442761 | -0.13 | -1.18 | 0.92 | 0.91 | 0.51 | 1.65 |
| Cardiac arrest | 11 | 20084 | 31401 | 28428467 | -0.98 | -2.03 | 0.07 | 0.51 | 0.28 | 0.92 |
| Embolic stroke | 11 | 20084 | 3369 | 28456499 | 2.00 | 0.95 | 3.04 | 4.00 | 2.21 | 7.22 |
| Haematoma | 11 | 20084 | 14742 | 28445126 | 0.08 | -0.97 | 1.13 | 1.05 | 0.58 | 1.90 |
| Subarachnoid haemorrhage | 11 | 20084 | 10162 | 28449706 | 0.58 | -0.47 | 1.63 | 1.50 | 0.83 | 2.71 |
| Sudden death | 11 | 20084 | 11087 | 28448781 | 0.47 | -0.58 | 1.51 | 1.38 | 0.76 | 2.49 |
| Ventricular extrasystoles | 11 | 20084 | 6322 | 28453546 | 1.21 | 0.16 | 2.26 | 2.32 | 1.28 | 4.19 |
| Cardiac failure congestive | 10 | 20085 | 43168 | 28416700 | -1.56 | -2.67 | -0.45 | 0.34 | 0.18 | 0.63 |
| Injection site bruising | 10 | 20085 | 29560 | 28430308 | -1.02 | -2.13 | 0.08 | 0.49 | 0.26 | 0.91 |
| Pericarditis | 10 | 20085 | 6168 | 28453700 | 1.11 | 0.00 | 2.22 | 2.16 | 1.16 | 4.02 |
| Sinus bradycardia | 10 | 20085 | 6222 | 28453646 | 1.10 | -0.01 | 2.21 | 2.15 | 1.15 | 3.99 |
| Subcutaneous haematoma | 10 | 20085 | 1583 | 28458285 | 2.69 | 1.59 | 3.80 | 6.49 | 3.49 | 12.09 |
| Angina pectoris | 9 | 20086 | 12461 | 28447407 | 0.03 | -1.14 | 1.20 | 1.02 | 0.53 | 1.96 |
| Ascites | 9 | 20086 | 10758 | 28449110 | 0.23 | -0.94 | 1.40 | 1.17 | 0.61 | 2.26 |
| Cardiac failure chronic | 9 | 20086 | 2080 | 28457788 | 2.27 | 1.09 | 3.44 | 4.83 | 2.51 | 9.29 |
| Lacunar infarction | 9 | 20086 | 1716 | 28458152 | 2.47 | 1.29 | 3.64 | 5.55 | 2.88 | 10.69 |
| Renal haemangioma | 9 | 20086 | 2073 | 28457795 | 2.27 | 1.10 | 3.44 | 4.84 | 2.51 | 9.32 |
| Thalamus haemorrhage | 9 | 20086 | 1707 | 28458161 | 2.47 | 1.30 | 3.65 | 5.57 | 2.89 | 10.73 |
| Thrombotic cerebral infarction | 9 | 20086 | 892 | 28458976 | 3.06 | 1.89 | 4.24 | 8.41 | 4.36 | 16.22 |
| Thrombotic microangiopathy | 9 | 20086 | 7798 | 28452070 | 0.66 | -0.51 | 1.83 | 1.58 | 0.82 | 3.04 |
| Cardiac disorder | 8 | 20087 | 32375 | 28427493 | -1.46 | -2.71 | -0.20 | 0.36 | 0.18 | 0.73 |
| Dyspnoea exertional | 8 | 20087 | 15035 | 28444833 | -0.39 | -1.64 | 0.87 | 0.76 | 0.38 | 1.53 |
| Injection site haemorrhage | 8 | 20087 | 24898 | 28434970 | -1.09 | -2.34 | 0.17 | 0.47 | 0.23 | 0.94 |
| Peripheral arterial occlusive disease | 8 | 20087 | 3228 | 28456640 | 1.61 | 0.36 | 2.87 | 3.06 | 1.53 | 6.12 |
| Poor venous access | 8 | 20087 | 3197 | 28456671 | 1.62 | 0.37 | 2.88 | 3.08 | 1.54 | 6.17 |
| Presyncope | 8 | 20087 | 15153 | 28444715 | -0.40 | -1.65 | 0.86 | 0.76 | 0.38 | 1.52 |
| Bradycardia | 7 | 20088 | 19810 | 28440058 | -0.95 | -2.30 | 0.41 | 0.52 | 0.25 | 1.09 |
| Haemorrhagic diathesis | 7 | 20088 | 1800 | 28458068 | 2.08 | 0.72 | 3.43 | 4.24 | 2.02 | 8.90 |
| Lower gastrointestinal haemorrhage | 7 | 20088 | 17909 | 28441959 | -0.81 | -2.16 | 0.55 | 0.57 | 0.27 | 1.20 |
| Phlebitis | 7 | 20088 | 2830 | 28457038 | 1.58 | 0.23 | 2.94 | 3.00 | 1.43 | 6.31 |
| Pulmonary alveolar haemorrhage | 7 | 20088 | 3975 | 28455893 | 1.18 | -0.18 | 2.54 | 2.27 | 1.08 | 4.76 |
| Pulmonary haemorrhage | 7 | 20088 | 5669 | 28454199 | 0.74 | -0.62 | 2.09 | 1.67 | 0.79 | 3.50 |
| Shock | 7 | 20088 | 10776 | 28449092 | -0.11 | -1.47 | 1.24 | 0.92 | 0.44 | 1.94 |
| Stress cardiomyopathy | 7 | 20088 | 4872 | 28454996 | 0.93 | -0.43 | 2.28 | 1.90 | 0.91 | 4.00 |
| Cardio-respiratory arrest | 6 | 20089 | 17223 | 28442645 | -0.96 | -2.44 | 0.52 | 0.51 | 0.23 | 1.14 |
| Colitis ischaemic | 6 | 20089 | 3836 | 28456032 | 1.02 | -0.47 | 2.50 | 2.03 | 0.91 | 4.51 |
| Contusion | 6 | 20089 | 35332 | 28424536 | -1.97 | -3.45 | -0.48 | 0.26 | 0.11 | 0.57 |
| Coronary artery disease | 6 | 20089 | 13795 | 28446073 | -0.66 | -2.14 | 0.83 | 0.63 | 0.29 | 1.41 |
| Disseminated intravascular coagulation | 6 | 20089 | 5072 | 28454796 | 0.67 | -0.81 | 2.15 | 1.59 | 0.72 | 3.55 |
| Gastric ulcer haemorrhage | 6 | 20089 | 4894 | 28454974 | 0.72 | -0.77 | 2.20 | 1.64 | 0.74 | 3.66 |
| Pulmonary congestion | 6 | 20089 | 6241 | 28453627 | 0.41 | -1.08 | 1.89 | 1.32 | 0.59 | 2.95 |
| Thrombotic thrombocytopenic purpura | 6 | 20089 | 2938 | 28456930 | 1.33 | -0.15 | 2.82 | 2.53 | 1.13 | 5.63 |
| Venous thrombosis limb | 6 | 20089 | 2762 | 28457106 | 1.41 | -0.08 | 2.89 | 2.65 | 1.19 | 5.91 |
| Visceral congestion | 6 | 20089 | 377 | 28459491 | 3.08 | 1.59 | 4.56 | 8.48 | 3.79 | 19.01 |
| Cerebellar haemorrhage | 5 | 20090 | 1403 | 28458465 | 1.88 | 0.23 | 3.54 | 3.69 | 1.53 | 8.88 |
| Hypertensive crisis | 5 | 20090 | 8077 | 28451791 | -0.17 | -1.83 | 1.48 | 0.89 | 0.37 | 2.13 |
| Hypovolaemic shock | 5 | 20090 | 2722 | 28457146 | 1.18 | -0.47 | 2.84 | 2.27 | 0.94 | 5.46 |
| Internal haemorrhage | 5 | 20090 | 19701 | 28440167 | -1.39 | -3.04 | 0.27 | 0.38 | 0.16 | 0.92 |
| Oesophageal varices haemorrhage | 5 | 20090 | 2160 | 28457708 | 1.44 | -0.22 | 3.09 | 2.72 | 1.13 | 6.53 |
| Portal vein thrombosis | 5 | 20090 | 1996 | 28457872 | 1.52 | -0.13 | 3.18 | 2.88 | 1.20 | 6.93 |
| Prinzmetal angina | 5 | 20090 | 1079 | 28458789 | 2.12 | 0.47 | 3.78 | 4.36 | 1.81 | 10.50 |
| Purpura | 5 | 20090 | 4377 | 28455491 | 0.61 | -1.04 | 2.27 | 1.53 | 0.64 | 3.68 |
| Putamen haemorrhage | 5 | 20090 | 571 | 28459297 | 2.60 | 0.95 | 4.26 | 6.09 | 2.52 | 14.69 |
| Retinal vein occlusion | 5 | 20090 | 1901 | 28457967 | 1.58 | -0.08 | 3.23 | 2.99 | 1.24 | 7.18 |
| Sinus node dysfunction | 5 | 20090 | 2856 | 28457012 | 1.13 | -0.53 | 2.78 | 2.19 | 0.91 | 5.26 |
| Sinus tachycardia | 5 | 20090 | 6683 | 28453185 | 0.08 | -1.58 | 1.73 | 1.05 | 0.44 | 2.53 |
| Brain stem infarction | 4 | 20091 | 699 | 28459169 | 2.18 | 0.28 | 4.08 | 4.53 | 1.70 | 12.10 |
| Cardiac tamponade | 4 | 20091 | 2732 | 28457136 | 0.89 | -1.01 | 2.79 | 1.85 | 0.69 | 4.94 |
| Haemoptysis | 4 | 20091 | 18477 | 28441391 | -1.59 | -3.49 | 0.31 | 0.33 | 0.12 | 0.88 |
| Haemorrhoidal haemorrhage | 4 | 20091 | 4588 | 28455280 | 0.27 | -1.63 | 2.17 | 1.20 | 0.45 | 3.21 |
| Haemorrhoids | 4 | 20091 | 8815 | 28451053 | -0.58 | -2.48 | 1.32 | 0.67 | 0.25 | 1.78 |
| Mitral valve prolapse | 4 | 20091 | 770 | 28459098 | 2.10 | 0.20 | 4.00 | 4.31 | 1.61 | 11.52 |
| Pallor | 4 | 20091 | 8788 | 28451080 | -0.57 | -2.47 | 1.33 | 0.67 | 0.25 | 1.79 |
| Peripheral coldness | 4 | 20091 | 6070 | 28453798 | -0.09 | -1.99 | 1.81 | 0.94 | 0.35 | 2.51 |
| Renal cortical necrosis | 4 | 20091 | 109 | 28459759 | 2.96 | 1.06 | 4.86 | 7.80 | 2.88 | 21.16 |
| Retinal haemorrhage | 4 | 20091 | 4026 | 28455842 | 0.43 | -1.47 | 2.33 | 1.35 | 0.50 | 3.59 |
| Sudden cardiac death | 4 | 20091 | 2817 | 28457051 | 0.85 | -1.05 | 2.75 | 1.81 | 0.68 | 4.82 |
| Vitreous haemorrhage | 4 | 20091 | 2662 | 28457206 | 0.92 | -0.98 | 2.82 | 1.89 | 0.71 | 5.04 |
| Aortic aneurysm rupture | 3 | 20092 | 729 | 28459139 | 1.78 | -0.51 | 4.07 | 3.45 | 1.11 | 10.72 |
| Arteriosclerosis | 3 | 20092 | 2672 | 28457196 | 0.55 | -1.74 | 2.84 | 1.47 | 0.47 | 4.55 |
| Atrioventricular block complete | 3 | 20092 | 3191 | 28456677 | 0.35 | -1.94 | 2.64 | 1.27 | 0.41 | 3.94 |
| Basal ganglia haemorrhage | 3 | 20092 | 836 | 28459032 | 1.68 | -0.61 | 3.97 | 3.21 | 1.03 | 9.98 |
| Cardiogenic shock | 3 | 20092 | 4842 | 28455026 | -0.16 | -2.45 | 2.13 | 0.89 | 0.29 | 2.77 |
| Cardiomyopathy | 3 | 20092 | 6252 | 28453616 | -0.49 | -2.78 | 1.80 | 0.71 | 0.23 | 2.21 |
| Cardiovascular disorder | 3 | 20092 | 6172 | 28453696 | -0.47 | -2.76 | 1.82 | 0.72 | 0.23 | 2.23 |
| Dizziness postural | 3 | 20092 | 3476 | 28456392 | 0.24 | -2.05 | 2.53 | 1.18 | 0.38 | 3.68 |
| Fluid overload | 3 | 20092 | 10332 | 28449536 | -1.15 | -3.44 | 1.14 | 0.45 | 0.14 | 1.39 |
| Gastric antral vascular ectasia | 3 | 20092 | 273 | 28459595 | 2.33 | 0.04 | 4.62 | 5.05 | 1.62 | 15.76 |
| Haemophilic arthropathy | 3 | 20092 | 144 | 28459724 | 2.54 | 0.25 | 4.83 | 5.82 | 1.85 | 18.25 |
| Haemorrhage intracranial | 3 | 20092 | 13833 | 28446035 | -1.55 | -3.84 | 0.74 | 0.34 | 0.11 | 1.06 |
| Hypoxic-ischaemic encephalopathy | 3 | 20092 | 1227 | 28458641 | 1.36 | -0.93 | 3.65 | 2.56 | 0.82 | 7.95 |
| Injection site haematoma | 3 | 20092 | 1430 | 28458438 | 1.21 | -1.08 | 3.50 | 2.32 | 0.75 | 7.20 |
| Intra-abdominal haemorrhage | 3 | 20092 | 1469 | 28458399 | 1.19 | -1.10 | 3.48 | 2.28 | 0.73 | 7.07 |
| Mouth haemorrhage | 3 | 20092 | 3875 | 28455993 | 0.11 | -2.18 | 2.40 | 1.08 | 0.35 | 3.36 |
| Muscle haemorrhage | 3 | 20092 | 3095 | 28456773 | 0.38 | -1.91 | 2.67 | 1.30 | 0.42 | 4.04 |
| Myocarditis | 3 | 20092 | 5770 | 28454098 | -0.39 | -2.68 | 1.90 | 0.77 | 0.25 | 2.37 |
| Nephroangiosclerosis | 3 | 20092 | 93 | 28459775 | 2.62 | 0.33 | 4.91 | 6.19 | 1.96 | 19.54 |
| Oesophagitis haemorrhagic | 3 | 20092 | 565 | 28459303 | 1.96 | -0.33 | 4.25 | 3.89 | 1.25 | 12.11 |
| Periorbital haematoma | 3 | 20092 | 341 | 28459527 | 2.24 | -0.05 | 4.53 | 4.72 | 1.52 | 14.72 |
| Peripheral venous disease | 3 | 20092 | 1337 | 28458531 | 1.28 | -1.01 | 3.57 | 2.42 | 0.78 | 7.53 |
| Pulmonary thrombosis | 3 | 20092 | 7502 | 28452366 | -0.73 | -3.02 | 1.56 | 0.60 | 0.19 | 1.87 |
| Retroperitoneal haemorrhage | 3 | 20092 | 1954 | 28457914 | 0.90 | -1.39 | 3.19 | 1.86 | 0.60 | 5.78 |
| Splenic infarction | 3 | 20092 | 1157 | 28458711 | 1.41 | -0.88 | 3.70 | 2.66 | 0.86 | 8.25 |
| Stoma site haemorrhage | 3 | 20092 | 1395 | 28458473 | 1.24 | -1.05 | 3.53 | 2.36 | 0.76 | 7.32 |
| Strangulated hernia | 3 | 20092 | 264 | 28459604 | 2.35 | 0.06 | 4.64 | 5.10 | 1.63 | 15.91 |
| Thrombophlebitis | 3 | 20092 | 2950 | 28456918 | 0.44 | -1.85 | 2.73 | 1.36 | 0.44 | 4.20 |
| Torsade de pointes | 3 | 20092 | 5461 | 28454407 | -0.32 | -2.61 | 1.97 | 0.80 | 0.26 | 2.49 |
| Traumatic haematoma | 3 | 20092 | 1173 | 28458695 | 1.40 | -0.89 | 3.69 | 2.64 | 0.85 | 8.18 |
| Ulcer haemorrhage | 3 | 20092 | 5948 | 28453920 | -0.42 | -2.72 | 1.87 | 0.74 | 0.24 | 2.31 |
| Uterine haemorrhage | 3 | 20092 | 3026 | 28456842 | 0.41 | -1.88 | 2.70 | 1.33 | 0.43 | 4.12 |
| Vaginal haemorrhage | 3 | 20092 | 27004 | 28432864 | -2.48 | -4.77 | -0.19 | 0.18 | 0.06 | 0.55 |
| Varices oesophageal | 3 | 20092 | 2041 | 28457827 | 0.85 | -1.44 | 3.14 | 1.80 | 0.58 | 5.60 |

**Table S6**. Disproportionality results of cardiac and vascular toxicities associated with esomeprazole

| PT | a | b | c | d | IC | IC_025_ | IC_975_ | ROR | ROR_025_ | ROR_975_ |
| --- | --- | --- | --- | --- | --- | --- | --- | --- | --- | --- |
| Dyspnoea | 132 | 26883 | 218017 | 28234931 | -0.65 | -0.94 | -0.36 | 0.64 | 0.54 | 0.76 |
| Hypotension | 110 | 26905 | 87358 | 28365590 | 0.40 | 0.09 | 0.72 | 1.33 | 1.10 | 1.60 |
| Hypertension | 85 | 26930 | 88079 | 28364869 | 0.02 | -0.34 | 0.38 | 1.02 | 0.82 | 1.26 |
| Septic shock | 84 | 26931 | 38307 | 28414641 | 1.19 | 0.83 | 1.56 | 2.30 | 1.85 | 2.84 |
| Syncope | 82 | 26933 | 75233 | 28377715 | 0.20 | -0.17 | 0.56 | 1.15 | 0.92 | 1.43 |
| Dizziness | 81 | 26934 | 172755 | 28280193 | -1.01 | -1.38 | -0.64 | 0.49 | 0.40 | 0.61 |
| Pulmonary embolism | 61 | 26954 | 70014 | 28382934 | -0.12 | -0.55 | 0.30 | 0.92 | 0.71 | 1.18 |
| Rectal haemorrhage | 58 | 26957 | 47944 | 28405004 | 0.35 | -0.09 | 0.78 | 1.27 | 0.98 | 1.65 |
| Peripheral swelling | 54 | 26961 | 104537 | 28348411 | -0.87 | -1.33 | -0.42 | 0.55 | 0.42 | 0.71 |
| Subdural haematoma | 54 | 26961 | 20634 | 28432314 | 1.44 | 0.98 | 1.89 | 2.72 | 2.08 | 3.55 |
| Chest pain | 51 | 26964 | 67960 | 28384988 | -0.34 | -0.80 | 0.13 | 0.79 | 0.60 | 1.04 |
| Oedema peripheral | 46 | 26969 | 50349 | 28402599 | -0.05 | -0.55 | 0.44 | 0.96 | 0.72 | 1.29 |
| Gastrointestinal haemorrhage | 44 | 26971 | 118725 | 28334223 | -1.35 | -1.85 | -0.84 | 0.39 | 0.29 | 0.53 |
| Haematuria | 44 | 26971 | 23222 | 28429726 | 0.98 | 0.48 | 1.48 | 1.98 | 1.47 | 2.66 |
| Palpitations | 44 | 26971 | 52725 | 28400223 | -0.18 | -0.69 | 0.32 | 0.88 | 0.65 | 1.18 |
| Upper gastrointestinal haemorrhage | 41 | 26974 | 38486 | 28414462 | 0.16 | -0.36 | 0.69 | 1.12 | 0.82 | 1.52 |
| Pericarditis | 40 | 26975 | 6138 | 28446810 | 2.67 | 2.14 | 3.20 | 6.41 | 4.69 | 8.75 |
| Chest discomfort | 39 | 26976 | 32153 | 28420795 | 0.35 | -0.19 | 0.88 | 1.27 | 0.93 | 1.74 |
| Tachycardia | 39 | 26976 | 44608 | 28408340 | -0.12 | -0.65 | 0.42 | 0.92 | 0.67 | 1.26 |
| Thrombosis | 38 | 26977 | 63642 | 28389306 | -0.66 | -1.21 | -0.12 | 0.63 | 0.46 | 0.87 |
| Transient ischaemic attack | 37 | 26978 | 30355 | 28422593 | 0.35 | -0.20 | 0.91 | 1.28 | 0.93 | 1.77 |
| Cardiac failure | 34 | 26981 | 38348 | 28414600 | -0.10 | -0.67 | 0.48 | 0.93 | 0.67 | 1.31 |
| Myocardial infarction | 34 | 26981 | 96296 | 28356652 | -1.41 | -1.99 | -0.84 | 0.37 | 0.27 | 0.52 |
| Anaphylactic reaction | 33 | 26982 | 25655 | 28427293 | 0.43 | -0.16 | 1.01 | 1.35 | 0.96 | 1.90 |
| Atrial fibrillation | 32 | 26983 | 45045 | 28407903 | -0.41 | -1.01 | 0.18 | 0.75 | 0.53 | 1.06 |
| Melaena | 32 | 26983 | 15279 | 28437669 | 1.11 | 0.52 | 1.71 | 2.17 | 1.53 | 3.07 |
| Cardio-respiratory arrest | 28 | 26987 | 17201 | 28435747 | 0.76 | 0.12 | 1.40 | 1.69 | 1.17 | 2.45 |
| Cerebrovascular accident | 27 | 26988 | 78099 | 28374849 | -1.44 | -2.09 | -0.79 | 0.37 | 0.25 | 0.54 |
| Haemorrhage | 27 | 26988 | 44664 | 28408284 | -0.64 | -1.29 | 0.01 | 0.64 | 0.44 | 0.93 |
| Ischaemic stroke | 27 | 26988 | 18209 | 28434739 | 0.63 | -0.02 | 1.28 | 1.55 | 1.06 | 2.26 |
| Sudden death | 27 | 26988 | 11071 | 28441877 | 1.32 | 0.67 | 1.97 | 2.50 | 1.71 | 3.65 |
| Deep vein thrombosis | 26 | 26989 | 33220 | 28419728 | -0.27 | -0.94 | 0.39 | 0.83 | 0.56 | 1.21 |
| Haematoma | 26 | 26989 | 14727 | 28438221 | 0.87 | 0.21 | 1.53 | 1.83 | 1.25 | 2.69 |
| Orthostatic hypotension | 25 | 26990 | 12499 | 28440449 | 1.04 | 0.37 | 1.72 | 2.06 | 1.39 | 3.05 |
| Skin ulcer | 25 | 26990 | 18288 | 28434660 | 0.51 | -0.16 | 1.19 | 1.43 | 0.96 | 2.11 |
| Hypertensive nephropathy | 24 | 26991 | 386 | 28452562 | 4.78 | 4.09 | 5.48 | 28.29 | 18.72 | 42.73 |
| Pulmonary oedema | 24 | 26991 | 27759 | 28425189 | -0.13 | -0.82 | 0.56 | 0.91 | 0.61 | 1.36 |
| Haemoptysis | 23 | 26992 | 18458 | 28434490 | 0.38 | -0.32 | 1.09 | 1.30 | 0.87 | 1.96 |
| Haematochezia | 20 | 26995 | 24449 | 28428499 | -0.21 | -0.97 | 0.55 | 0.86 | 0.56 | 1.34 |
| Migraine | 20 | 26995 | 42563 | 28410385 | -1.00 | -1.76 | -0.24 | 0.50 | 0.32 | 0.78 |
| Shock haemorrhagic | 20 | 26995 | 9158 | 28443790 | 1.15 | 0.39 | 1.92 | 2.23 | 1.44 | 3.46 |
| Bradycardia | 19 | 26996 | 19798 | 28433150 | 0.02 | -0.77 | 0.80 | 1.01 | 0.64 | 1.58 |
| Vascular purpura | 19 | 26996 | 1578 | 28451370 | 3.27 | 2.49 | 4.06 | 9.76 | 6.21 | 15.35 |
| Acute myocardial infarction | 18 | 26997 | 21914 | 28431034 | -0.20 | -1.01 | 0.60 | 0.87 | 0.55 | 1.38 |
| Epistaxis | 18 | 26997 | 40043 | 28412905 | -1.06 | -1.86 | -0.25 | 0.48 | 0.30 | 0.76 |
| Renal haemangioma | 18 | 26997 | 2064 | 28450884 | 2.90 | 2.10 | 3.71 | 7.52 | 4.73 | 11.97 |
| Shock | 18 | 26997 | 10765 | 28442183 | 0.79 | -0.02 | 1.59 | 1.73 | 1.09 | 2.74 |
| Osteonecrosis of jaw | 17 | 26998 | 26606 | 28426342 | -0.56 | -1.39 | 0.27 | 0.68 | 0.42 | 1.09 |
| Ventricular tachycardia | 17 | 26998 | 12786 | 28440162 | 0.47 | -0.36 | 1.30 | 1.38 | 0.86 | 2.23 |
| Arrhythmia | 16 | 26999 | 17102 | 28435846 | -0.02 | -0.88 | 0.84 | 0.99 | 0.60 | 1.61 |
| Muscle haemorrhage | 16 | 26999 | 3082 | 28449866 | 2.26 | 1.41 | 3.12 | 4.82 | 2.95 | 7.88 |
| Pericardial haemorrhage | 16 | 26999 | 3157 | 28449791 | 2.23 | 1.38 | 3.09 | 4.72 | 2.89 | 7.71 |
| Haematemesis | 15 | 27000 | 12417 | 28440531 | 0.33 | -0.55 | 1.22 | 1.26 | 0.76 | 2.09 |
| Pallor | 15 | 27000 | 8777 | 28444171 | 0.81 | -0.08 | 1.70 | 1.76 | 1.06 | 2.91 |
| Pulmonary hypertension | 15 | 27000 | 14397 | 28438551 | 0.13 | -0.76 | 1.02 | 1.09 | 0.66 | 1.82 |
| Angina pectoris | 14 | 27001 | 12456 | 28440492 | 0.23 | -0.69 | 1.15 | 1.18 | 0.70 | 1.99 |
| Cardiac failure congestive | 13 | 27002 | 43165 | 28409783 | -1.62 | -2.58 | -0.66 | 0.33 | 0.19 | 0.56 |
| Contusion | 13 | 27002 | 35325 | 28417623 | -1.33 | -2.29 | -0.38 | 0.40 | 0.23 | 0.68 |
| Hypertensive crisis | 13 | 27002 | 8069 | 28444879 | 0.73 | -0.23 | 1.68 | 1.65 | 0.96 | 2.85 |
| Supraventricular tachycardia | 13 | 27002 | 7834 | 28445114 | 0.77 | -0.19 | 1.72 | 1.70 | 0.99 | 2.93 |
| Torsade de pointes | 13 | 27002 | 5451 | 28447497 | 1.25 | 0.29 | 2.21 | 2.38 | 1.38 | 4.10 |
| Anaphylactic shock | 12 | 27003 | 13039 | 28439909 | -0.04 | -1.04 | 0.96 | 0.97 | 0.55 | 1.71 |
| Cardiac arrest | 12 | 27003 | 31400 | 28421548 | -1.28 | -2.28 | -0.28 | 0.41 | 0.23 | 0.73 |
| Cardiac disorder | 12 | 27003 | 32371 | 28420577 | -1.32 | -2.32 | -0.32 | 0.40 | 0.23 | 0.70 |
| Cerebral haemorrhage | 12 | 27003 | 24036 | 28428912 | -0.90 | -1.90 | 0.10 | 0.54 | 0.30 | 0.94 |
| Haemorrhagic stroke | 12 | 27003 | 10146 | 28442802 | 0.30 | -0.70 | 1.30 | 1.23 | 0.70 | 2.17 |
| Hot flush | 12 | 27003 | 28017 | 28424931 | -1.12 | -2.12 | -0.11 | 0.46 | 0.26 | 0.81 |
| Presyncope | 12 | 27003 | 15149 | 28437799 | -0.25 | -1.25 | 0.75 | 0.84 | 0.48 | 1.48 |
| Cerebral infarction | 11 | 27004 | 16156 | 28436792 | -0.46 | -1.51 | 0.59 | 0.73 | 0.40 | 1.31 |
| Extrasystoles | 11 | 27004 | 2606 | 28450342 | 1.95 | 0.90 | 3.00 | 3.87 | 2.14 | 6.99 |
| Jugular vein thrombosis | 11 | 27004 | 832 | 28452116 | 3.15 | 2.10 | 4.20 | 8.92 | 4.92 | 16.17 |
| Purple glove syndrome | 11 | 27004 | 224 | 28452724 | 3.99 | 2.94 | 5.04 | 16.14 | 8.81 | 29.57 |
| Renal infarct | 11 | 27004 | 729 | 28452219 | 3.26 | 2.21 | 4.31 | 9.65 | 5.32 | 17.50 |
| Sinus bradycardia | 11 | 27004 | 6221 | 28446727 | 0.84 | -0.21 | 1.89 | 1.80 | 0.99 | 3.24 |
| Subdural haemorrhage | 11 | 27004 | 3398 | 28449550 | 1.62 | 0.57 | 2.67 | 3.09 | 1.71 | 5.58 |
| Ventricular fibrillation | 11 | 27004 | 8429 | 28444519 | 0.44 | -0.61 | 1.48 | 1.35 | 0.75 | 2.44 |
| Colitis ischaemic | 10 | 27005 | 3832 | 28449116 | 1.34 | 0.23 | 2.45 | 2.54 | 1.36 | 4.72 |
| Dyspnoea exertional | 10 | 27005 | 15033 | 28437915 | -0.49 | -1.60 | 0.61 | 0.71 | 0.38 | 1.32 |
| Oesophageal varices haemorrhage | 10 | 27005 | 2155 | 28450793 | 2.04 | 0.93 | 3.15 | 4.12 | 2.22 | 7.68 |
| Pericardial effusion | 10 | 27005 | 12276 | 28440672 | -0.21 | -1.32 | 0.90 | 0.86 | 0.46 | 1.61 |
| Peripheral ischaemia | 10 | 27005 | 3050 | 28449898 | 1.63 | 0.52 | 2.73 | 3.09 | 1.66 | 5.75 |
| Renal artery stenosis | 10 | 27005 | 678 | 28452270 | 3.19 | 2.08 | 4.29 | 9.18 | 4.92 | 17.15 |
| Retroperitoneal haematoma | 10 | 27005 | 1890 | 28451058 | 2.19 | 1.08 | 3.30 | 4.58 | 2.46 | 8.52 |
| Subarachnoid haemorrhage | 10 | 27005 | 10163 | 28442785 | 0.05 | -1.06 | 1.16 | 1.03 | 0.56 | 1.92 |
| Acute coronary syndrome | 9 | 27006 | 4875 | 28448073 | 0.89 | -0.29 | 2.06 | 1.85 | 0.96 | 3.56 |
| Tumour haemorrhage | 9 | 27006 | 2586 | 28450362 | 1.68 | 0.51 | 2.86 | 3.22 | 1.67 | 6.19 |
| Cardiac flutter | 8 | 27007 | 2556 | 28450392 | 1.54 | 0.28 | 2.79 | 2.90 | 1.45 | 5.82 |
| Cardiomyopathy | 8 | 27007 | 6247 | 28446701 | 0.40 | -0.85 | 1.66 | 1.32 | 0.66 | 2.64 |
| Flushing | 8 | 27007 | 30357 | 28422591 | -1.79 | -3.04 | -0.53 | 0.29 | 0.14 | 0.58 |
| Myocarditis | 8 | 27007 | 5765 | 28447183 | 0.51 | -0.75 | 1.76 | 1.42 | 0.71 | 2.85 |
| Osteonecrosis | 8 | 27007 | 9636 | 28443312 | -0.18 | -1.44 | 1.07 | 0.88 | 0.44 | 1.76 |
| Pulmonary arterial hypertension | 8 | 27007 | 13884 | 28439064 | -0.69 | -1.94 | 0.57 | 0.62 | 0.31 | 1.24 |
| Waterhouse-friderichsen syndrome | 8 | 27007 | 159 | 28452789 | 3.69 | 2.44 | 4.95 | 13.06 | 6.42 | 26.57 |
| Blood pressure fluctuation | 7 | 27008 | 8370 | 28444578 | -0.17 | -1.53 | 1.18 | 0.89 | 0.42 | 1.86 |
| Cardiovascular disorder | 7 | 27008 | 6168 | 28446780 | 0.24 | -1.12 | 1.59 | 1.18 | 0.56 | 2.48 |
| Carotid artery thrombosis | 7 | 27008 | 233 | 28452715 | 3.37 | 2.01 | 4.72 | 10.40 | 4.90 | 22.06 |
| Coronary artery disease | 7 | 27008 | 13794 | 28439154 | -0.86 | -2.21 | 0.50 | 0.55 | 0.26 | 1.16 |
| Duodenal ulcer haemorrhage | 7 | 27008 | 2417 | 28450531 | 1.42 | 0.07 | 2.78 | 2.68 | 1.28 | 5.64 |
| Haemodynamic instability | 7 | 27008 | 2078 | 28450870 | 1.60 | 0.24 | 2.95 | 3.03 | 1.44 | 6.37 |
| Heart injury | 7 | 27008 | 554 | 28452394 | 2.86 | 1.51 | 4.22 | 7.31 | 3.47 | 15.41 |
| Heat stroke | 7 | 27008 | 780 | 28452168 | 2.59 | 1.23 | 3.94 | 6.05 | 2.87 | 12.73 |
| Infarction | 7 | 27008 | 3372 | 28449576 | 1.02 | -0.34 | 2.37 | 2.03 | 0.97 | 4.25 |
| Post procedural haemorrhage | 7 | 27008 | 7353 | 28445595 | 0.00 | -1.35 | 1.36 | 1.00 | 0.48 | 2.10 |
| Purpura | 7 | 27008 | 4375 | 28448573 | 0.69 | -0.67 | 2.04 | 1.61 | 0.77 | 3.38 |
| Sinus node dysfunction | 7 | 27008 | 2854 | 28450094 | 1.22 | -0.13 | 2.58 | 2.34 | 1.11 | 4.91 |
| Vaginal haemorrhage | 7 | 27008 | 27000 | 28425948 | -1.80 | -3.16 | -0.44 | 0.29 | 0.14 | 0.60 |
| Vasculitis | 7 | 27008 | 7354 | 28445594 | 0.00 | -1.35 | 1.36 | 1.00 | 0.48 | 2.10 |
| Angina unstable | 6 | 27009 | 4064 | 28448884 | 0.58 | -0.91 | 2.06 | 1.49 | 0.67 | 3.32 |
| Ascites | 6 | 27009 | 10761 | 28442187 | -0.72 | -2.20 | 0.76 | 0.61 | 0.27 | 1.35 |
| Injection site haemorrhage | 6 | 27009 | 24900 | 28428048 | -1.89 | -3.38 | -0.41 | 0.27 | 0.12 | 0.60 |
| Left ventricular dysfunction | 6 | 27009 | 4497 | 28448451 | 0.45 | -1.04 | 1.93 | 1.36 | 0.61 | 3.04 |
| Patent ductus arteriosus | 6 | 27009 | 1553 | 28451395 | 1.72 | 0.23 | 3.20 | 3.29 | 1.48 | 7.34 |
| Pulmonary haemorrhage | 6 | 27009 | 5670 | 28447278 | 0.14 | -1.34 | 1.63 | 1.10 | 0.50 | 2.46 |
| Sinus tachycardia | 6 | 27009 | 6682 | 28446266 | -0.07 | -1.56 | 1.41 | 0.95 | 0.43 | 2.11 |
| Thrombotic microangiopathy | 6 | 27009 | 7801 | 28445147 | -0.28 | -1.77 | 1.20 | 0.82 | 0.37 | 1.83 |
| Cerebral haematoma | 5 | 27010 | 2102 | 28450846 | 1.14 | -0.52 | 2.79 | 2.20 | 0.92 | 5.30 |
| Ecchymosis | 5 | 27010 | 2440 | 28450508 | 0.96 | -0.69 | 2.62 | 1.95 | 0.81 | 4.70 |
| Gastric antral vascular ectasia | 5 | 27010 | 271 | 28452677 | 2.85 | 1.20 | 4.51 | 7.26 | 3.00 | 17.59 |
| Internal haemorrhage | 5 | 27010 | 19701 | 28433247 | -1.80 | -3.46 | -0.15 | 0.29 | 0.12 | 0.69 |
| Lower gastrointestinal haemorrhage | 5 | 27010 | 17911 | 28435037 | -1.67 | -3.32 | -0.01 | 0.31 | 0.13 | 0.75 |
| Spontaneous haematoma | 5 | 27010 | 681 | 28452267 | 2.26 | 0.60 | 3.91 | 4.80 | 1.99 | 11.56 |
| Systemic inflammatory response syndrome | 5 | 27010 | 5252 | 28447696 | 0.00 | -1.65 | 1.66 | 1.00 | 0.42 | 2.41 |
| Traumatic haematoma | 5 | 27010 | 1171 | 28451777 | 1.77 | 0.11 | 3.42 | 3.41 | 1.42 | 8.22 |
| Venoocclusive liver disease | 5 | 27010 | 6284 | 28446664 | -0.23 | -1.89 | 1.42 | 0.85 | 0.35 | 2.04 |
| Ventricular extrasystoles | 5 | 27010 | 6328 | 28446620 | -0.24 | -1.90 | 1.41 | 0.85 | 0.35 | 2.03 |
| Ventricular hypokinesia | 5 | 27010 | 1933 | 28451015 | 1.23 | -0.42 | 2.89 | 2.36 | 0.98 | 5.67 |
| Withdrawal hypertension | 5 | 27010 | 175 | 28452773 | 3.04 | 1.38 | 4.69 | 8.26 | 3.39 | 20.09 |
| Cardiorenal syndrome | 4 | 27011 | 241 | 28452707 | 2.62 | 0.72 | 4.52 | 6.17 | 2.30 | 16.59 |
| Circulatory collapse | 4 | 27011 | 6456 | 28446492 | -0.56 | -2.46 | 1.34 | 0.68 | 0.25 | 1.81 |
| Coronary artery occlusion | 4 | 27011 | 6037 | 28446911 | -0.47 | -2.37 | 1.43 | 0.72 | 0.27 | 1.92 |
| Diarrhoea haemorrhagic | 4 | 27011 | 3907 | 28449041 | 0.10 | -1.80 | 2.00 | 1.07 | 0.40 | 2.85 |
| Haemorrhage intracranial | 4 | 27011 | 13832 | 28439116 | -1.60 | -3.50 | 0.30 | 0.33 | 0.12 | 0.88 |
| Hypersensitivity vasculitis | 4 | 27011 | 3674 | 28449274 | 0.17 | -1.73 | 2.07 | 1.13 | 0.42 | 3.01 |
| Hypertensive heart disease | 4 | 27011 | 906 | 28452042 | 1.72 | -0.18 | 3.62 | 3.31 | 1.24 | 8.84 |
| Hypovolaemic shock | 4 | 27011 | 2723 | 28450225 | 0.54 | -1.36 | 2.44 | 1.46 | 0.55 | 3.89 |
| Injection site bruising | 4 | 27011 | 29566 | 28423382 | -2.67 | -4.57 | -0.77 | 0.16 | 0.06 | 0.42 |
| Kounis syndrome | 4 | 27011 | 1555 | 28451393 | 1.19 | -0.71 | 3.09 | 2.28 | 0.85 | 6.08 |
| Mitral valve incompetence | 4 | 27011 | 3874 | 28449074 | 0.11 | -1.79 | 2.01 | 1.08 | 0.40 | 2.87 |
| Myocardial ischaemia | 4 | 27011 | 5835 | 28447113 | -0.42 | -2.32 | 1.48 | 0.74 | 0.28 | 1.99 |
| Papilloedema | 4 | 27011 | 2147 | 28450801 | 0.82 | -1.08 | 2.72 | 1.77 | 0.66 | 4.73 |
| Pulmonary artery stenosis | 4 | 27011 | 312 | 28452636 | 2.49 | 0.59 | 4.39 | 5.65 | 2.11 | 15.15 |
| Retinal haemorrhage | 4 | 27011 | 4026 | 28448922 | 0.06 | -1.84 | 1.96 | 1.04 | 0.39 | 2.78 |
| Retinal vein occlusion | 4 | 27011 | 1902 | 28451046 | 0.96 | -0.94 | 2.86 | 1.95 | 0.73 | 5.21 |
| Small intestinal haemorrhage | 4 | 27011 | 2743 | 28450205 | 0.54 | -1.36 | 2.44 | 1.45 | 0.54 | 3.87 |
| Traumatic haemorrhage | 4 | 27011 | 1204 | 28451744 | 1.45 | -0.45 | 3.35 | 2.74 | 1.03 | 7.31 |
| Ulcer haemorrhage | 4 | 27011 | 5947 | 28447001 | -0.45 | -2.35 | 1.45 | 0.73 | 0.27 | 1.95 |
| Urinary bladder haemorrhage | 4 | 27011 | 2371 | 28450577 | 0.71 | -1.19 | 2.61 | 1.64 | 0.61 | 4.36 |
| Varices oesophageal | 4 | 27011 | 2040 | 28450908 | 0.88 | -1.02 | 2.78 | 1.85 | 0.69 | 4.93 |
| Ventricular arrhythmia | 4 | 27011 | 2614 | 28450334 | 0.59 | -1.31 | 2.49 | 1.51 | 0.57 | 4.02 |
| Arteriosclerosis | 3 | 27012 | 2672 | 28450276 | 0.20 | -2.09 | 2.49 | 1.15 | 0.37 | 3.58 |
| Atrioventricular block complete | 3 | 27012 | 3191 | 28449757 | -0.01 | -2.30 | 2.28 | 0.99 | 0.32 | 3.08 |
| Cardiogenic shock | 3 | 27012 | 4842 | 28448106 | -0.54 | -2.83 | 1.75 | 0.69 | 0.22 | 2.13 |
| Cardiopulmonary failure | 3 | 27012 | 1655 | 28451293 | 0.76 | -1.53 | 3.05 | 1.69 | 0.54 | 5.25 |
| Cyanosis | 3 | 27012 | 4135 | 28448813 | -0.34 | -2.63 | 1.95 | 0.79 | 0.25 | 2.45 |
| Disseminated intravascular coagulation | 3 | 27012 | 5075 | 28447873 | -0.60 | -2.89 | 1.69 | 0.66 | 0.21 | 2.04 |
| Embolic cerebral infarction | 3 | 27012 | 680 | 28452268 | 1.61 | -0.68 | 3.90 | 3.06 | 0.98 | 9.50 |
| Embolism | 3 | 27012 | 5676 | 28447272 | -0.75 | -3.04 | 1.54 | 0.59 | 0.19 | 1.84 |
| Gastric ulcer haemorrhage | 3 | 27012 | 4897 | 28448051 | -0.56 | -2.85 | 1.73 | 0.68 | 0.22 | 2.11 |
| Granulomatosis with polyangiitis | 3 | 27012 | 507 | 28452441 | 1.83 | -0.46 | 4.12 | 3.57 | 1.15 | 11.10 |
| Haemorrhagic transformation stroke | 3 | 27012 | 1725 | 28451223 | 0.71 | -1.58 | 3.00 | 1.64 | 0.53 | 5.08 |
| Haemothorax | 3 | 27012 | 2354 | 28450594 | 0.36 | -1.93 | 2.65 | 1.28 | 0.41 | 3.97 |
| Henoch-schonlein purpura | 3 | 27012 | 907 | 28452041 | 1.36 | -0.93 | 3.65 | 2.57 | 0.83 | 7.99 |
| Increased tendency to bruise | 3 | 27012 | 4647 | 28448301 | -0.49 | -2.78 | 1.80 | 0.71 | 0.23 | 2.21 |
| Localised oedema | 3 | 27012 | 1831 | 28451117 | 0.64 | -1.65 | 2.93 | 1.56 | 0.50 | 4.85 |
| Peripheral artery occlusion | 3 | 27012 | 1334 | 28451614 | 0.99 | -1.31 | 3.28 | 1.98 | 0.64 | 6.15 |
| Peripheral coldness | 3 | 27012 | 6071 | 28446877 | -0.84 | -3.13 | 1.45 | 0.56 | 0.18 | 1.73 |
| Petechiae | 3 | 27012 | 4896 | 28448052 | -0.56 | -2.85 | 1.73 | 0.68 | 0.22 | 2.11 |
| Phlebitis | 3 | 27012 | 2834 | 28450114 | 0.13 | -2.16 | 2.42 | 1.10 | 0.35 | 3.40 |
| Portal vein thrombosis | 3 | 27012 | 1998 | 28450950 | 0.55 | -1.74 | 2.84 | 1.46 | 0.47 | 4.53 |
| Post procedural haematoma | 3 | 27012 | 1610 | 28451338 | 0.79 | -1.50 | 3.08 | 1.73 | 0.56 | 5.36 |
| Pulmonary alveolar haemorrhage | 3 | 27012 | 3979 | 28448969 | -0.29 | -2.58 | 2.00 | 0.82 | 0.26 | 2.54 |
| Purpura senile | 3 | 27012 | 46 | 28452902 | 2.68 | 0.39 | 4.97 | 6.44 | 2.00 | 20.70 |
| Renal artery arteriosclerosis | 3 | 27012 | 126 | 28452822 | 2.49 | 0.20 | 4.78 | 5.65 | 1.80 | 17.75 |
| Right ventricular failure | 3 | 27012 | 7423 | 28445525 | -1.11 | -3.40 | 1.18 | 0.46 | 0.15 | 1.44 |
| Scleroderma renal crisis | 3 | 27012 | 259 | 28452689 | 2.23 | -0.06 | 4.52 | 4.69 | 1.50 | 14.65 |
| Sinoatrial block | 3 | 27012 | 466 | 28452482 | 1.89 | -0.40 | 4.18 | 3.71 | 1.19 | 11.56 |
| Subarachnoid haematoma | 3 | 27012 | 108 | 28452840 | 2.53 | 0.24 | 4.82 | 5.81 | 1.84 | 18.30 |
| Subcutaneous haematoma | 3 | 27012 | 1590 | 28451358 | 0.80 | -1.49 | 3.09 | 1.74 | 0.56 | 5.41 |
| Temporal arteritis | 3 | 27012 | 1276 | 28451672 | 1.03 | -1.26 | 3.32 | 2.05 | 0.66 | 6.35 |
| Thrombophlebitis superficial | 3 | 27012 | 3343 | 28449605 | -0.07 | -2.36 | 2.22 | 0.95 | 0.31 | 2.96 |
| Thrombotic thrombocytopenic purpura | 3 | 27012 | 2941 | 28450007 | 0.09 | -2.20 | 2.38 | 1.06 | 0.34 | 3.30 |
| Tricuspid valve incompetence | 3 | 27012 | 3032 | 28449916 | 0.05 | -2.24 | 2.34 | 1.04 | 0.33 | 3.21 |
| Varicose vein | 3 | 27012 | 3380 | 28449568 | -0.08 | -2.37 | 2.21 | 0.94 | 0.30 | 2.93 |
| Vascular stent thrombosis | 3 | 27012 | 3609 | 28449339 | -0.17 | -2.46 | 2.12 | 0.89 | 0.29 | 2.77 |
| Vena cava thrombosis | 3 | 27012 | 1506 | 28451442 | 0.86 | -1.43 | 3.15 | 1.81 | 0.58 | 5.63 |
| Venous thrombosis | 3 | 27012 | 3565 | 28449383 | -0.15 | -2.44 | 2.14 | 0.90 | 0.29 | 2.79 |
| Vitreous haemorrhage | 3 | 27012 | 2663 | 28450285 | 0.21 | -2.08 | 2.50 | 1.16 | 0.37 | 3.59 |

**Table S7.** Disproportionality results of cardiac and vascular toxicities associated with dexlansoprazole

| PT | a | b | c | d | IC | IC_025_ | IC_975_ | ROR | ROR_025_ | ROR_975_ |
| --- | --- | --- | --- | --- | --- | --- | --- | --- | --- | --- |
| Renal haemangioma | 221 | 30301 | 1861 | 28447580 | 6.34 | 6.12 | 6.56 | 89.23 | 77.58 | 102.63 |
| Thrombophlebitis superficial | 9 | 30513 | 3337 | 28446104 | 1.22 | 0.04 | 2.39 | 2.33 | 1.21 | 4.48 |
| Renal artery stenosis | 5 | 30517 | 683 | 28448758 | 2.15 | 0.50 | 3.81 | 4.46 | 1.85 | 10.76 |
| Renal hypertension | 4 | 30518 | 371 | 28449070 | 2.32 | 0.42 | 4.22 | 5.01 | 1.87 | 13.42 |
